# Identifying the Mechanism of Polygoni Cuspidati Rhizoma et Radix in Treating Acute Liver Failure Based on Network Pharmacology and Molecular Docking

**DOI:** 10.1155/2022/2021066

**Published:** 2022-04-08

**Authors:** Jing Hong, Jie Ding, Han-han Hong, Xiao-wan Xu, Bo Pan, Yi Ruan, Xiao-feng Zhai

**Affiliations:** ^1^Department of Integrative Oncology, Changhai Hospital, Naval Medical University, Shanghai 200433, China; ^2^School of Traditional Chinese Medicine, Naval Medical University, Shanghai 200433, China; ^3^Gynecology of Traditional Chinese Medicine, Changhai Hospital, Naval Medical University, Shanghai 200433, China; ^4^Department of Nursing, Chengjiaqiao Community Health Service Center of Changning District, Shanghai 201103, China

## Abstract

**Materials and Methods:**

The potential bioactive compounds of PCRR and their targets were collected from TCMSP, TCMID, and BATMAN-TCM databases with absorption, distribution, metabolism, and excretion protocols (oral bioavailability ≥30% and drug-likeness ≥0.18). The ALF-related target genes were identified using the GeneCards and OMIM databases. A protein-protein interaction (PPI) network among these targets was constructed using the Cytoscape software to obtain the core targets. The genes associated with ALF were analyzed via Gene Ontology (GO) and Kyoto Encyclopedia of Genes and Genomes (KEGG) enrichment analyses to identify the signaling pathways related to the therapeutic effect of PCRR in ALF.

**Results:**

In total, 10 bioactive compounds of PCRR and 200 targets related to them were obtained, and 2913 ALF-related target genes were identified. PPI network analysis pinpointed 15 core targets, namely, TP53, AKT1, JUN, HSP90AA1, MAPK1, RELA, TNF, ESR1, IL6, MYC, MAPK14, FOS, RB1, CDKN1A, and EGFR. GO enrichment and KEGG pathway analyses revealed that the therapeutic mechanisms of PCRR in ALF are related to cell metabolism, oxidative stress, inflammation, and hepatocyte apoptosis.

**Conclusion:**

This is the first study to explore the therapeutic mechanisms of PCRR in ALF via network pharmacology and molecular docking. This study provides a research platform with candidate ALF-related targets of PRCC for the development of therapeutics against ALF.

## 1. Introduction

Acute liver failure (ALF) is a serious decompensation disorder caused by various factors, including hepatic synthesis, detoxification, excretion, and biotransformation [1]. In developed countries, the incidence of ALF is higher than 10 cases per million persons per year [2]. Hepatitis virus infection and acetaminophen are the main causes of ALF in developing [3] and developed countries [4], respectively. Although the worldwide survival rate in ALF has steadily improved from approximately 20% to more than 60% over the past few decades [5], there are still no specific drugs for the treatment of this disorder.

Traditional Chinese medicine (TCM) uses natural sources and thereby provides unique advantages in the treatment of liver injury [6]. Polygoni Cuspidati Rhizoma et Radix (PCRR) is a popular Chinese herb used to treat various liver diseases. PCRR has been reported to have more than 67 bioactive components, including quinones, stilbenes, flavonoids, and lignans [7]. Acute-on-chronic liver failure refers to acute decompensation in liver injury and has a similar prognosis as ALF [8, 9]. The Guidelines for Clinical Diagnosis and Treatment of Acute-on-chronic Liver Failure in TCM recommends PCRR as one of the main components of the prescription in treating acute-on-chronic liver failure [10]. The results of many clinical observations are in line with this recommendation [11, 12]. A study has confirmed the protective effect of the PCRR against carbon tetrachloride-induced liver injury in mice [13]. However, only a few studies on the therapeutic mechanisms of PCRR in ALF have been reported.

The therapeutics of TCM generally involve multiple components, targets, and pathways, and thus characterization of therapeutic mechanisms is highly challenging in TCM. Network pharmacology is very useful to this end. In this approach, a multilevel network of “disease/phenotype–gene/drug” is constructed to explore the correlation between drugs and diseases from a holistic perspective, whereby drug targets can be identified or new drugs can be developed [14, 15].

**Figure 1:**
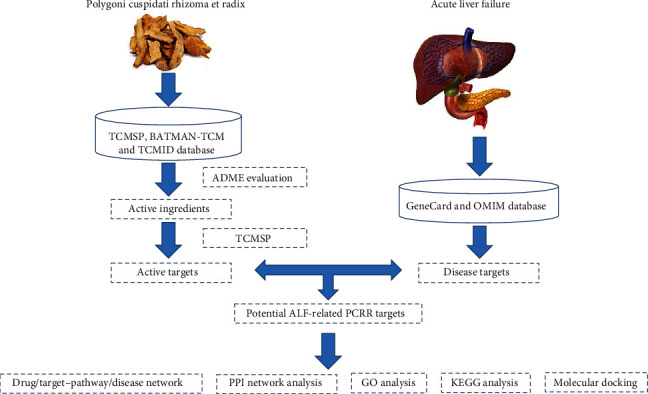
Detailed flowchart of the study design.

This study sought to identify the bioactive compounds of PCRR against ALF and the involving key genes and pathways via network pharmacology and molecular docking methods. The flowchart of this study is shown in Figure 1.

## 2. Materials and Methods

### 2.1. Collection of Potential Bioactive Compounds and Related Targets of PCRR

The corresponding compounds and related information were obtained using the Traditional Chinese Medicine Systems Pharmacology (TCMSP, https://tcmspw.com/tcmsp.php) database [16], Bioinformatics Analysis Tool for Molecular mechANism of Traditional Chinese Medicine (BATMAN-TCM, http://bionet.ncpsb.org/batman-tcm/) [17], and Traditional Chinese Medicine Integrated Database (TCMID, http://www.megabionet.org/tcmid/) [18]. TCMSP also provides absorption, distribution, metabolism, and excretion (ADME)-related parameters, such as oral bioavailability (OB) and drug-likeness (DL), of herbal components. OB indicates the relative amount and rate of oral absorption of a drug into the circulation of the body. DL is a concept based on the physicochemical properties and molecular structure of existing drugs. Generally, only compounds with OB ≥30% and DL ≥0.18 are considered potential bioactive compounds [19]. The target information analysis function of the TCMSP platform was used to obtain the gene targets of the anti-ALF bioactive components of PCRR. For the components with no corresponding targets in the TCMSP platform, a similarity ensemble approach (SEA, https://sea.bkslab.org/) was used to predict the targets. The target protein species was set as *Homo sapiens*, and the obtained target information was unified using UniProt (https://www.uniprot.org).

### 2.2. Acquisition of ALF-Related Targets

Keywords such as “acute liver failure”, “acute hepatic failure”, and “ALF” were used to search ALF-related targets from the GeneCards (https://www.genecards.org) [20] and OMIM (https://omim.org/) [21] databases. PCRR-related targets and ALF-related targets were input into an online Venn tool (https://bioinfogp.cnb.csic.es/tools/venny/) to obtain the intersection genes, which were considered candidate targets of PCRR against ALF

### 2.3. Analysis of the Drug/Target–Pathway/Disease Network

The relationship between potential bioactive compounds of PRCC and intersection genes was constructed using the Cytoscape software (version 3.8.0) as a drug-components-target-disease network. The average value of the degree value of the network nodes was calculated (average value), and the components with the degree value of the network node ≥ average value were considered as core components.

### 2.4. Gene Ontology (GO) and Kyoto Encyclopedia of Genes and Genomes (KEGG) Analyses

The candidate targets of PCRR against ALF obtained were used to explore the potential mechanism of PCRR in ALF via GO and KEGG analyses. The GO and KEGG pathway enrichment analyses were performed using the Database for Annotation, Visualization, and Integrated Discovery tool (DAVID, https://david.ncifcrf.gov/home.jsp). The biological processes (BPs), cellular components (CCs), molecular functions (MFs), and key signaling pathways were obtained to explore PCRR-related biological pathways. The functional annotations with *P*-values < 0.05 were further analyzed.

### 2.5. Construction of a Protein-Protein Interaction (PPI) Network

Search Tool for the Retrieval of Interacting Genes (STRING, https://string-db.org/) was used to identify possible PPIs by uploading the candidate targets from the Venn diagram. Species was limited to *Homo sapiens* with a confidence score > 0.9. The analysis plugin of Cytoscape 3.8.0 was used to visualize the PPI network, in which the target of the height value plays a pivotal role. The HUBBA plug-in was used to calculate the degree of hub nodes and to select out hub nodes with degree higher than the average degree as the core targets.

### 2.6. Molecular Docking Simulation

The top 15 target genes were selected. The protein crystal structures corresponding to the core target genes were accessed from the Protein Data Bank (PDB, https://www.rcsb.org) database, and the structures of the bioactive components were downloaded from the TCMSP database. The AutoDock 4.2.6 software was employed to perform molecular docking between receptors and ligands. Eventually, the results were visualized using the PyMOL software.

## 3. Results

### 3.1. Bioactive Compounds and Potential Targets of PCRR

After searching, filtering, and duplicate removal in the TCMSP, TCMID, and BATMAN-TCM databases, 10 bioactive components of PCRR with OB ≥ 30% and DL ≥ 0.18 were collected, including luteolin, quercetin, *β*-sitosterol, (+)-catechin, physcion diglucoside, rhein, torachrysone-8-O-*β*-D-(6′-oxayl)-glucoside, 6,8-dihydroxy-7-methoxyxanthone, physovenine, and picralinal (Table 1). Additionally, 200 target genes interacting with these 10 bioactive components were identified (Supplementary file, Table S1).

### 3.2. Potential ALF-Related PCRR Targets

**Figure 2:**
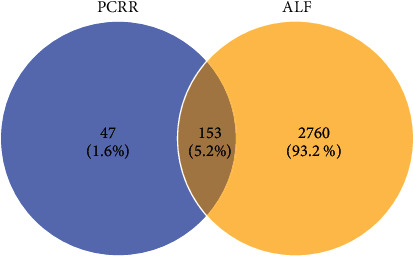
The candidate target genes of Polygoni Cuspidati Rhizoma et Radix (PCRR) and/or in acute liver failure (ALF).

In total, 2913 ALF-related target genes were obtained by searching the GeneCards and OMIM databases (Supplementary file, Table S2). The Venn diagram tool was used to identify the genes found among both ALF-related targets and PCRR targets. Consequently, 153 ALF-related PCRR target candidates were identified (Figure 2 and Supplementary file, Table S3).

### 3.3. Analysis of the Drug/Target–Pathway/Disease Network

**Figure 3:**
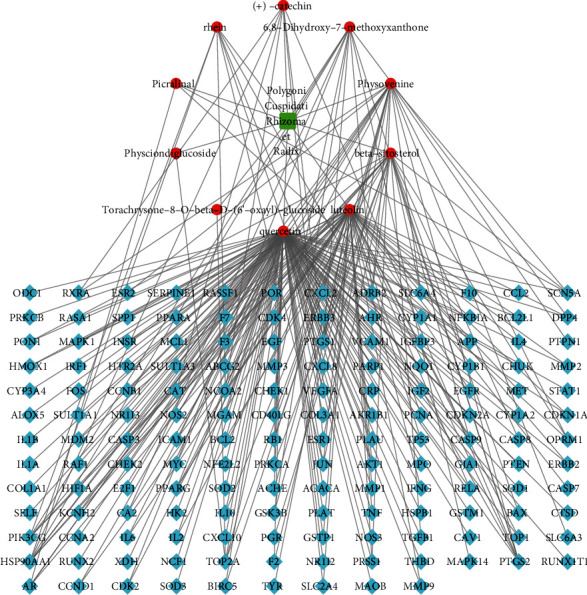
Drug-components–target genes network. The red circle nodes represent the bioactive components of Polygoni Cuspidati Rhizoma et Radix (PCRR), the blue diamond-shaped nodes represent the candidate targets, and the green square represents PCRR.

The 10 bioactive components of PCRR and 153 candidate targets of PCRR against ALF were imported into the Cytoscape 3.8.0 software to illustrate the interaction between the two groups (Figure 3). We identified the core components among the 153 ALF-related PCRR target candidates by calculating the degree values of the network nodes. In the order from high to low degrees, the core components were quercetin (degree = 131), luteolin (degree = 51), *β*-sitosterol (degree = 22), and physovenine (degree = 22) (Table 2). According to the network analysis, multiple bioactive components of PCRR act on at least one core target gene. The results showed that the therapeutic effect of PCRR in ALF has multicomponent and multitarget characteristics.

### 3.4. GO Functional and KEGG Pathway Enrichment Analysis

**Figure 4:**
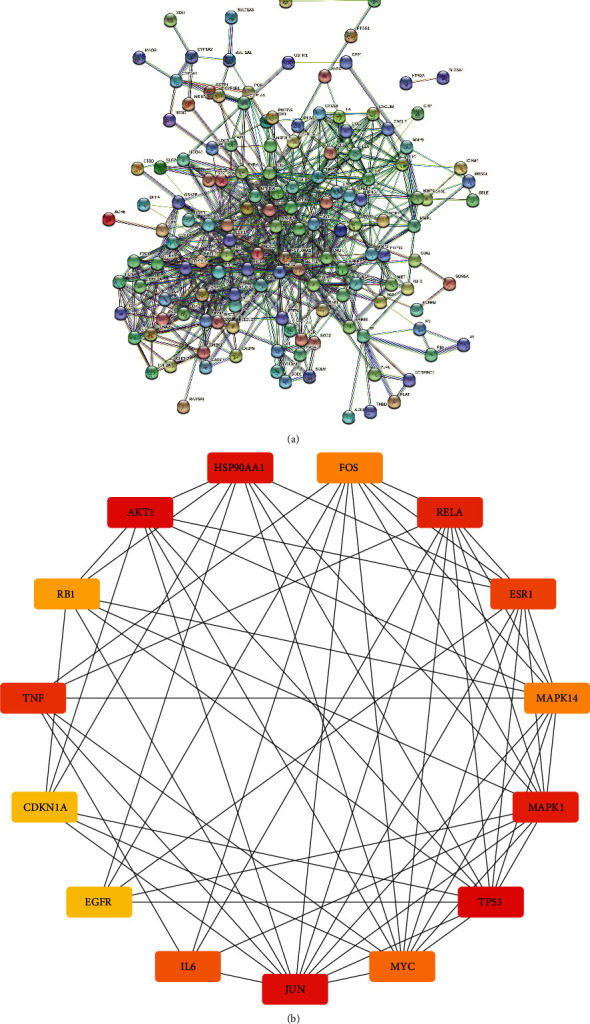
Protein-protein interaction (PPI) network based on the candidate target genes of Polygoni Cuspidati Rhizoma et Radix against acute liver failure. (a) PPI network of the candidate target genes. Each node represents the protein product of an associated target gene. The degree values of the proteins are represented by the node sizes. Colors indicate the connection sources. (b) The top 15 core target genes were identified based on the degree values. The protein with the darkest color has the highest degree value, indicating that it plays the most significant role in the regulation of the network.

To elucidate the biological processes involved in the ALF-related PCRR candidates targets, GO enrichment analysis was performed. A total of 320 significantly enriched GO terms were identified (*P*-value <0.05, Supplementary file, Table S4). The top 10 significantly enriched terms, including BPs, MFs, and CCs are presented in Figure 4(a). In the order from low to high adjusted *P*-values, the top three GO-MC terms were mainly enriched in protein domain-specific binding (GO:0019904), steroid hormone receptor activity (GO:0003707), and scaffold protein binding (GO:0097110); the top three GO-CC terms were mainly enriched in cytoplasm (GO:0005737), mast cell granule (GO:0042629), and condensed chromosome (GO:0000793); and the top three GO-BP terms were mainly enriched in positive regulation of blood vessel endothelial cell migration (GO:0043536), positive regulation of mitotic cell cycle (GO:0045931), and positive regulation of transcription from RNA polymerase II promoter (GO:0045944).

KEGG enrichment analysis was performed to elucidate the pathways involved in the therapeutic effect of PCRR in the treatment of ALF. Consequently, 160 enriched KEGG pathways were identified (*P*-value <0.05, Supplementary file, Table S5). The top 30 significant signaling pathways are shown in Figure 4(b). The top 10 ALF-related signaling pathways were identified as pathway in cancer (path:hsa05200), AGE-RAGE signaling pathway in diabetic complications (path:hsa04933), hepatitis B (path:hsa05161), prostate cancer (path:hsa05215), bladder cancer (path:hsa05219), fluid shear stress and atherosclerosis (path:hsa05418), interlukin (IL)-17 signaling pathway (path:hsa04657), Kaposi sarcoma-associated herpesvirus infection (path:hsa05167), pancreatic cancer (path:hsa05212), and tumor necrosis factor (TNF) signaling pathway (path:hsa04668). These pathways suggest that the therapeutic effect of PCRR in ALF is related to cell metabolism, oxidative stress, inflammation, and hepatocyte apoptosis.

### 3.5. PPI Network Analysis

**Figure 5:**
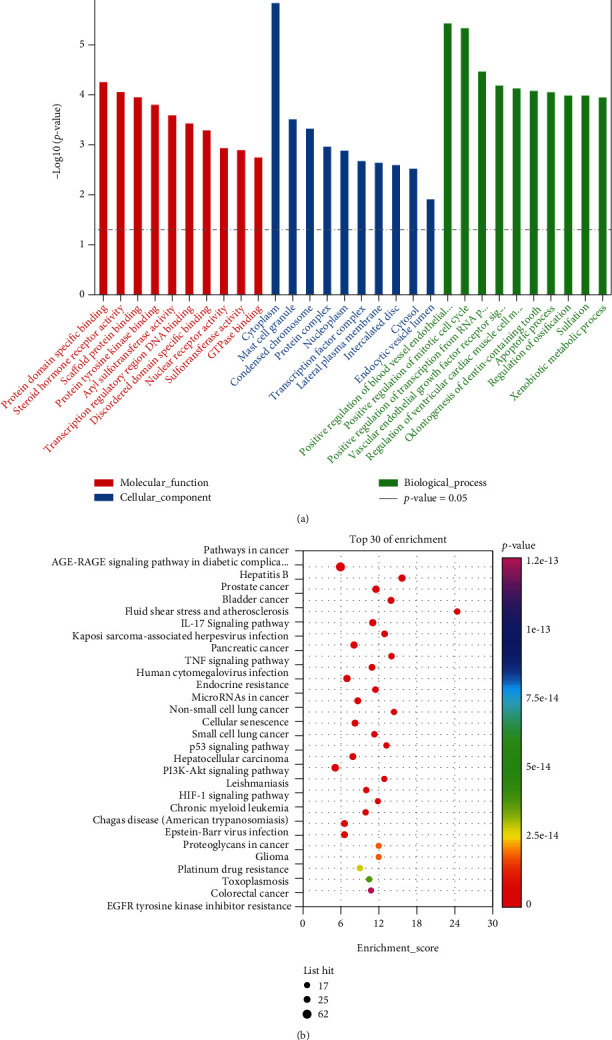
Gene ontology (GO) and Kyoto Encyclopedia of Genes and Genomes (KEGG) analyses. Different colors represent different *P*-values, and circle size represents the counts. (a) The top 10 GO terms. Red, blue, and green bars represent molecular function, cellular component, and biological process, respectively. (b) The top 30 KEGG pathways.

To assess the synergism between the bioactive components of PCRR, the 153 candidate target genes were imported into the STRING database to construct an initial PPI network with the minimum required interaction score > 0.9 (Figure 5(a)). The Cytoscape 3.8.0 software was used to reconstruct the STRING graph, and the HUBBA plug-in was used to select the top 15 targets for plotting (Figure 5(b)). The core targets, which may play important anti-ALF roles, were TP53, AKT1, JUN, HSP90AA1, MAPK1, RELA, TNF, ESR1, IL6, MYC, MAPK14, FOS, RB1, CDKN1A, and EGFR (Table 3).

### 3.6. Validation through Molecular Docking

**Figure 6:**
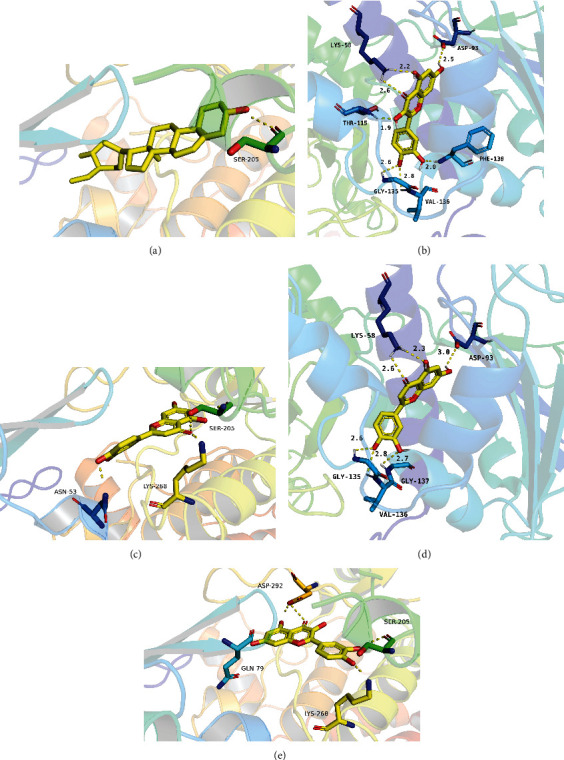
Molecular docking of the receptors and their ligands: (a) *β*-sitosterol to AKT1; (b) quercetin to HSP90AA1; (c) luteolin to AKT1; (d) luteolin to HSP90AA1; (e) quercetin to AKT1.

Molecular docking is used to verify the interaction between ligands and their receptors. Here, we applied this strategy for the 4 bioactive compounds of PCRR and the 15 core target genes by using AutoDock Vina (Table 4). A minimum binding potential energy of < 0 between a molecule and its target indicates that the two molecules can spontaneously bind to each other [22]. The lowest binding-free energies of *β*-sitosterol to AKT1, quercetin to HSP90AA1, luteolin to AKT1, luteolin to HSP90AA1, and quercetin to AKT1 were estimated at –10.9, –10.2, –9.8, –9.8, and –9.7 kcal/mol, respectively (See Figure 6).

## 4. Discussion

ALF is a rare but serious clinical syndrome involving hepatocyte damage and progresses rapidly, with a possibility of causing multiple organ dysfunction [23]. For patients with ALF, there is no specific treatment. With the advent of liver transplantation, the survival rate of ALF has greatly improved [24]. However, the lack of donors and high treatment costs limited the application of this approach. PCRR is a classical TCM therapeutic with a highlighted effect in the prevention and treatment of various liver diseases. TCM comprises multicomponent and multitarget therapeutics, which are difficult to mechanistically characterize. Network pharmacology is a simple and feasible method that solves this difficulty. In this study, the bioactive components and potential targets of PCRR in the treatment of ALF were predicted via network pharmacology and molecular docking.

According to ADME protocols (OB ≥ 30%, DL ≥ 0.18) and the principle of target correspondence, four bioactive components were screened out. Of them, the flavonoid luteolin is found in various types of plants, including fruits, vegetables, and herbs, worldwide [25]. Previous studies have suggested that the protective effect of luteolin on acetaminophen-induced liver failure in mice may be related to the inhibition of lipid peroxidation, oxidative stress, and estrogen-receptor stress [26, 27]. Quercetin is a bioactive flavonoid in the class of polyphenols [28], which can prevent and treat liver injury by preventing oxidative stress, inhibiting the release of inflammatory factors, and promoting the synthesis of antioxidant enzymes [29, 30].

Based on the PPI network analysis, we predicted that the ALF-related genes most commonly targeted by the PCRR bioactive compounds are TP53, AKT1, JUN, HSP90AA1, MAPK1, RELA, TNF, ESR1, IL6, MYC, MAPK14, FOS, RB1, CDKN1A, and EGFR. The tumor suppressor gene TP53 encodes P53 [31, 32], whose transient activation helps prevent progression of acetaminophen-induced liver injury, and continued activation of P53 may affect regeneration and recovery of the liver [33, 34]. AKT1 has been reported to regulate fibrogenesis and proliferation in hepatocytes and hepatic stellate cells [35, 36]. Additionally, previous studies have shown that HSP90 can promote proinflammatory cytokines and its inhibition can attenuate alcohol-induced liver injury [37, 38]. MAPK1 (extracellular signal-regulated kinase 2, ERK2) is involved in the regulation of cellular physiology and pathology [39]. Altering the ERK signaling pathway through ERK2 deficiency can reduce liver fibrosis and inflammation [40]. ESR1-mediated signaling inhibits liver regeneration after chemical-induced liver injury by suppressing the Wnt signaling pathway, resulting in lower cyclin D1 activation [41]. During the development of acute liver failure, TNF-mediated over-immune cascade response may contribute to massive hepatocyte apoptosis and impaired hepatocyte proliferation [42, 43].

To explore the therapeutic mechanism of PCRR in ALF, GO and KEGG pathway enrichment analyses were performed. According to the adjusted *P*-values, the top three GO-MC terms were mainly enriched in protein domain-specific binding, steroid hormone receptor activity, and scaffold protein binding; the top three GO-CC terms were mainly enriched in cytoplasm, mast cell granule, and condensed chromosome; and the top three GO-BP terms were mainly enriched in positive regulation of blood vessel endothelial cell migration, positive regulation of mitotic cell cycle, and positive regulation of transcription from RNA polymerase II promoter. The 10 crucial pathways that may be regulated by PCRR in the treatment of ALF by the KEGG pathway enrichment analysis included pathway in cancer, AGE-RAGE pathway in diabetic complications, hepatitis B, prostate cancer, bladder cancer, fluid shear stress and atherosclerosis, IL-17 pathway, Kaposi sarcoma-associated herpesvirus infection, pancreatic cancer, and TNF. The pathway enrichment results suggested that the anti-ALF therapeutic effect of PCRR mainly results from the regulation of immune and inflammatory responses and cell metabolism. Cancer mechanisms are known to be relevant with ALF since neoplastic infiltration is one of the courses of ALF progression [44–46]. Chronic hepatitis B virus infection is one of the important causes of acute liver failure in developing countries, including China [47]. AGE-RAGE interaction contributes to fat accumulation in the liver, increases oxidative stress and chronic inflammation, and may be involved in liver injury [48–50]. IL-17 plays an important role in the pathogenesis of immune-mediated liver injury; IL-17 is significantly upregulated in the liver and serum of BALB/cJ mice infected with mouse hepatitis virus strain 3 [51]. The PI3K-Akt signaling affects cell migration, mobilization, differentiation, and apoptosis [52, 53] and has also been found to affect early liver regeneration and improve survival in a mouse model of acetaminophen-induced acute liver injury [52, 54]. Excessive reactive oxygen species (ROS) can directly lead to oxidative stress, which plays an important role in liver damage [55]. Activation of the PI3K/Akt signaling can alleviate liver injury by reducing ROS levels, inhibiting apoptosis, and accelerating hyoxia-inducible factor-1*α* [56].

## 5. Conclusion

This is the first study that has predicted the therapeutic mechanisms of PCRR in ALF by using network pharmacology and molecular docking. The results suggest that the therapeutic effect of PCRR in ALF involves multiple components, targets, and pathways. Luteolin, quercetin, *β*-sitosterol, and physovenine are likely the major bioactive compounds of PCRR against ALF. Accordingly, this study provides a research platform with candidate ALF-related targets of PRCC for the development of therapeutics against ALF. However, it has several limitations as well. First, the potential bioactive components are screened primarily by databases using ADME protocols [58], and some components may be overlooked. Second, the study lacks experimental verification, which should be addressed in biologically relevant platforms in the future.

## Figures and Tables

**Table 1:** Active pharmaceutical components of PCRR.

Molecule ID	Molecule name	OB	DL
MOL000006	Luteolin	36.16	0.25
MOL000098	Quercetin	46.43	0.28
MOL000358	*β*-Sitosterol	36.91	0.75
MOL000492	(+)-Catechin	54.83	0.24
MOL002259	Physcion diglucoside	41.65	0.63
MOL002268	Rhein	47.07	0.28
MOL002280	Torachrysone-8-O-*β*-D-(6-oxayl)-glucoside	43.02	0.74
MOL013281	6,8-Dihydroxy-7-methoxyxanthone	35.83	0.21
MOL013287	Physovenine	106.10	0.19
MOL013288	Picralinal	58.01	0.75

PCRR: Polygoni Cuspidati Rhizoma et Radix; OB: oral bioavailability; DL: drug-likeness.

**Table 2:** Core pharmaceutical components of PCRR.

Molecule ID	Molecule name	OB	DL	2D structure	PubChem CID
MOL000006	Luteolin	36.16	0.245	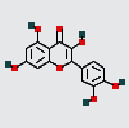	5280445
MOL000098	Quercetin	46.43	0.28	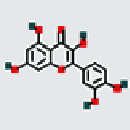	5280343
MOL000358	*β*-Sitosterol	36.91	0.75	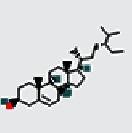	222284
MOL013287	Physovenine	106.21	0.19	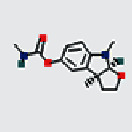	442113

PCRR: Polygoni Cuspidati Rhizoma et Radix; OB: oral bioavailability; DL: drug-likeness.

**Table 3:** Core targets of PCRR in the treatment of ALF and the topological parameters.

Uniport ID	Gene symbol	Degree	Betweenness	Closeness
P04637	TP53	40	0.11	0.49
P31749	AKT1	39	0.11	0.49
P05412	JUN	36	0.09	0.50
P07900	HSP90AA1	35	0.08	0.48
P28482	MAPK1	34	0.12	0.50
Q04206	RELA	33	0.06	0.49
P01375	TNF	28	0.06	0.46
P03372	ESR1	26	0.04	0.46
P05231	IL6	25	0.04	0.44
P01106	MYC	24	0.03	0.46
Q16539	MAPK14	23	0.02	0.46
P01100	FOS	23	0.04	0.46
P06400	RB1	22	0.02	0.45
P38936	CDKN1A	21	0.01	0.43
P00533	EGFR	21	0.03	0.44

PCRR: Polygoni Cuspidati Rhizoma et Radix; ALF: acute liver failure.

**Table 4:** Results of 15 core target genes and related bioactive compounds of molecular docking.

No.	Targets	PDB ID	Compound	Binding affinity(kcal/Mol)
1	AKT1	3O96	Luteolin	-9.8
			Physovenine	-8.6
			Quercetin	-9.7
			*β*-Sitosterol	-10.9
2	CDKN1A	6P8H	Luteolin	-6.4
			Physovenine	-5.9
			Quercetin	-6.0
			*β*-Sitosterol	-6.9
3	EGFR	1 M17	Luteolin	-8.4
			Physovenine	-7.2
			Quercetin	-8.5
			*β*-Sitosterol	-8.5
4	ESR1	1A52	Luteolin	-8.7
			Physovenine	-7.6
			Quercetin	-8.4
			*β*-Sitosterol	-4.2
5	FOS	1A02	Luteolin	-5.6
			Physovenine	-4.9
			Quercetin	-5.0
			*β*-Sitosterol	-5.5
6	HSP90AA1	7L7I	Luteolin	-9.8
			Physovenine	-8.0
			Quercetin	-10.2
			*β*-Sitosterol	-7.2
7	IL-6	1ALU	Luteolin	-8.0
			Physovenine	-6.4
			Quercetin	-7.9
			*β*-Sitosterol	-6.6
8	JUN	1JNM	Luteolin	-5.4
			Physovenine	-4.8
			Quercetin	-5.4
			*β*-Sitosterol	-5.4
9	MAPK1	1PME	Luteolin	-9.2
			Physovenine	-7.5
			Quercetin	-8.5
			*β*-Sitosterol	-8.8
10	MAPK14	1A9U	Luteolin	-7.5
			Physovenine	-6.9
			Quercetin	-7.2
			*β*-Sitosterol	-8.2
11	MYC	5I4Z	Luteolin	-6.5
			Physovenine	-5.6
			Quercetin	-6.1
			*β*-Sitosterol	-6.9
12	RB1	4EIJ	Luteolin	-8.5
			Physovenine	-7.0
			Quercetin	-8.4
			*β*-Sitosterol	-6.9
13	RELA	1NFI	Luteolin	-7.4
			Physovenine	-6.4
			Quercetin	-7.0
			*β*-Sitosterol	-7.0
14	TNF	1TNF	Luteolin	-7.0
			Physovenine	-5.7
			Quercetin	-6.9
			*β*-Sitosterol	-6.6
15	TP53	TP53	Luteolin	-7.1
			Physovenine	-6.0
			Quercetin	-7.3
			*β*-Sitosterol	-6.0

## Data Availability

All data obtained or analyzed during this work are included within the article.

## Abbreviations

ALF: Acute liver failure
PCRR: Polygoni Cuspidati Rhizoma et Radix
TCM: Traditional Chinese medicine
TCMSP: Traditional Chinese medicine systems pharmacology
OB: Oral bioavailability
DL: Drug-likeness
PPI: Protein-protein interaction
GO: Gene ontology
KEGG: Kyoto Encyclopedia of Genes and Genomes.

## Supplementary Materials

Supplementary Materials The 200 target genes interacting with the 10 bioactive components of PCRR, and the 2913 ALF-related target genes are provided in supplementary Tables S1 and S2, respectively. The 153 ALF-related PCRR target candidates are provided in supplementary Table S3. The results of the GO and KEGG pathway enrichment analyses are provided in supplementary Tables S4 and S5, respectively.
