# Supplementary material for: Identifying the Mechanism of Polygoni Cuspidati Rhizoma et Radix in Treating Acute Liver Failure Based on Network Pharmacology and Molecular Docking
Source: Gastroenterol Res Pract. 2022 Apr 8;2022:2021066. doi: 10.1155/2022/2021066 (PMC9012611; doi:10.1155/2022/2021066)
Supplement: Supplementary Materials — The 200 target genes interacting with the 10 bioactive components of PCRR, and the 2913 ALF-related target genes are provided in supplementary Tables S1 and S2, respectively. The 153 ALF-related PCRR target candidates are provided in supplementary Table S3. The results of the GO and KEGG pathway enrichment analyses are provided in supplementary Tables S4 and S5, respectively. [file 2021066.f1.zip › 2021066.f1.pdf]

Supplementary file

Table S1. 200 target genes interacting with the 10 active components of Polygoni Cuspidati Rhizoma et Radix

No.	Gene	No.	Gene	No.	Gene	No.	Gene	No.	Gene
1	ADCY2	41	PIK3CG	81	CXCL8	121	NCF1	161	VEGFA
2	AKT1	42	PPARG	82	CYP1A1	122	NFE2L2	162	XDH
3	APP	43	PRSS1	83	CYP1A2	123	NKX3-1	163	SOD2
4	AR	44	PTGES	84	CYP1B1	124	NOS1,2,3	164	SOD3
5	BCL2L1	45	PTGS1	85	CYP3A4	125	NOS3	165	TOP2B
6	BIRC4	46	PTGS2	86	DCAF5	126	NPEPPS	166	ADRA1A
7	BIRC5	47	RB1	87	DIO1	127	NQO1	167	ADRA1B
8	CASP3	48	RELA	88	DUOX2	128	NR1I2	168	CHRM1
9	CASP7	49	SLC2A4	89	E2F1	129	NR1I3	169	CHRM2
10	CASP9	50	TNF	90	E2F2	130	ODC1	170	CHRM3
11	CCNB1	51	TOP1	91	EGF	131	PARP1	171	CHRM4
12	CCND1	52	TOP2A	92	EIF6	132	PCOLCE	172	CHRNA2
13	CD40LG	53	TP53	93	ELK1	133	PLAT	173	DRD1
14	CDK4	54	TYR	94	ERBB3	134	PLAU	174	GABRA2
15	CDKN1A	55	HSP90AB	95	F10	135	PON1	175	GABRA3
16	DPP4	56	ABCG2	96	F2	136	POR	176	GABRA5
17	EGFR	57	ACACA	97	F3	137	PPARA	177	HTR2A
18	ERBB2	58	ACHE	98	F7	138	PPARD	178	MAP2
19	GSTP1	59	ACP3	99	FOS	139	PRKCA	179	OPRM1
20	HMOX1	60	ADRB2	100	GABRA1	140	PRKCB	180	PDE3A
21	HSP90AA	61	AHR	101	GJA1	141	PSMD3	181	PGR
22	ICAM1	62	AHSA1	102	GSTM1	142	PTEN	182	SLC6A4
23	IFNG	63	AKR1B1	103	GSTM2	143	PTGER3	183	CALM1
24	IL10	64	ALOX5	104	HAS2	144	RAF1	184	CAT
25	IL2	65	BAX	105	HERC5	145	RASA1	185	ESR1
26	IL4	66	BCL2	106	HIF1A	146	RASSF1	186	CA2
27	IL6	67	CASP8	107	HK2	147	RUNX1T1	187	CDK2
28	INSR	68	CAV1	108	HSF1	148	RUNX2	188	CHEK1
29	JUN	69	CCL2	109	HSPB1	149	RXRA	189	GSK3B
30	MAPK1	70	CDKN2A	110	IGF2	150	SCN5A	190	MAPK14
31	MCL1	71	CHEK2	111	IGFBP3	151	SELE	191	PKIA
32	MDM2	72	CHUK	112	IL1A	152	SERPINE1	192	ADRA2B
33	MET	73	CLDN4	113	IL1B	153	SOD1	193	CCNA2
34	MMP1	74	COL1A1	114	IRF1	154	SPP1	194	ESR2
35	MMP2	75	COL3A1	115	KCNH2	155	STAT1	195	GRIA2
36	MMP9	76	CRP	116	MAOB	156	SULT1A1	196	NOS2
37	NCOA2	77	CTSD	117	MGAM	157	SULT1A3	197	OPRD1
38	NFKBIA	78	CXCL10	118	MMP3	158	TGFB1	198	PTPN1
39	NUF2	79	CXCL11	119	MPO	159	THBD	199	SLC6A2
40	PCNA	80	CXCL2	120	MYC	160	VCAM1	200	SLC6A3
