# Supplementary material for: Identifying the Mechanism of Polygoni Cuspidati Rhizoma et Radix in Treating Acute Liver Failure Based on Network Pharmacology and Molecular Docking
Source: Gastroenterol Res Pract. 2022 Apr 8;2022:2021066. doi: 10.1155/2022/2021066 (PMC9012611; doi:10.1155/2022/2021066)
Supplement: Supplementary Materials — The 200 target genes interacting with the 10 bioactive components of PCRR, and the 2913 ALF-related target genes are provided in supplementary Tables S1 and S2, respectively. The 153 ALF-related PCRR target candidates are provided in supplementary Table S3. The results of the GO and KEGG pathway enrichment analyses are provided in supplementary Tables S4 and S5, respectively. [file 2021066.f1.zip › 2021066.f2.pdf]

Supplementary file

Table S2. 2913 ALF-related target genes

No.	Gene	No.	Gene	No.	Gene	No.	Gene	No.	Gene	No.	Gene	No.	Gene
1	TP53	56	BRCA2	111	ABCB1	166	FASLG	221	IL17A	276	MME	331	NFKB1
2	TERT	57	TMEM67	112	FAS	167	SH3GL1	222	CP	277	FAH	332	MIR22
3	JAK2	58	NSD1	113	NPPA	168	ERCC6	223	FOS	278	FANCM	333	CD46
4	RTEL1	59	CBFB	114	LARS1	169	ATM	224	XIAP	279	MKS1	334	CCL5
5	FLT3	60	NPPB	115	EGF	170	APC	225	TSC2	280	IL3	335	MET
6	TTN	61	DDX41	116	HNF4A	171	ATP7B	226	CSF2	281	NOS2	336	NOBOX
7	IL6	62	PIK3CA	117	SERPINA1	172	HADHB	227	TLR2	282	CST3	337	SRP72
8	RTEL1-TNFRSF6B	63	MIR122	118	BAX	173	POLG2	228	FMR1	283	U2AF1	338	NPHP1
9	LIPC	64	MYC	119	MPO	174	MIR320A	229	IL2RA	284	MT-ND1	339	GAREM2
10	TNF	65	HADHA	120	MEFV	175	HLA-DQB1	230	APOB	285	SETD2	340	SST
11	CEBPA	66	PTEN	121	GNB1	176	MAT1A	231	ADIPOQ	286	BCL2	341	FBN1
12	RUNX1	67	CC2D2A	122	IFNL3	177	MPL	232	PLAT	287	PTGS2	342	CALR
13	NPM1	68	CDKN2A	123	CHEK2	178	ABCB11	233	VWF	288	JUN	343	RPL5
14	TRMU	69	IDH1	124	MMP9	179	SLC25A13	234	CD34	289	SMPD1	344	HIF1A
15	IFNG	70	PARN	125	MIR210	180	MAPK1	235	IFIH1	290	BCS1L	345	MAPK8
16	KRAS	71	PAX5	126	MIR203A	181	IFNA2	236	MIR152	291	PIK3C2A	346	LDLR
17	ALB	72	SLC17A5	127	F3	182	LPL	237	NR1H4	292	RYR1	347	HLA-DQA1
18	RANBP2	73	PKD1	128	PTPRC	183	CFTR	238	MIRLET7C	293	SF3B1	348	ASS1
19	IL10	74	PML	129	RBCK1	184	CFH	239	HBB	294	SOD1	349	CD79A
20	INSL6	75	IKZF1	130	THPO	185	CXCL10	240	TSC1	295	CCND1	350	MIR16-1
21	STAT3	76	TERC	131	HLA-C	186	HAVCR2	241	MYH11	296	COL4A5	351	ADRB2
22	KIT	77	FOXP3	132	PTPN11	187	LCN2	242	GALT	297	CYP2D6	352	MILR1
23	HMBS	78	ERCC6L2	133	NOS3	188	TF	243	NRAS	298	CHAT	353	RUNX1T1
24	ETV6	79	SP140	134	STAT1	189	MIR15A	244	PBX1	299	PMS2	354	MT-CYB
25	RINT1	80	POLG	135	CCL2	190	CEP290	245	TCF3	300	TINF2	355	FOXL2
26	F2	81	IDH2	136	IL18	191	SERPINE1	246	MDM2	301	CXCL12	356	IL6R
27	GPT	82	VEGFA	137	LOC110806263	192	HGF	247	LEP	302	IL1A	357	MIR18A
28	ACE	83	ACTB	138	CTLA4	193	AKT1	248	KRT8	303	MYH7	358	EP300
29	NPHP3	84	HLA-B	139	HP	194	HNF1B	249	GSTM1	304	WDR19	359	HADH
30	KMT2A	85	THBD	140	IL2	195	EGFR	250	MIR27A	305	PDGFRA	360	CYP2E1
31	CRP	86	CTNNB1	141	PPARG	196	SMAD4	251	FGFR1	306	CSF3R	361	TNFRSF1A
32	HFE	87	APOE	142	CASP8	197	AGTR1	252	TIMP1	307	HJV	362	MAP2K1
33	CPT2	88	PKHD1	143	PKD2	198	SCARB2	253	PRKAR1A	308	CD81	363	CPT1A
34	NBAS	89	HNF1A	144	BCR	199	MBL2	254	MYB	309	EIF2AK3	364	MT-ND4
35	GBE1	90	F5	145	MIR204	200	SBDS	255	CDKN1A	310	MT-ATP6	365	NR5A2
36	LMNA	91	CCR5	146	FADD	201	CASP3	256	PMM2	311	NUP98	366	SEPTIN9
37	DNAJC21	92	RARA	147	SERPINC1	202	IGF1	257	UROD	312	MTM1	367	MIRLET7D
38	DNMT3A	93	INPP5E	148	IL18BP	203	KRT18	258	CCR6	313	ANXA5	368	NOTCH2
39	GATA2	94	MIR21	149	NEK8	204	CD4	259	SERPINA3	314	PPOX	369	GAA
40	NBN	95	TLR4	150	HAVCR1	205	C3	260	MIR221	315	MMP2	370	ITGB3
41	MIR155	96	AFP	151	ALDH2	206	MIR34A	261	TNNT2	316	VHL	371	CDKN2B
42	HLA-DRB1	97	RPGRIP1L	152	MIR17	207	GGT1	262	JAK3	317	FECH	372	GATA4
43	IL1B	98	REN	153	CRLF2	208	CPOX	263	MPV17	318	JAG1	373	MMP1
44	INS	99	TLR3	154	ELANE	209	POF1B	264	TGM6	319	SURF1	374	SFTPC
45	SP110	100	WT1	155	JAK1	210	ICAM1	265	CFI	320	IGF2R	375	G6PD
46	TET2	101	STAT5B	156	LPP	211	CD40LG	266	FN1	321	B2M	376	DGKE
47	NUP214	102	STAR	157	ZBTB16	212	GATA3	267	TICAM1	322	BRCA1	377	CDKN3
48	ABL1	103	LPIN1	158	CREBBP	213	MECOM	268	APOA1	323	MTOR	378	DYNC2H1
49	MIR126	104	IRF3	159	NOTCH1	214	CAV1	269	SCYL1	324	ASXL1	379	MIR23A
50	CXCL8	105	IL4	160	TBK1	215	CYP3A4	270	AGT	325	IL7R	380	BRAF
51	GATA1	106	MTHFR	161	TAL1	216	ABCB4	271	IGHMBP2	326	MIR222	381	ANPEP
52	ALAD	107	TTR	162	EDN1	217	ENG	272	FIP1L1	327	EIF2AK2	382	CYCS
53	TGFB1	108	CSF3	163	HRAS	218	HMOX1	273	PRF1	328	ADA	383	FLT1
54	IFNA1	109	EPO	164	HAMP	219	MT-CO3	274	ADAMTS13	329	FANCA	384	CLCN6
55	MIR223	110	HLA-A	165	IL1RN	220	MIR146A	275	DDX3X	330	MIR29A	385	SLC22A12

Table S2. 2913 ALF-related target genes (contrinued)

No.	Gene	No.	Gene	No.	Gene	No.	Gene	No.	Gene	No.	Gene	No.	Gene
386	GBA	441	SCARB1	496	LIPA	551	SCN5A	606	IRF7	661	POLR1C	716	PTHLH
387	SLC22A5	442	MIR20A	497	STAT5A	552	NFKBIA	607	CCL11	662	NOTCH3	717	PCK1
388	INSR	443	ABCC2	498	CYP3A5	553	CDH1	608	HSD3B7	663	RPS19	718	PECAM1
389	SLC10A1	444	TFRC	499	IRF2BP2	554	TNFSF10	609	TCIRG1	664	NDUFS4	719	ADH5
390	SARS2	445	MYH6	500	AXIN1	555	AGXT	610	MYD88	665	AHI1	720	STAT2
391	IFT140	446	NCAM1	501	RPS26	556	TSPO	611	TEK	666	ABCD1	721	ALAS2
392	ESR1	447	ERCC1	502	SRC	557	MAPK14	612	MIR199A1	667	MST1	722	CYP19A1
393	IFNB1	448	MIR23B	503	LGALS3	558	MVK	613	NLRP3	668	TGFB3	723	EFL1
394	MIR127	449	MIR106B	504	DNAH8	559	HOTAIR	614	LOC107303340	669	PTCH1	724	CXCR3
395	CYP1A1	450	MX1	505	OTC	560	FGA	615	MMACHC	670	HLA-DPB1	725	CYP7A1
396	GSTP1	451	ABCC8	506	CD14	561	MT-ND2	616	LRRC56	671	SMAD3	726	PRL
397	BTK	452	NR1H2	507	SLC25A15	562	ERBB3	617	SDHA	672	CBS	727	PRTN3
398	MIR181A1	453	TJP2	508	VCAM1	563	ERCC2	618	ACADVL	673	COL4A3	728	CCL3
399	DMD	454	PTH1R	509	IL13	564	TCF4	619	SLC2A2	674	LMBRD1	729	SKIV2L
400	MT-TL1	455	LOC110806306	510	LEPR	565	CDKN1C	620	PPARGC1A	675	BMP2	730	SPINK1
401	IKBKG	456	CLCN5	511	CBL	566	RETN	621	NOD2	676	LOX	731	SYK
402	ACAD9	457	PTX3	512	TMEM216	567	SET	622	CDK1	677	AVP	732	ACTC1
403	MIR181A2	458	DSP	513	MIR25	568	ACADM	623	TTC21B	678	ERCC4	733	GAPDH
404	CD19	459	FANCC	514	POMC	569	MRTFA	624	NKX2-5	679	MMP3	734	IL12A
405	DDX58	460	NABP1	515	PON1	570	IFNAR2	625	IL15	680	CACNA1S	735	CDKN2B-AS1
406	ABCC1	461	ALMS1	516	BDNF	571	MUC1	626	LIG4	681	BAAT	736	HLA-G
407	BCOR	462	MT-ND6	517	NFE2L2	572	FABP1	627	IL2RB	682	EDNRB	737	SQSTM1
408	TRAF3	463	ELN	518	CD40	573	MT-CO1	628	CFHR1	683	NQO1	738	MSH2
409	TPMT	464	ITGAM	519	IL1R1	574	DES	629	ACADS	684	HMGCR	739	TMEM237
410	CCN2	465	ABCG2	520	IFT172	575	BIRC5	630	MAVS	685	DNMT3B	740	FCGR2A
411	SAMD9L	466	NPC1	521	SAA1	576	NF1	631	MCL1	686	CIITA	741	ERBB2
412	PLG	467	KDR	522	NR3C2	577	TRB	632	CYP1A2	687	EDN3	742	MYSM1
413	RSF1	468	INVS	523	RAD51	578	COL4A4	633	GLIS2	688	XRCC4	743	GCK
414	ADM	469	CREB1	524	CXCR4	579	GLA	634	PRKCD	689	BCL10	744	MEG3
415	SPP1	470	ACVRL1	525	TFR2	580	EZH2	635	PNPLA3	690	NPHP3-AS1	745	IL6ST
416	PPARA	471	OCLN	526	RBP4	581	MIR181C	636	CD274	691	KCNJ11	746	AQP2
417	BCL2L1	472	XRCC2	527	SELE	582	ENPP1	637	AIRE	692	MIR93	747	LPA
418	MTTP	473	RAF1	528	HMGB1	583	LBP	638	GRB2	693	LCK	748	SCN1A
419	CALCA	474	FANCI	529	PEX6	584	PMP22	639	CD28	694	OFD1	749	GHR
420	MB	475	ATP8B1	530	BMP15	585	ADCY10	640	CSF1	695	CYP2B6	750	B9D1
421	KITLG	476	CYP11A1	531	RET	586	NPHP4	641	SIRT1	696	ZCCHC8	751	SOD2
422	SELP	477	HFE-AS1	532	IGF2	587	CD8A	642	GLUL	697	EDAR	752	UNC93B1
423	BSCL2	478	AGPAT2	533	MIF	588	CHUK	643	SYCE1	698	APRT	753	IGF1R
424	SEPSECS	479	MT-ND3	534	CASR	589	ACE2	644	SLCO1B1	699	ALG9	754	IL12RB1
425	CFB	480	CDKN1B	535	AKR1D1	590	TFAM	645	DNMT1	700	IKBKB	755	CAVIN1
426	MIR19A	481	NR3C1	536	ADRB1	591	TMEM231	646	SRSF2	701	SRP54	756	CETP
427	ABCA3	482	DZIP1L	537	ASGR1	592	CYP2C9	647	TGFBR1	702	UGT1A1	757	AARS2
428	MT-ND5	483	ITGA2B	538	CD44	593	PTPN22	648	NDUFAF2	703	G6PC1	758	RPS14
429	BMP6	484	DGUOK	539	SLC40A1	594	RUNX2	649	SOCS3	704	NDUFS2	759	TLR9
430	LTA	485	CLDN1	540	RB1	595	MIR125A	650	TWNK	705	GDF9	760	ATRX
431	VDR	486	IL5	541	RARB	596	MIR143	651	IFT122	706	APOC2	761	TGFBR2
432	LAMTOR5	487	KNG1	542	CLEC4M	597	KRT19	652	CDK4	707	CXCR1	762	ALOX5
433	DKC1	488	MAPK3	543	MIR196A2	598	GH1	653	WDR35	708	TCTN2	763	CDK2
434	FGF2	489	H2AC18	544	IL7	599	CAT	654	IQCB1	709	ADAR	764	SFTPA2
435	CD33	490	SOHLH1	545	MIR181B1	600	MSH5	655	STIL	710	PCSK9	765	ADORA2A
436	UMOD	491	CYP2C19	546	CD36	601	PLAUR	656	HPRT1	711	SON	766	LOC101928371
437	XDH	492	SLC2A9	547	PARP1	602	GC	657	WRAP53	712	ANKRD26	767	GANAB
438	ASGR2	493	SOCS1	548	IFNL4	603	CASP9	658	ACTA1	713	CEP19	768	STK11
439	MIR195	494	RAG1	549	CEBPB	604	CFHR5	659	GNAS	714	BGLAP	769	IFNGR1
440	PDCD1	495	SLC4A1	550	ANGPT2	605	GDNF	660	IGFBP3	715	NAGLU	770	GLIS3

Table S2. 2913 ALF-related target genes (contrinued)

No.	Gene	No.	Gene	No.	Gene	No.	Gene	No.	Gene	No.	Gene	No.	Gene
771	MIR499A	826	SFTPD	881	RXRA	936	HDAC9	991	RPS24	1046	CTSB	1101	VIPAS39
772	GFI1	827	HSPA4	882	CD247	937	ECE1	992	NR1I2	1047	TRPV4	1102	MDM4
773	SLC6A4	828	MIR199B	883	ERCC8	938	ABCC3	993	SCO2	1048	CXCR2	1103	CASP7
774	LEF1	829	CYC1	884	TYK2	939	DPAGT1	994	LIF	1049	TGM1	1104	FHIT
775	RUNX3	830	ARG1	885	RRM2B	940	PEX1	995	ALAS1	1050	FANCL	1105	PIK3C2G
776	SH2D1A	831	HDAC1	886	MIR378A	941	FKRP	996	MLLT1	1051	COX5A	1106	HNRNPUL2-BSCL2
777	B9D2	832	FANCG	887	CD86	942	PAH	997	CLDN6	1052	NPHS2	1107	PSMB9
778	GSTT1	833	NDUFV1	888	GYPA	943	FLNC	998	ANGPT1	1053	IL11	1108	SLC7A7
779	TTC37	834	COQ2	889	NPY	944	MIR9-1	999	IRF8	1054	SREBF1	1109	NDUFAF6
780	SELL	835	SDCCAG8	890	MCCC1	945	AR	1000	LRP5	1055	CD38	1110	SMC3
781	VCL	836	SLPI	891	ABCC6	946	F8	1001	MIR140	1056	IFT43	1111	TUG1
782	MARS1	837	TNFRSF1E	892	CCNA2	947	SFTPA1	1002	FOXO1	1057	LYN	1112	RPS7
783	SLC2A1	838	AMACR	893	TIMP2	948	HSP90AA1	1003	CYP7B1	1058	AGK	1113	SUZ12
784	FABP3	839	ABCB7	894	DIPK1A	949	BLM	1004	GSR	1059	MMP8	1114	RPS17
785	AIP	840	XRCC1	895	BAG3	950	PNLIP	1005	NPHS1	1060	TREX1	1115	TRA
786	CYP17A1	841	DIAPH2	896	BMPR2	951	CHPT1	1006	PDE5A	1061	CHIT1	1116	SLCO1B3
787	ALPP	842	NFRSF13I	897	AVPR2	952	MIR224	1007	SELENON	1062	MIR324	1117	FARSB
788	PRSS1	843	CFHR3	898	DLD	953	LDHA	1008	TK2	1063	ALK	1118	IL21
789	NHP2	844	UROS	899	TSFM	954	F7	1009	ACOX1	1064	IGFBP2	1119	NTRK1
790	PIK3CG	845	CCDC85B	900	CSF1R	955	NDUFS7	1010	MEIS1	1065	MIR125B1	1120	TG
791	TCTN3	846	RPL11	901	TNFRSF8	956	NDUFA13	1011	GLI1	1066	GFER	1121	IL3RA
792	TGFB2	847	IRF5	902	C4A	957	PLAU	1012	TNFAIP3	1067	BSG	1122	AHCY
793	PSAP	848	CHGA	903	GPR35	958	MIR150	1013	KRT7	1068	IL4R	1123	HSPD1
794	MYBPC3	849	PTGS1	904	SCO1	959	PMFBP1	1014	SMN1	1069	TGIF1	1124	CPD
795	PROM1	850	NAGS	905	TGFA	960	GFAP	1015	AKT2	1070	ITCH	1125	MIR34C
796	HMGCL	851	MT-TK	906	OC1071335	961	LBR	1016	PNPLA2	1071	PTPA	1126	DDX3Y
797	CYP11B2	852	PIK3R1	907	NOS1	962	PALB2	1017	TPO	1072	GUSB	1127	NEAT1
798	IL10RB	853	MIR191	908	ITGAL	963	FLNA	1018	GDF15	1073	FANCF	1128	KCNQ1
799	NPPC	854	GHRL	909	MPI	964	RBM15	1019	SERPINF2	1074	ICOSLG	1129	BRIP1
800	PF4	855	EDNRA	910	RPS6KB1	965	CASP1	1020	RAG2	1075	MIRLET7E	1130	PLA2G6
801	MECP2	856	ACHE	911	HOXA9	966	COL1A1	1021	BCL2L11	1076	KMT2D	1131	IL22
802	RLBP1	857	BCL6	912	C4B	967	PPIG	1022	CTCF	1077	DPP4	1132	ZAP70
803	KDM4C	858	TYMP	913	TLR5	968	HLA-DPA1	1023	IGH	1078	ABCD3	1133	BBS1
804	GLS	859	MIR200B	914	S100B	969	AURKC	1024	THY1	1079	PEX13	1134	AHSG
805	SMARCA4	860	FOXP1	915	PI4KA	970	CD7	1025	IFI27	1080	NKX2-1	1135	POU5F1
806	TYMS	861	KLRK1	916	SPI1	971	NFRSF10I	1026	PVT1	1081	PIGA	1136	SLC19A1
807	APOBEC3G	862	CASP10	917	AGER	972	KL	1027	MBP	1082	H19	1137	APBB1
808	NOP10	863	NFRSF11I	918	MIR130A	973	VPS33B	1028	RSAD2	1083	MIR30D	1138	HSPG2
809	RYR2	864	MALAT1	919	HSPA1A	974	MTR	1029	RELA	1084	GALE	1139	TAC1
810	APOH	865	IFNAR1	920	FOXO3	975	EPOR	1030	MIR483	1085	CD163	1140	CCL4
811	MVP	866	HAX1	921	IRS1	976	ADA2	1031	ALDOB	1086	CLCNKB	1141	HSPA5
812	FOXRED1	867	OC1066279	922	DHFR	977	IGFBP1	1032	CXCL9	1087	MYH9	1142	FCGR3A
813	CD209	868	TLR7	923	GZMB	978	SDHB	1033	MLXIPL	1088	AXL	1143	MIR124-1
814	KIR3DL1	869	LCAT	924	HPX	979	PYGM	1034	NR1I3	1089	ISG15	1144	HMGA2
815	DNAJC19	870	FGF23	925	LMO2	980	DCK	1035	MT-TE	1090	EPHX1	1145	DBH
816	FBXW7	871	PSMB8	926	ANKS6	981	PHKG2	1036	THBS1	1091	CLDN9	1146	BLNK
817	DEK	872	DNAJB11	927	PSMA7	982	MYCN	1037	TANGO2	1092	FANCD2	1147	SDHD
818	MIR142	873	PDGFRB	928	OC1060990	983	GCG	1038	PEX5	1093	IL12B	1148	NR0B1
819	RNASE3	874	ITGB1	929	FH	984	SCGB1A1	1039	MRPS7	1094	PLK1	1149	PTMA
820	PRDM16	875	CD80	930	SHH	985	TRIP4	1040	CCK	1095	LAMP2	1150	CXCL1
821	SLC25A20	876	IRF1	931	OC1100063	986	TNFSF11	1041	CORIN	1096	GATA6	1151	ABCC4
822	VIM	877	TOP1	932	SH2B3	987	CD55	1042	PTH	1097	SLC37A4	1152	SLC34A1
823	APOC3	878	CDK6	933	TAFAZZIN	988	ANXA2	1043	SMAD2	1098	MIR30A	1153	C5
824	SUN5	879	CSF2RA	934	STAT4	989	TCL1A	1044	GJA1	1099	PRODH	1154	PRAME
825	ETFDH	880	MIR24-2	935	CSPP1	990	MUC5B	1045	MEN1	1100	MT-TW	1155	SLC9A3

Table S2. 2913 ALF-related target genes (contrinued)

No.	Gene	No.	Gene	No.	Gene	No.	Gene	No.	Gene	No.	Gene	No.	Gene
1156	ITGB2	1211	ASL	1266	GSN	1321	KIR2DL3	1376	CCL17	1431	KMT2C	1486	BTNL2
1157	RAD51C	1212	CD5	1267	APOL1	1322	CHEK1	1377	RPN1	1432	ITPA	1487	SEC63
1158	NCR1	1213	SERPINA7	1268	GP1BA	1323	SUFU	1378	NFU1	1433	RPS28	1488	SHBG
1159	ETFB	1214	MIR182	1269	KLRC1	1324	SUMO1	1379	PCCB	1434	RPL31	1489	CYBB
1160	AUTS2	1215	GFM1	1270	LMF1	1325	CLEC12A	1380	UTS2	1435	TREM1	1490	CASP2
1161	ALPL	1216	ALG8	1271	XBP1	1326	MS4A1	1381	FCGR1A	1436	ATP7A	1491	APEX1
1162	PICK1	1217	GOLM1	1272	RPL26	1327	MTAP	1382	GSK3B	1437	SLC10A2	1492	GLE1
1163	PEX19	1218	HBG2	1273	TAP2	1328	CR2	1383	VCP	1438	PSEN1	1493	SLC35A2
1164	TBX5	1219	BCKDHB	1274	RNF31	1329	UCA1	1384	RPL15	1439	NCOA2	1494	PRKN
1165	BMI1	1220	CLCNKA	1275	GRHPR	1330	FANCB	1385	AURKB	1440	TCN2	1495	OSTM1
1166	FGFR3	1221	PRKCA	1276	CPS1	1331	ANKRD1	1386	FASN	1441	SLC6A8	1496	NTS
1167	IFT80	1222	COMT	1277	NEU1	1332	RPS29	1387	NAMPT	1442	TOP2A	1497	MC2R
1168	PAX2	1223	SP1	1278	CYBA	1333	PCK2	1388	CDA	1443	SMARCB1	1498	TIMP3
1169	ETFA	1224	TALDO1	1279	MIR196A1	1334	NDUFAF1	1389	HDAC2	1444	GSTA1	1499	FOXA2
1170	SKP2	1225	NME1	1280	RFX5	1335	PTPN6	1390	RPL18	1445	SLC12A3	1500	CNOT3
1171	CRH	1226	MIR128-2	1281	MIR148A	1336	TRMT10C	1391	TH	1446	FBP1	1501	TMEM126B
1172	PPBP	1227	MIR328	1282	SRP19	1337	RHOA	1392	PEX11B	1447	PRKCSH	1502	CRYAB
1173	RFXANK	1228	CCND2	1283	NCL	1338	NSMCE2	1393	RPL35A	1448	SLC1A2	1503	CD2
1174	RPS10	1229	CFHR4	1284	AMPD1	1339	SAMHD1	1394	HSD17B4	1449	LRP6	1504	CD9
1175	MT-TV	1230	PRKAG2	1285	TTC7A	1340	MIR214	1395	VKORC1	1450	MIR192	1505	MAOA
1176	FANCE	1231	AGTR2	1286	FGB	1341	CCR2	1396	DLAT	1451	MPZ	1506	KLRD1
1177	IL1RAPL2	1232	NPC2	1287	MT-CO2	1342	AMBP	1397	KIR2DL1	1452	MRPL44	1507	CD96
1178	BCHE	1233	TEX14	1288	GATM	1343	MIR15B	1398	LOC102723566	1453	NSUN2	1508	CRKL
1179	STN1	1234	BICC1	1289	ASNS	1344	PGF	1399	FOXE3	1454	BBS4	1509	MYO5B
1180	KDM6A	1235	MIR424	1290	LIPE	1345	SF3B2	1400	NDUFS1	1455	GNMT	1510	DIABLO
1181	PYGL	1236	TP73	1291	RFXAP	1346	GALK1	1401	ISG20	1456	C1S	1511	IL18R1
1182	MIR331	1237	OPRM1	1292	IL33	1347	ASAH1	1402	NR0B2	1457	FTL	1512	GLI3
1183	LTF	1238	CX3CR1	1293	EVC	1348	ABCA1	1403	MATR3	1458	SIGLEC5	1513	FTO
1184	CCDC115	1239	FSHR	1294	PDX1	1349	PIK3CD	1404	EGR1	1459	LAMA2	1514	PNP
1185	IFIT1	1240	DCTN1	1295	NEK1	1350	HCRT	1405	SLC25A4	1460	SOS1	1515	CYP2A6
1186	STEAP3	1241	MIRLET7E	1296	PEX14	1351	NCR3	1406	P4HB	1461	TNFSF15	1516	DNASE1
1187	BMP7	1242	KLF4	1297	MTFMT	1352	DNAH2	1407	PDE4D	1462	TNFRSF9	1517	AZU1
1188	FASTKD2	1243	MIR151A	1298	SMN2	1353	CACNA1C	1408	CD3D	1463	LARS2	1518	HNRNPH1
1189	OTULIN	1244	LDB3	1299	PLA2G7	1354	CDH2	1409	TRIM37	1464	RPS15A	1519	IRF4
1190	NCOR1	1245	KIF7	1300	CAPN3	1355	VIP	1410	DSG2	1465	ERCC5	1520	TNFRSF13C
1191	NCOR2	1246	ITGAX	1301	SLX4	1356	NHLRC1	1411	GPIHBP1	1466	GNRH1	1521	NARS2
1192	LACTB	1247	PEX16	1302	GAST	1357	SCT	1412	MUSK	1467	TAT	1522	SAA2
1193	CYP27B1	1248	GDF2	1303	CLDN16	1358	ARID1B	1413	CTC1	1468	IDO1	1523	CD27
1194	EVC2	1249	CEACAM5	1304	TRD	1359	CTSD	1414	GNB3	1469	COG8	1524	CS
1195	PEX3	1250	SAA4	1305	ABCG5	1360	MEF2A	1415	IRF9	1470	ATRIP	1525	PGM1
1196	MAP2K2	1251	ITGA4	1306	MIR149	1361	FCGR3B	1416	DDOST	1471	AGL	1526	ATP11A
1197	SOX9	1252	F13A1	1307	COA8	1362	UCP2	1417	MRC1	1472	DLL4	1527	FIG4
1198	MIR145	1253	MKKS	1308	COL3A1	1363	TMPO	1418	IARS2	1473	TRG	1528	HM13
1199	PEX2	1254	PEX10	1309	ENO2	1364	MIR451A	1419	MMAA	1474	CAV3	1529	SDC1
1200	FHL1	1255	BRD4	1310	CFHR2	1365	CD22	1420	GOPC	1475	HDAC6	1530	PSMB4
1201	HSPB1	1256	EIF2S1	1311	MIR326	1366	SULT1A3	1421	PLA2G2A	1476	MMUT	1531	ATP5F1A
1202	CTSG	1257	MUC5AC	1312	SLC12A1	1367	FAM13A	1422	MIR144	1477	LUC7L2	1532	SPAG17
1203	MYL3	1258	CFLAR	1313	CR1	1368	CFAP92	1423	KIAA0586	1478	GPS2	1533	PHEX
1204	APAF1	1259	CHKA	1314	ZMPSTE24	1369	MIR130B	1424	SRF	1479	MIR10A	1534	MMP7
1205	RPS27	1260	SGSH	1315	F10	1370	FLI1	1425	CLCN7	1480	MGMT	1535	CHD7
1206	ARID5B	1261	DDB1	1316	CDKN2C	1371	TRAF3IP1	1426	DYNC2I1	1481	IL21R	1536	MIR29B1
1207	SCNN1A	1262	PCNA	1317	EPCAM	1372	JUP	1427	APLN	1482	S100A9	1537	AFDN
1208	EIF2AK1	1263	VAV1	1318	SERAC1	1373	RAC1	1428	FLNB	1483	AMH	1538	PHF6
1209	LEPQTL1	1264	ICOS	1319	RAD21	1374	MIR423	1429	CEBPE	1484	UQCRFS1	1539	NDUFB11
1210	SRPRA	1265	FGL2	1320	WRN	1375	TUSC7	1430	SOX2	1485	ARL13B	1540	TSPAN7

Table S2. 2913 ALF-related target genes (contrinued)

No.	Gene	No.	Gene	No.	Gene	No.	Gene	No.	Gene	No.	Gene	No.	Gene
1541	BPI	1596	ERBB4	1651	KCNK3	1706	ACTA2	1761	NNT	1816	H4C1	1871	TNFSF12
1542	DPYD	1597	MIR30C1	1652	LZTFL1	1707	FTH1	1762	PRKG1	1817	CPA1	1872	SPTAN1
1543	GYS1	1598	HNRNPA1	1653	TBC1D20	1708	IGHE	1763	HPD	1818	GLUD1	1873	MIR24-1
1544	TCF7L2	1599	CREB3L3	1654	ITIH4	1709	APOA5	1764	COG2	1819	MAOB	1874	MICA
1545	EIF4E	1600	SOX4	1655	HELLS	1710	CCAT1	1765	TNFSF13B	1820	EPX	1875	IL23A
1546	NDUFAF8	1601	SNCA	1656	NR1H3	1711	RMND1	1766	SLC6A3	1821	EHHADH	1876	PROC
1547	ACD	1602	NAT2	1657	MIR338	1712	ACTG1	1767	S100A8	1822	RMND5B	1877	COG7
1548	GPD1	1603	CYP21A2	1658	HTR1A	1713	FUT4	1768	TXN	1823	MT-TF	1878	NGF
1549	HSPA8	1604	LOC105371049	1659	CHI3L1	1714	CEACAM3	1769	CCR7	1824	VEGFC	1879	RPL27
1550	CD59	1605	DICER1	1660	USB1	1715	PITX2	1770	KCNN4	1825	CITED2	1880	AP1B1
1551	HHEX	1606	P2RY12	1661	MGP	1716	MYL2	1771	PPIA	1826	TERF1	1881	G6PC3
1552	OCRL	1607	SOX10	1662	HLA-DRA	1717	CYP24A1	1772	CYP27A1	1827	MMP12	1882	RBP1
1553	EPRS1	1608	MTRR	1663	NDUFA6	1718	CNR1	1773	TXNRD2	1828	HVBS7	1883	UGT1A7
1554	APP	1609	PTPN2	1664	HOXA10	1719	EPAS1	1774	CLU	1829	ERG	1884	SYP
1555	CYB5R3	1610	ATP2A2	1665	LIFR	1720	PNPLA6	1775	C1QTNF3-AMACR	1830	FURIN	1885	MIR128-1
1556	RASA1	1611	LRP2	1666	MKI67	1721	IFT52	1776	YAP1	1831	OPTN	1886	CARS1
1557	SHC1	1612	REG3A	1667	CEP83	1722	ALG2	1777	ATG5	1832	NT5C2	1887	HNRNPK
1558	HBB-LCR	1613	PORCN	1668	IL36RN	1723	ITGA5	1778	HTR2A	1833	XIST	1888	TPI1
1559	KCNJ5	1614	ADAM17	1669	NCF1	1724	ABCC5	1779	ID4	1834	CRAT	1889	CA2
1560	FCGR2B	1615	ALOX5AP	1670	GFI1B	1725	HSD11B2	1780	ITGA2	1835	TMEM70	1890	GGH
1561	EIF4EBP1	1616	OLR1	1671	TAP1	1726	GALC	1781	ORM1	1836	AGO2	1891	AARS1
1562	TYR	1617	CPLANE1	1672	SUCLG1	1727	PTK2	1782	BUB1	1837	FGF7	1892	CDX2
1563	YARS1	1618	KLK3	1673	TRPM3	1728	ENO1	1783	ACP1	1838	INHA	1893	PRDX1
1564	STMN1	1619	WNT5A	1674	MLH1	1729	MAPT	1784	S100A12	1839	NCOA3	1894	DKK1
1565	TSR2	1620	AOC3	1675	DYRK1A	1730	PDHA1	1785	ACP5	1840	CDK9	1895	ATP6AP2
1566	BBS9	1621	GPX3	1676	TGM2	1731	RPS20	1786	GRK2	1841	F12	1896	SLC35C1
1567	TP63	1622	PCCA	1677	TNFRSF10A	1732	FMO3	1787	SPINK5	1842	MCCC2	1897	POLR3A
1568	LOC10050554	1623	HOGA1	1678	WNT4	1733	DLL1	1788	NDUFV2	1843	IL9	1898	RASSF1
1569	DDIT3	1624	E2F1	1679	RASGRP1	1734	CUBN	1789	BOLA3	1844	GHRHR	1899	SLC3A1
1570	PKHD1L1	1625	PMPCA	1680	ABCG8	1735	NFKB2	1790	AQP1	1845	PLIN1	1900	CDH23
1571	KHDRBS1	1626	OXT	1681	PRKD1	1736	POLR2A	1791	IL2RG	1846	IRAK4	1901	LTBP4
1572	DAXX	1627	PRNP	1682	DDC	1737	BAK1	1792	CXCR5	1847	SKI	1902	BIRC2
1573	NUBPL	1628	ACSF3	1683	MIR1225	1738	FOXM1	1793	ABHD5	1848	ADK	1903	FES
1574	TCAP	1629	SMAD7	1684	UQCRB	1739	CYP1B1	1794	PRKCB	1849	UBA1	1904	PLCE1
1575	TXNDC15	1630	HRH2	1685	PNKD	1740	IFITM3	1795	DPP9	1850	EIF2B3	1905	MT-TS1
1576	FBXL4	1631	MIR212	1686	SPRTN	1741	COX10	1796	COL1A2	1851	ARID1A	1906	FGF21
1577	NDUFAF5	1632	MIR335	1687	KDM1A	1742	HOXA7	1797	NAXE	1852	DRD2	1907	PSC
1578	SLC11A2	1633	AOPEP	1688	ATG7	1743	POSTN	1798	NDUFS8	1853	RPL35	1908	TKT
1579	NUP62	1634	GPC3	1689	APOA2	1744	SPARC	1799	PC	1854	DDB2	1909	SEMA3C
1580	XPO1	1635	CIDEC	1690	DACT1	1745	CCR9	1800	FBLN5	1855	HVBS8	1910	CTH
1581	PAPPA	1636	IL23R	1691	POR	1746	SLC3A2	1801	MIR374A	1856	MUS81	1911	PKM
1582	MRPL12	1637	PON2	1692	INF2	1747	SUCLA2	1802	HBA1	1857	PDGFB	1912	NANOG
1583	FLT3LG	1638	TNFRSF11A	1693	MMP14	1748	MIR30E	1803	ACSL4	1858	CCND3	1913	INPP5D
1584	IL1RL1	1639	MIR193A	1694	H4-16	1749	RTN4	1804	GCDH	1859	SOD3	1914	MT-TI
1585	COG4	1640	USP18	1695	IREB2	1750	LTC4S	1805	POU2AF1	1860	LPIN2	1915	FGG
1586	VAPB	1641	RNASEH2B	1696	MIR31	1751	CXCR6	1806	RIPK1	1861	TAF15	1916	ATF4
1587	MIR27B	1642	TBX1	1697	ZEB2	1752	WAS	1807	TRAPPC11	1862	BMPR1A	1917	CLDN19
1588	RARS1	1643	VIPR1	1698	CD69	1753	NDUFB9	1808	DGAT1	1863	RNASEH2C	1918	PRSS2
1589	MRPS34	1644	IARS1	1699	DARS2	1754	NDUFS3	1809	IRS2	1864	BECN1	1919	TBX21
1590	NEK9	1645	TRIM22	1700	RPGRIP1	1755	MLF1	1810	DEPDC5	1865	CARD14	1920	ATP1A3
1591	KCNH2	1646	DYNC2LI1	1701	CXCL2	1756	BMP4	1811	AQP4	1866	ASCL1	1921	CD68
1592	NAIP	1647	CTNS	1702	WT1-AS	1757	ADORA1	1812	EIF2AK4	1867	MT-TT	1922	IGHV4-38-2
1593	SIN3A	1648	GNAQ	1703	GLI2	1758	CD24	1813	SI	1868	SERPING1	1923	VTN
1594	NDUFAF3	1649	YARS2	1704	SLC30A10	1759	PGR	1814	BBS7	1869	LRBA	1924	FUS
1595	KHK	1650	LYRM7	1705	LGALS9	1760	NDUFA1	1815	BAD	1870	CYP26A1	1925	GAS6

Table S2. 2913 ALF-related target genes (contrinued)

No.	Gene	No.	Gene	No.	Gene	No.	Gene	No.	Gene	No.	Gene	No.	Gene
1926	SLAMF1	1981	SSBP1	2036	PIK3CB	2091	ELP1	2146	FCER2	2201	AQP9	2256	DNTT
1927	MIR6511B1	1982	CD48	2037	CTF1	2092	ALG1	2147	BRD2	2202	THRA	2257	PLN
1928	METTL3	1983	SLC11A1	2038	COL2A1	2093	COG1	2148	SPCS1	2203	IOXA-AS	2258	UGT1A3
1929	HNRNPA2B1	1984	GAL	2039	KIF1B	2094	APC2	2149	MYOCD	2204	PLA2R1	2259	UGT1A4
1930	OSM	1985	HCFC1	2040	GUCY2C	2095	H3-2	2150	HTR3A	2205	LRRK2	2260	BNC1
1931	STING1	1986	XPNPEP3	2041	CD3E	2096	MIR615	2151	AFF4	2206	FCN2	2261	MCM8
1932	VPS33A	1987	FABP2	2042	ABCB6	2097	C5AR1	2152	RRM2	2207	TNFRSF4	2262	DPY19L2
1933	F9	1988	CDC25A	2043	DUSP1	2098	BRF1	2153	MIR133A1	2208	SSR4	2263	MYPN
1934	PCBP2	1989	UBE2L3	2044	CDH5	2099	CEP120	2154	HOXA5	2209	FOXP2	2264	DLC1
1935	NDUFAF4	1990	AKT3	2045	CEACAM	2100	NHLRC2	2155	HPSE	2210	COQ4	2265	UGT1A10
1936	MIR486-1	1991	RBPJ	2046	IL10RA	2101	PAX4	2156	BIRC3	2211	ATF6	2266	COX8A
1937	TNFRSF6B	1992	MCM4	2047	A2ML1	2102	ANGPTL4	2157	DOT1L	2212	RPS27A	2267	NGLY1
1938	SLC22A6	1993	STAT6	2048	KLF9-DT	2103	MADD	2158	GSTZ1	2213	VPREB1	2268	KCNE2
1939	MIR208B	1994	CCNC	2049	MLN	2104	SDHC	2159	FGF1	2214	PNPLA8	2269	MT-TN
1940	APOA4	1995	COL4A1	2050	FUT8	2105	F2R	2160	PSTPIP1	2215	TRIP-TREX	2270	MICOS13
1941	HDAC3	1996	NASEH2	2051	MIR590	2106	PODXL	2161	XRCC5	2216	UMPS	2271	KDM5A
1942	OPA1	1997	ID1	2052	SP100	2107	FNDC5	2162	MARCAL	2217	STOX1	2272	MYLK
1943	FPGS	1998	LMNB2	2053	DEFB4A	2108	SGCD	2163	SMARCA2	2218	BHLHE40	2273	ACOX2
1944	SMC1A	1999	MYRF	2054	DNM1L	2109	IRAK1	2164	CLIC2	2219	SLC22A2	2274	UGT2B7
1945	WFS1	2000	CDC6	2055	ANG	2110	TRIP13	2165	FER	2220	BCORL1	2275	GCKR
1946	SLC52A3	2001	SPART	2056	DLK1	2111	DMBT1	2166	HCK	2221	LOC100507346	2276	TMEM199
1947	KARS1	2002	PEX7	2057	MIR340	2112	CEP57	2167	KIF1C	2222	CLEC7A	2277	ONECUT1
1948	NCR2	2003	BDKRB2	2058	IDUA	2113	CPQ	2168	DHCR24	2223	ATP6AP1	2278	MSH6
1949	MIR141	2004	CCR1	2059	PIGL	2114	MAP3K7	2169	SERPINA	2224	PRKCE	2279	UNC13D
1950	TNFSF4	2005	TFAP2A	2060	ACKR1	2115	UGT1A8	2170	AKR1B1	2225	ATP6V1B	2280	CNQ1OT1
1951	SALL4	2006	PIEZO1	2061	GDF1	2116	TRPV1	2171	TSLP	2226	MIR10B	2281	PEMT
1952	COX4I1	2007	UBA7	2062	BST2	2117	IVNS1AB	2172	RAB27A	2227	SLC5A1	2282	ADIPOR1
1953	UQCRC2	2008	CACNA1A	2063	HARS1	2118	UCP1	2173	SLC39A8	2228	TTN-AS1	2283	STX11
1954	EARS2	2009	CD47	2064	WNT2B	2119	TRIM32	2174	CCR4	2229	SETBP1	2284	MOGS
1955	TET1	2010	IGFBP7	2065	BCL11A	2120	CCL20	2175	CRABP1	2230	MLLT10	2285	ADH1B
1956	ALDH1A1	2011	PANK2	2066	C11orf65	2121	AFF2	2176	FYN	2231	PICALM	2286	LMO1
1957	GRP	2012	TUBB	2067	PTK2B	2122	MAPK10	2177	GPX1	2232	NR5A1	2287	GLS2
1958	STXBP2	2013	SETX	2068	VANGL2	2123	TET3	2178	EMD	2233	CHIC2	2288	ALGAPA1
1959	NDUFB3	2014	CD226	2069	IVD	2124	SLC9A1	2179	CXCL5	2234	TNNI3	2289	GLB1
1960	PDE4A	2015	MMAB	2070	DAPK1	2125	TJP1	2180	GLRX5	2235	STAG3	2290	SLC26A1
1961	ERN1	2016	DTNA	2071	CELA2A	2126	ATXN2	2181	GRIN2B	2236	BRDT	2291	PARS2
1962	SSB	2017	CCNB1	2072	MIR99A	2127	H4C2	2182	CCR3	2237	NUMA1	2292	DNAH17
1963	CCL18	2018	TIMMDC1	2073	CDK8	2128	H4C3	2183	MUC16	2238	TBL1XR1	2293	BHMT
1964	SPIB	2019	LRPPRC	2074	IL17F	2129	UGT1A	2184	MAP3K5	2239	PFKL	2294	TMPRSS6
1965	MIRLET7A1	2020	ARG2	2075	ADD1	2130	USH2A	2185	CTBP1	2240	AFF1	2295	TKFC
1966	CHRND	2021	PTF1A	2076	CES1	2131	MIR100HC	2186	UCHL1	2241	SFTPB	2296	UGT1A5
1967	NOTCH4	2022	CARS2	2077	MIR125B2	2132	CTSL	2187	CD2AP	2242	BAALC	2297	STX1A
1968	TNPO3	2023	ATP4A	2078	SLC2A4	2133	BUB1B	2188	RAI1	2243	KAT6A	2298	COX6B1
1969	ATP6V0A2	2024	FGF8	2079	ALG11	2134	RAPSN	2189	SCN4A	2244	MCM9	2299	NDUFB8
1970	MYL1	2025	GOT1	2080	MDK	2135	RECQL4	2190	DAMTSL	2245	PPA2	2300	HSD17B10
1971	DCLRE1C	2026	CISH	2081	AICDA	2136	AHR	2191	UBR1	2246	SLC13A3	2301	UBE2T
1972	C2CD3	2027	TCOF1	2082	SLC22A4	2137	KLF6	2192	NOX4	2247	RAD54L	2302	ACACA
1973	DYNC2I2	2028	CAMK2D	2083	UCN	2138	FLVCR2	2193	CA5A	2248	TLX1	2303	PRKAB1
1974	TRAF6	2029	MOG	2084	ETS1	2139	SNAI1	2194	MDH2	2249	FRAXA	2304	HULC
1975	MASP2	2030	NDUFA11	2085	OXA1L	2140	CTRC	2195	UGT1A6	2250	TLX3	2305	TACO1
1976	NDUFS6	2031	ADIPOR2	2086	PKLR	2141	PAX6	2196	ADAMTS1	2251	PLCZ1	2306	PRPS1
1977	MT-ATP8	2032	C1QBP	2087	IFITM1	2142	UQCRQ	2197	COPA	2252	UGT1A9	2307	TPM1
1978	ATP12A	2033	DPM1	2088	CYP2C8	2143	AURKA	2198	FHL2	2253	BLVRA	2308	MSH4
1979	SUOX	2034	PSMB10	2089	AKR1A1	2144	FGFR2	2199	DOCK6	2254	CDH17	2309	HBA2
1980	TNFAIP2	2035	COX15	2090	HSD11B1	2145	FOXF1	2200	PTGIS	2255	PHKB	2310	ADH1C

Table S2. 2913 ALF-related target genes (contrinued)

No.	Gene	No.	Gene	No.	Gene	No.	Gene	No.	Gene	No.	Gene	No.	Gene
2311	GNE	2366	MIR200A	2421	SLC4A2	2476	RMRP	2531	CHGB	2586	LOC113633877	2641	SLC51A
2312	PSEN2	2367	MHRT	2422	NRG1	2477	PKD2L1	2532	BAZ1B	2587	SERPINB1	2642	XPA
2313	BSND	2368	NDUFB10	2423	CEL	2478	CLPP	2533	ARID2	2588	SLC25A19	2643	ALDH18A1
2314	PRKAA1	2369	NEB	2424	NANOS1	2479	ELAC2	2534	PLD3	2589	PRDX3	2644	SHMT1
2315	ACTN2	2370	FXN	2425	GRK5	2480	CYP3A7	2535	IGF2BP2	2590	FCHO1	2645	CSTB
2316	CASK	2371	MFN2	2426	HYDIN	2481	LDB1	2536	HBZ	2591	CLEC4G	2646	KRT14
2317	TMEM43	2372	CTSA	2427	LOC111674472	2482	SCP2	2537	PIBF1	2592	MAGT1	2647	STARD13
2318	FGF19	2373	SALL2	2428	PKP2	2483	CANX	2538	TRPC6	2593	MIR205	2648	NAPB
2319	PKD1L1	2374	GOT2	2429	ABCC9	2484	JMJD1C	2539	EIF2B2	2594	PMAIP1	2649	NLRP1
2320	FGL1	2375	DYNLT2B	2430	CTTN	2485	NGFR	2540	KIF3A	2595	PROS1	2650	PDE4B
2321	CCDC47	2376	HGSNAT	2431	PEPD	2486	GJB2	2541	MAT2A	2596	LOC113664106	2651	TCF7
2322	GJB1	2377	GSTM3	2432	GPBAR1	2487	RAC2	2542	LAS1L	2597	NTRK2	2652	FOLR2
2323	EYA4	2378	SCNN1G	2433	DVL2	2488	PRKDC	2543	C1R	2598	APEH	2653	DAO
2324	SCD	2379	GBA2	2434	ARSB	2489	HK2	2544	CEP41	2599	STS	2654	WWOX
2325	AOX1	2380	HGD	2435	PRKACA	2490	MESD	2545	PRLR	2600	SNRPN	2655	SLC16A2
2326	LECT2	2381	MEGF10	2436	ARSA	2491	PDHX	2546	SLC19A2	2601	MRPS28	2656	TFPI
2327	PET100	2382	AK7	2437	ACACB	2492	CFTR-AS1	2547	FLVCR1	2602	MIR200C	2657	ZRSR2
2328	SLC22A1	2383	GCLC	2438	PRDM2	2493	CEACAM1	2548	FARSA	2603	UGCG	2658	GJA5
2329	FAN1	2384	CES2	2439	SIL1	2494	MOCOS	2549	MFSD2A	2604	PLP1	2659	SLC22A7
2330	TMEM165	2385	CYB5A	2440	GSTA2	2495	LYRM4	2550	CTNNA1	2605	MRAP	2660	TNC
2331	TMPRSS15	2386	SREBF2	2441	ARHGAP9	2496	SEC61A1	2551	OGG1	2606	PTRH2	2661	OCA2
2332	PTPN3	2387	GNPTAB	2442	LYST	2497	DPM2	2552	APTX	2607	AHSP	2662	P2RX7
2333	CCNH	2388	DHDDS	2443	RARS2	2498	SIRT3	2553	SMARCE1	2608	YY1AP1	2663	TAB2
2334	RBM20	2389	MYOT	2444	AFF3	2499	SARDH	2554	NDUFA9	2609	NRP1	2664	HAND2
2335	CSRP3	2390	IFT88	2445	LIMK1	2500	RGN	2555	PLOD1	2610	PDHA2	2665	PRKCQ
2336	SCNN1B	2391	ESR2	2446	FABP12	2501	MAN1B1	2556	KLKB1	2611	ERCC3	2666	KCNH1
2337	SULT2A1	2392	SEC23B	2447	COA3	2502	MARS2	2557	MGAM	2612	NDUFA10	2667	GAMT
2338	MIR29C	2393	LOC107980440	2448	TTC19	2503	DUSP6	2558	MN1	2613	PURB	2668	NLRP12
2339	DHCR7	2394	RARRES2	2449	STT3B	2504	ANGPTL8	2559	B4GALT1	2614	LIN28B	2669	XRCC3
2340	STAG2	2395	DNASE1L3	2450	AXIN2	2505	HEXB	2560	NPC1L1	2615	GALNS	2670	TRIB3
2341	CBFA2T2	2396	BTD	2451	CEP85L	2506	ADRA2C	2561	FDFT1	2616	TFDP1	2671	EVPL
2342	COLEC10	2397	GYPC	2452	FCN3	2507	CD82	2562	CRYAA	2617	ORM2	2672	GK
2343	PON3	2398	GALM	2453	UBD	2508	CFP	2563	NEFH	2618	TBX4	2673	CLPX
2344	IDS	2399	PFKM	2454	GHRH	2509	MIR154	2564	CUL3	2619	SDHAF1	2674	SUMF1
2345	LOC107982234	2400	AKR1C2	2455	PHYH	2510	RELN	2565	PTP4A3	2620	HOTAIRM	2675	GTF2IRD1
2346	LMOD3	2401	SLC46A1	2456	CYBC1	2511	FMO1	2566	ALDOA	2621	GPHN	2676	APOC1
2347	CLMP	2402	COG6	2457	NEUROD1	2512	BLK	2567	ATP5F1E	2622	ODC1	2677	KAT6B
2348	CERS1	2403	KRT5	2458	VRK2	2513	RFWD3	2568	TWIST1	2623	FOXA1	2678	COQ8B
2349	FGFR4	2404	GAS5	2459	MIR132	2514	MLLT6	2569	AFG3L2	2624	KISS1	2679	ACO1
2350	EPHX2	2405	DNM2	2460	PHOX2B	2515	MIR183	2570	MRPL3	2625	VPS11	2680	MEF2C
2351	LTBP3	2406	NAT1	2461	LYZ	2516	TUFM	2571	HSF2BP	2626	CAMP	2681	VPS37A
2352	AKR1C4	2407	HBG1	2462	KEAP1	2517	ABCG1	2572	AAAS	2627	ITK	2682	BCL2L2
2353	TTC21A	2408	KLF1	2463	H6PD	2518	PHGDH	2573	RNU4ATAC	2628	CLPB	2683	SGK1
2354	PPP2R3C	2409	MSR1	2464	INTU	2519	PLCG1	2574	SPTB	2629	WNT1	2684	MASP1
2355	KIAA0319L	2410	MIR107	2465	PREPL	2520	SLC7A9	2575	AK2	2630	PLTP	2685	PUS1
2356	IKZF3	2411	MAD2L2	2466	MIR26A1	2521	OXCT1	2576	ADRB3	2631	GPC4	2686	F13B
2357	FABP4	2412	PIK3R2	2467	LOC111674475	2522	GTF2I	2577	GREM1	2632	NEXN	2687	SPN
2358	HELLPAR	2413	SLC2A10	2468	SLCO1A2	2523	GRN	2578	SQOR	2633	MERTK	2688	ADCY6
2359	EPHB4	2414	MTHFD1	2469	F11	2524	POLD1	2579	GP1BB	2634	NAGA	2689	SMARCC2
2360	ATPAF2	2415	ANGPTL3	2470	TRIB1	2525	DNA2	2580	SFRP1	2635	NSD2	2690	TPP1
2361	PLIN2	2416	SPTA1	2471	CPB2	2526	NFS1	2581	SLC39A14	2636	PTPN1	2691	IAPP
2362	FLAD1	2417	JAGN1	2472	LRP1	2527	SLC39A4	2582	CYP8B1	2637	SULT1A1	2692	MCU
2363	MYOD1	2418	M6PR	2473	NFIX	2528	COL6A2	2583	SERPINB3	2638	RFC2	2693	MIR184
2364	MAFB	2419	PRKAA2	2474	AIFM1	2529	TTPA	2584	SALL1	2639	DEFB1	2694	ROBO1
2365	MAN2B1	2420	ID2	2475	FUCA1	2530	UTP4	2585	NCF2	2640	GLYAT	2695	HMGCS2

Table S2. 2913 ALF-related target genes (contrinued)

No.	Gene	No.	Gene	No.	Gene	No.	Gene	No.	Gene	No.	Gene	No.	Gene
2696	SLC4A4	2751	ACVR2B	2806	CASC15	2861	SEC61B						
2697	NPR2	2752	FADS1	2807	FADS2	2862	MRE11						
2698	ALG12	2753	WNT3A	2808	NDE1	2863	CHRNA1						
2699	HSD3B2	2754	APCS	2809	MSLN	2864	KLRC2						
2700	OGDH	2755	ACTG2	2810	SNAI2	2865	NCF4						
2701	HOXA13	2756	STARD3	2811	KCNB1	2866	KCNA5						
2702	PEG10	2757	APLNR	2812	ETS2	2867	WNT7A						
2703	GADD45G	2758	TBL2	2813	FTCD	2868	KRT20						
2704	DDAH1	2759	PCSK1	2814	LHX4	2869	TFDP3						
2705	KCNJ1	2760	PYY	2815	TPM3	2870	GNA11						
2706	ALG6	2761	NES	2816	MIR98	2871	MIR30B						
2707	RPS6KA1	2762	RTL1	2817	GSTO1	2872	CARD9						
2708	USP7	2763	PFAS	2818	CLIP2	2873	MIR28						
2709	NPR1	2764	PRKCZ	2819	ALG13	2874	REST						
2710	NEUROG3	2765	UGT2B17	2820	CPSF6	2875	CADM1						
2711	NDUFA12	2766	H4C11	2821	NF2	2876	SLC29A1						
2712	XRCC6	2767	HSPA1B	2822	ENTPD1	2877	MAPK9						
2713	PROX1	2768	PCNT	2823	IMMT	2878	NADK2						
2714	GPT2	2769	GAD1	2824	UBA5	2879	DECR1						
2715	CPT1B	2770	H4C14	2825	NNMT	2880	TREM2						
2716	DGAT2	2771	ITGA6	2826	S100A10	2881	SMAD9						
2717	HRG	2772	UCP3	2827	SORD	2882	RELB						
2718	BATF2	2773	DOLK	2828	PLAG1	2883	IFT20						
2719	H2AX	2774	ACAT1	2829	RARG	2884	RPS6						
2720	MIR148B	2775	TFF3	2830	HPR	2885	ZFPM2						
2721	RPL36A-HNRNPH2	2776	PLEC	2831	MAPKAPK2	2886	PGK1						
2722	CEP70	2777	GPX4	2832	LACC1	2887	MIR452						
2723	OLIG2	2778	IFT81	2833	AKR1B10	2888	MYOG						
2724	TTC36	2779	H4C9	2834	PALLD	2889	FLG						
2725	PDP1	2780	PARK7	2835	CD70	2890	LRRC41						
2726	SLC29A3	2781	FLNC-AS1	2836	MIR137	2891	PROCR						
2727	CATSPER2	2782	MYH3	2837	ECHS1	2892	DAG1						
2728	TRIM28	2783	CYP51A1	2838	ZEB1	2893	SERPINB2						
2729	SCTR	2784	EBF3	2839	IER3IP1	2894	GTF2A1L						
2730	MYLK2	2785	HDAC8	2840	HAO1	2895	STON1-GTF2A1L						
2731	HOXD13	2786	SSTR2	2841	MMP13	2896	AFM						
2732	COL18A1	2787	SOST	2842	MIR342	2897	GTF2A1						
2733	COX4I2	2788	TPD52	2843	PROZ	2898	STON1						
2734	MIR455	2789	SRA1	2844	PITRM1	2899	TBP						
2735	ADH4	2790	BCAP31	2845	AZIN1	2900	GTF2A2						
2736	ECI2	2791	NDUFA2	2846	DBT	2901	TBPL1						
2737	WNT3	2792	SCN1B	2847	CDC73	2902	CSNK2A1						
2738	BID	2793	ZNF276	2848	CCNE1	2903	POLR2L						
2739	TARDBP	2794	ATR	2849	CYP2J2	2904	ORC1						
2740	ABAT	2795	CSNK2A2	2850	EBF1	2905	RFX1						
2741	CKM	2796	H4C12	2851	SLC51B	2906	NAGK						
2742	NUDC	2797	H4C8	2852	FLT4	2907	IGFBP4						
2743	AGRN	2798	H4C6	2853	EWSR1	2908	IGFBP5						
2744	FBL	2799	H4C13	2854	IHH	2909	SLC38A5						
2745	ALDH9A1	2800	H4C5	2855	MIR139	2910	PFKFB3						
2746	CGA	2801	H4C15	2856	NDUFC2	2911	ARF6						
2747	ZIC2	2802	H4C4	2857	GARS1	2912	NT5E						
2748	NRTN	2803	NOS1AP	2858	AREG	2913	GSDMD						
2749	RPGR	2804	MAPKBP1	2859	MAGEA4								
2750	MLH3	2805	SLC8A1	2860	LOC112042785								
