# Supplementary material for: Identifying the Mechanism of Polygoni Cuspidati Rhizoma et Radix in Treating Acute Liver Failure Based on Network Pharmacology and Molecular Docking
Source: Gastroenterol Res Pract. 2022 Apr 8;2022:2021066. doi: 10.1155/2022/2021066 (PMC9012611; doi:10.1155/2022/2021066)
Supplement: Supplementary Materials — The 200 target genes interacting with the 10 bioactive components of PCRR, and the 2913 ALF-related target genes are provided in supplementary Tables S1 and S2, respectively. The 153 ALF-related PCRR target candidates are provided in supplementary Table S3. The results of the GO and KEGG pathway enrichment analyses are provided in supplementary Tables S4 and S5, respectively. [file 2021066.f1.zip › 2021066.f3.pdf]

Supplementary file

Table S3. 153 candidate target genes

No.	Gene	No.	Gene	No.	Gene	No.	Gene	No.	Gene
1	AKT1	32	MMP1	63	CDKN2A	94	IRF1	125	SERPINE1
2	APP	33	MMP2	64	CHEK2	95	KCNH2	126	SOD1
3	AR	34	MMP9	65	CHUK	96	MAOB	127	SPP1
4	BCL2L1	35	NCOA2	66	COL1A1	97	MGAM	128	STAT1
5	BIRC5	36	NFKBIA	67	COL3A1	98	MMP3	129	SULT1A1
6	CASP3	37	PCNA	68	CRP	99	MPO	130	SULT1A3
7	CASP7	38	PIK3CG	69	CTSD	100	MYC	131	TGFB1
8	CASP9	39	PPARG	70	CXCL10	101	NCF1	132	THBD
9	CCNB1	40	PRSS1	71	CXCL2	102	NFE2L2	133	VCAM1
10	CCND1	41	PTGS1	72	CXCL8	103	NOS3	134	VEGFA
11	CD40LG	42	PTGS2	73	CYP1A1	104	NQO1	135	XDH
12	CDK4	43	RB1	74	CYP1A2	105	NR1I2	136	SOD2
13	CDKN1A	44	RELA	75	CYP1B1	106	NR1I3	137	SOD3
14	DPP4	45	SLC2A4	76	CYP3A4	107	ODC1	138	HTR2A
15	EGFR	46	TNF	77	E2F1	108	PARP1	139	OPRM1
16	ERBB2	47	TOP1	78	EGF	109	PLAT	140	PGR
17	GSTP1	48	TOP2A	79	ERBB3	110	PLAU	141	SLC6A4
18	HMOX1	49	TP53	80	F10	111	PON1	142	CAT
19	HSP90AA1	50	TYR	81	F2	112	POR	143	ESR1
20	ICAM1	51	ABCG2	82	F3	113	PPARA	144	CA2
21	IFNG	52	ACACA	83	F7	114	PRKCA	145	CDK2
22	IL10	53	ACHE	84	FOS	115	PRKCB	146	CHEK1
23	IL2	54	ADRB2	85	GJA1	116	PTEN	147	GSK3B
24	IL4	55	AHR	86	GSTM1	117	RAF1	148	MAPK14
25	IL6	56	AKR1B1	87	HIF1A	118	RASA1	149	CCNA2
26	INSR	57	ALOX5	88	HK2	119	RASSF1	150	ESR2
27	JUN	58	BAX	89	HSPB1	120	RUNX1T1	151	NOS2
28	MAPK1	59	BCL2	90	IGF2	121	RUNX2	152	PTPN1
29	MCL1	60	CASP8	91	IGFBP3	122	RXRA	153	SLC6A3
30	MDM2	61	CAV1	92	IL1A	123	SCN5A		
31	MET	62	CCL2	93	IL1B	124	SELE		
