# Supplementary material for: Identifying the Mechanism of Polygoni Cuspidati Rhizoma et Radix in Treating Acute Liver Failure Based on Network Pharmacology and Molecular Docking
Source: Gastroenterol Res Pract. 2022 Apr 8;2022:2021066. doi: 10.1155/2022/2021066 (PMC9012611; doi:10.1155/2022/2021066)
Supplement: Supplementary Materials — The 200 target genes interacting with the 10 bioactive components of PCRR, and the 2913 ALF-related target genes are provided in supplementary Tables S1 and S2, respectively. The 153 ALF-related PCRR target candidates are provided in supplementary Table S3. The results of the GO and KEGG pathway enrichment analyses are provided in supplementary Tables S4 and S5, respectively. [file 2021066.f1.zip › 2021066.f4.pdf]

Supplementary file

Table S4. Gene ontology functional enrichment analysis

Term_ID	Term_description	ListHit	ListTotal	PopHit	PopTotal	GeneRatio	BgRatio	Enrichment_score	GeneIds	GeneSymbols	p-value	FDR_bh	Category
GO:0005737	cytoplasm	19	30	3289	14857	0.6	0.221	2.8608797	351;5294;760;5468;4780;5578;10499;9970;860;196;5728;840;7153;3320;332;1432;7422;1017;5921	APP;PIK3CG;CA2;PPARG;NFE2L2;PRKCA;NCOA2;NR1I3;RUNX2;AHR;PTEN;CASP7;TOP2A;HSP90AA1;BIRC5;MAPK14;VEGFA;CDK2;RASA1	1.44E-06	0.000128399	cellular_component
GO:0043536	positive regulation of blood vessel endothelial cell migration	3	30	14	13332	0.1	0.001	95.22857143	5578;7422;4780	PRKCA;VEGFA;NFE2L2	3.68E-06	0.000845272	biological_process
GO:0045931	positive regulation of mitotic cell cycle	3	30	15	13332	0.1	0.001	88.88	351;5578;332	APP;PRKCA;BIRC5	4.59E-06	0.000845272	biological_process
GO:0045944	positive regulation of transcription from RNA polymerase II promoter	8	30	588	13332	0.3	0.044	6.046258503	860;7153;5468;1432;7422;4780;196;10499	RUNX2;TOP2A;PPARG;MAPK14;VEGFA;NFE2L2;AHR;NCOA2	3.37E-05	0.003847452	biological_process
GO:0019904	protein domain specific binding	5	30	174	12391	0.2	0.014	11.86877395	860;6331;4780;10499;1017	RUNX2;SCN5A;NFE2L2;NCOA2;CDK2	5.53E-05	0.004598256	molecular_function
GO:0048010	vascular endothelial growth factor receptor signaling pathway	3	30	35	13332	0.1	0.003	38.09142857	3320;7422;1432	HSP90AA1;VEGFA;MAPK14	6.41E-05	0.003847452	biological_process
GO:0060373	regulation of ventricular cardiac muscle cell membrane depolarization	2	30	6	13332	0.1	0.000	148.1333333	6331;2697	SCN5A;GJA1	7.30E-05	0.003847452	biological_process
GO:0042475	odontogenesis of dentin-containing tooth	3	30	38	13332	0.1	0.003	35.08421053	860;6331;760	RUNX2;SCN5A;CA2	8.22E-05	0.003847452	biological_process
GO:0003707	steroid hormone receptor activity	3	30	36	12391	0.1	0.003	34.41944444	5468;9970;6256	PPARG;NR1I3;RXRA	8.66E-05	0.004598256	molecular_function
GO:0006915	apoptotic process	6	30	336	13332	0.2	0.025	7.935714286	351;1432;2697;196;5728;840	APP;MAPK14;GJA1;AHR;PTEN;CASP7	8.72E-05	0.003847452	biological_process
GO:0030278	regulation of ossification	2	30	7	13332	0.1	0.001	126.9714286	860;1432	RUNX2;MAPK14	0.000102079	0.003847452	biological_process
GO:0051923	sulfation	2	30	7	13332	0.1	0.001	126.9714286	6818;6817	SULT1A3;SULT1A1	0.000102079	0.003847452	biological_process
GO:0097110	scaffold protein binding	3	30	39	12391	0.1	0.003	31.77179487	3320;6331;2697	HSP90AA1;SCN5A;GJA1	0.000110358	0.004598256	molecular_function
GO:0006805	xenobiotic metabolic process	3	30	42	13332	0.1	0.003	31.74285714	6818;196;6817	SULT1A3;AHR;SULT1A1	0.00011125	0.003847452	biological_process
GO:0071498	cellular response to fluid shear stress	2	30	8	13332	0.1	0.001	111.1	760;4780	CA2;NFE2L2	0.000135915	0.003847452	biological_process
GO:0060371	regulation of atrial cardiac muscle cell membrane depolarization	2	30	8	13332	0.1	0.001	111.1	6331;2697	SCN5A;GJA1	0.000135915	0.003847452	biological_process
GO:0086014	atrial cardiac muscle cell action potential	2	30	8	13332	0.1	0.001	111.1	6331;2697	SCN5A;GJA1	0.000135915	0.003847452	biological_process
GO:1990782	protein tyrosine kinase binding	2	30	8	12391	0.1	0.001	103.2583333	3320;2697	HSP90AA1;GJA1	0.000157243	0.004913849	molecular_function
GO:0048514	blood vessel morphogenesis	2	30	10	13332	0.1	0.001	88.88	2697;5921	GJA1;RASA1	0.000217825	0.005009967	biological_process
GO:0045780	positive regulation of bone resorption	2	30	10	13332	0.1	0.001	88.88	5578;760	PRKCA;CA2	0.000217825	0.005009967	biological_process
GO:0006068	ethanol catabolic process	2	30	10	13332	0.1	0.001	88.88	6818;6817	SULT1A3;SULT1A1	0.000217825	0.005009967	biological_process
GO:0004062	aryl sulfotransferase activity	2	30	10	12391	0.1	0.001	82.60666667	6818;6817	SULT1A3;SULT1A1	0.000251952	0.006298798	molecular_function
GO:0060307	regulation of ventricular cardiac muscle cell membrane repolarization	2	30	11	13332	0.1	0.001	80.8	6331;2697	SCN5A;GJA1	0.000265858	0.005755037	biological_process
GO:0042629	mast cell granule	2	30	13	14857	0.1	0.001	76.18974359	5294;231	PIK3CG;AKR1B1	0.000303234	0.013493893	cellular_component
GO:0045766	positive regulation of angiogenesis	3	30	59	13332	0.1	0.004	22.59661017	5578;7422;4780	PRKCA;VEGFA;NFE2L2	0.000307015	0.006276744	biological_process
GO:0001525	angiogenesis	4	30	150	13332	0.1	0.011	11.85066667	5578;5294;7422;1432	PRKCA;PIK3CG;VEGFA;MAPK14	0.00033597	0.0065072	biological_process
GO:0044212	transcription regulatory region DNA binding	4	30	143	12391	0.1	0.012	11.55337995	860;196;5468;4780	RUNX2;AHR;PPARG;NFE2L2	0.00036912	0.007689991	molecular_function
GO:0035924	cellular response to vascular endothelial growth factor stimulus	2	30	13	13332	0.1	0.001	68.36923077	7422;1432	VEGFA;MAPK14	0.000375981	0.006588611	biological_process
GO:2000379	positive regulation of reactive oxygen species metabolic process	2	30	13	13332	0.1	0.001	68.36923077	1432;4780	MAPK14;NFE2L2	0.000375981	0.006588611	biological_process
GO:0000793	condensed chromosome	2	30	16	14857	0.1	0.001	61.90416667	7153;1017	TOP2A;CDK2	0.000464759	0.013787849	cellular_component
GO:0097718	disordered domain specific binding	2	30	14	12391	0.1	0.001	59.0047619	3320;2697	HSP90AA1;GJA1	0.000506443	0.00904362	molecular_function
GO:0046326	positive regulation of glucose import	2	30	17	13332	0.1	0.001	52.28235294	1432;4780	MAPK14;NFE2L2	0.000651897	0.01043035	biological_process
GO:0050427	3'-phosphoadenosine 5'-phosphosulfate metabolic process	2	30	17	13332	0.1	0.001	52.28235294	6818;6817	SULT1A3;SULT1A1	0.000651897	0.01043035	biological_process
GO:0038128	ERBB2 signaling pathway	2	30	18	13332	0.1	0.001	49.37777778	3320;5578	HSP90AA1;PRKCA	0.000732359	0.011229502	biological_process
GO:0032526	response to retinoic acid	2	30	20	13332	0.1	0.002	44.44	2697;5468	GJA1;PPARG	0.000906925	0.013349935	biological_process
GO:0043234	protein complex	5	30	395	14857	0.2	0.027	6.268776371	860;7153;3320;196;10499	RUNX2;TOP2A;HSP90AA1;AHR;NCOA2	0.001066493	0.022950876	cellular_component
GO:0004879	nuclear receptor activity	2	30	21	12391	0.1	0.002	39.33650794	196;5468	AHR;PPARG	0.001156467	0.017641686	molecular_function
GO:0008146	sulfotransferase activity	2	30	22	12391	0.1	0.002	37.54848485	6818;6817	SULT1A3;SULT1A1	0.001270201	0.017641686	molecular_function
GO:0005654	nucleoplasm	12	30	2356	14857	0.4	0.159	2.522410866	860;7153;10499;3320;862;4780;2697;5578;196;840;231;1017	RUNX2;TOP2A;NCOA2;HSP90AA1;RUNX1T1;NFE2L2;GJA1;PRKCA;AHR;CASP7;AKR1B1;CDK2	0.001289375	0.022950876	cellular_component
GO:0010595	positive regulation of endothelial cell migration	2	30	24	13332	0.1	0.002	37.03333333	5578;7422	PRKCA;VEGFA	0.001310081	0.017855919	biological_process
GO:0002062	chondrocyte differentiation	2	30	24	13332	0.1	0.002	37.03333333	860;1432	RUNX2;MAPK14	0.001310081	0.017855919	biological_process

Term_ID	Term_description	ListHit	ListTotal	PopHit	PopTotal	GeneRatio	BgRatio	Enrichment_score	GeneIds	GeneSymbols	p-value	FDR_bh	Category
GO:0018105	peptidyl-serine phosphorylation	3	30	103	13332	0.1	0.008	12.94368932	5578;1017;1432	PRKCA;CDK2;MAPK14	0.001562458	0.020535159	biological_process
GO:0045785	positive regulation of cell adhesion	2	30	27	13332	0.1	0.002	32.91851852	5578;7422	PRKCA;VEGFA	0.001659111	0.021053547	biological_process
GO:0051020	GTPase binding	2	30	26	12391	0.1	0.002	31.77179487	3320;5921	HSP90AA1;RASA1	0.001776364	0.022204544	molecular_function
GO:0006468	protein phosphorylation	4	30	235	13332	0.1	0.018	7.564255319	351;5578;1017;332	APP;PRKCA;CDK2;BIRC5	0.001798793	0.022065195	biological_process
GO:0042752	regulation of circadian rhythm	2	30	29	13332	0.1	0.002	30.64827586	7153;5468	TOP2A;PPARG	0.001913731	0.02271784	biological_process
GO:0043406	positive regulation of MAP kinase activity	2	30	30	13332	0.1	0.002	29.62666667	5294;7422	PIK3CG;VEGFA	0.002047564	0.022833443	biological_process
GO:0001938	positive regulation of endothelial cell proliferation	2	30	30	13332	0.1	0.002	29.62666667	5578;7422	PRKCA;VEGFA	0.002047564	0.022833443	biological_process
GO:0005667	transcription factor complex	3	30	127	14857	0.1	0.009	11.6984252	860;196;1017	RUNX2;AHR;CDK2	0.00209226	0.027950069	cellular_component
GO:0016328	lateral plasma membrane	2	30	35	14857	0.1	0.002	28.29904762	6331;2697	SCN5A;GJA1	0.002250162	0.027950069	cellular_component
GO:0032922	circadian regulation of gene expression	2	30	33	13332	0.1	0.002	26.93333333	10499;196	NCOA2;AHR	0.002474929	0.02678747	biological_process
GO:0014704	intercalated disc	2	30	37	14857	0.1	0.002	26.76936937	6331;2697	SCN5A;GJA1	0.002512366	0.027950069	cellular_component
GO:0045669	positive regulation of osteoblast differentiation	2	30	34	13332	0.1	0.003	26.14117647	860;2697	RUNX2;GJA1	0.002625944	0.027609923	biological_process
GO:0048511	rhythmic process	2	30	36	13332	0.1	0.003	24.68888889	7153;5468	TOP2A;PPARG	0.0029407	0.030060487	biological_process
GO:0005829	cytosol	15	30	3743	14857	0.5	0.252	1.984637991	860;5294;760;332;1432;3320;4780;6817;2697;5578;196;840;231;5921;6818	RUNX2;PIK3CG;CA2;BIRC5;MAPK14;HSP90AA1;NFE2L2;SULT1A1;GJA1;PRKCA;AHR;CASP7;AKR1B1;RASA1;SULT1A3	0.002953741	0.029209218	cellular_component
GO:0004672	protein kinase activity	3	30	122	12391	0.1	0.010	10.15655738	5578;5294;1017	PRKCA;PIK3CG;CDK2	0.003114046	0.034466637	molecular_function
GO:0008270	zinc ion binding	6	30	629	12391	0.2	0.051	3.93990461	332;760;5468;5578;6256;9970	BIRC5;CA2;PPARG;PRKCA;RXRA;NR1I3	0.003488726	0.034466637	molecular_function
GO:0043066	negative regulation of apoptotic process	4	30	284	13332	0.1	0.021	6.25915493	7422;231;332;5921	VEGFA;AKR1B1;BIRC5;RASA1	0.003570581	0.035512804	biological_process
GO:0003682	chromatin binding	4	30	266	12391	0.1	0.021	6.211027569	860;7153;10499;5468	RUNX2;TOP2A;NCOA2;PPARG	0.003666377	0.034466637	molecular_function
GO:0003700	DNA binding transcription factor activity	6	30	642	12391	0.2	0.052	3.860124611	860;5468;862;4780;196;6256	RUNX2;PPARG;RUNX1T1;NFE2L2;AHR;RXRA	0.003860263	0.034466637	molecular_function
GO:0030374	ligand-dependent nuclear receptor transcription coactivator activity	2	30	40	12391	0.1	0.003	20.65166667	10499;5468	NCOA2;PPARG	0.004174567	0.034788061	molecular_function
GO:0071356	cellular response to tumor necrosis factor	2	30	53	13332	0.1	0.004	16.76981132	1432;4780	MAPK14;NFE2L2	0.006281537	0.053821332	biological_process
GO:0016301	kinase activity	2	30	50	12391	0.1	0.004	16.52133333	5294;1017	PIK3CG;CDK2	0.006458635	0.050458086	molecular_function
GO:0045893	positive regulation of transcription, DNA-templated	4	30	339	13332	0.1	0.025	5.243657817	860;1017;196;5468	RUNX2;CDK2;AHR;PPARG	0.006676561	0.053821332	biological_process
GO:0007059	chromosome segregation	2	30	55	13332	0.1	0.004	16.16	7153;332	TOP2A;BIRC5	0.006750463	0.053821332	biological_process
GO:0030168	platelet activation	2	30	56	13332	0.1	0.004	15.87142857	5578;5294	PRKCA;PIK3CG	0.00699074	0.053821332	biological_process
GO:0071456	cellular response to hypoxia	2	30	56	13332	0.1	0.004	15.87142857	7422;4780	VEGFA;NFE2L2	0.00699074	0.053821332	biological_process
GO:0010628	positive regulation of gene expression	3	30	176	13332	0.1	0.013	7.575	860;2697;4780	RUNX2;GJA1;NFE2L2	0.007067176	0.053821332	biological_process
GO:0010389	regulation of G2/M transition of mitotic cell cycle	2	30	58	13332	0.1	0.004	15.32413793	3320;1017	HSP90AA1;CDK2	0.007482835	0.053821332	biological_process
GO:0045892	negative regulation of transcription, DNA-templated	4	30	358	13332	0.1	0.027	4.965363128	860;196;332;862	RUNX2;AHR;BIRC5;RUNX1T1	0.008069546	0.053821332	biological_process
GO:0001649	osteoblast differentiation	2	30	67	13332	0.1	0.005	13.26567164	860;2697	RUNX2;GJA1	0.009884274	0.053821332	biological_process
GO:0010468	regulation of gene expression	2	30	68	13332	0.1	0.005	13.07058824	196;1432	AHR;MAPK14	0.01016965	0.053821332	biological_process
GO:0007204	positive regulation of cytosolic calcium ion concentration	2	30	68	13332	0.1	0.005	13.07058824	2697;5294	GJA1;PIK3CG	0.01016965	0.053821332	biological_process
GO:0030522	intracellular receptor signaling pathway	1	30	5	13332	0.0	0.000	88.88	10499	NCOA2	0.011202277	0.053821332	biological_process
GO:0071467	cellular response to pH	1	30	5	13332	0.0	0.000	88.88	2697	GJA1	0.011202277	0.053821332	biological_process
GO:0003158	endothelium development	1	30	5	13332	0.0	0.000	88.88	2697	GJA1	0.011202277	0.053821332	biological_process
GO:0045040	protein import into mitochondrial outer membrane	1	30	5	13332	0.0	0.000	88.88	3320	HSP90AA1	0.011202277	0.053821332	biological_process
GO:0035148	tube formation	1	30	5	13332	0.0	0.000	88.88	7422	VEGFA	0.011202277	0.053821332	biological_process
GO:0060754	positive regulation of mast cell chemotaxis	1	30	5	13332	0.0	0.000	88.88	7422	VEGFA	0.011202277	0.053821332	biological_process
GO:1903169	regulation of calcium ion transmembrane transport	1	30	5	13332	0.0	0.000	88.88	5294	PIK3CG	0.011202277	0.053821332	biological_process
GO:0086045	membrane depolarization during AV node cell action potential	1	30	5	13332	0.0	0.000	88.88	6331	SCN5A	0.011202277	0.053821332	biological_process
GO:0002070	epithelial cell maturation	1	30	5	13332	0.0	0.000	88.88	2697	GJA1	0.011202277	0.053821332	biological_process
GO:0010818	T cell chemotaxis	1	30	5	13332	0.0	0.000	88.88	5294	PIK3CG	0.011202277	0.053821332	biological_process
GO:0003360	brainstem development	1	30	5	13332	0.0	0.000	88.88	6331	SCN5A	0.011202277	0.053821332	biological_process
GO:0045760	positive regulation of action potential	1	30	5	13332	0.0	0.000	88.88	6331	SCN5A	0.011202277	0.053821332	biological_process
GO:0031666	positive regulation of lipopolysaccharide-mediated signaling pathway	1	30	5	13332	0.0	0.000	88.88	5578	PRKCA	0.011202277	0.053821332	biological_process
GO:0007617	mating behavior	1	30	5	13332	0.0	0.000	88.88	351	APP	0.011202277	0.053821332	biological_process
GO:0046903	secretion	1	30	5	13332	0.0	0.000	88.88	760	CA2	0.011202277	0.053821332	biological_process
GO:0051146	striated muscle cell differentiation	1	30	5	13332	0.0	0.000	88.88	1432	MAPK14	0.011202277	0.053821332	biological_process

Term_ID	Term_description	ListHit	ListTotal	PopHit	PopTotal	GeneRatio	BgRatio	Enrichment_score	GeneIds	GeneSymbols	p-value	FDR_bh	Category
GO:0015867	ATP transport	1	30	5	13332	0.0	0.000	88.88	2697	GJA1	0.011202277	0.053821332	biological_process
GO:1901522	positive regulation of transcription from RNA polymerase II promoter involved in cellular response to chemical stimulus	1	30	5	13332	0.0	0.000	88.88	860	RUNX2	0.011202277	0.053821332	biological_process
GO:0045667	regulation of osteoblast differentiation	1	30	5	13332	0.0	0.000	88.88	860	RUNX2	0.011202277	0.053821332	biological_process
GO:0004032	alditol:NADP+ 1-oxidoreductase activity	1	30	5	12391	0.0	0.000	82.60666667	231	AKR1B1	0.01204902	0.065739172	molecular_function
GO:0086006	voltage-gated sodium channel activity involved in cardiac muscle cell action potential	1	30	5	12391	0.0	0.000	82.60666667	6331	SCN5A	0.01204902	0.065739172	molecular_function
GO:0043184	vascular endothelial growth factor receptor 2 binding	1	30	5	12391	0.0	0.000	82.60666667	7422	VEGFA	0.01204902	0.065739172	molecular_function
GO:0048273	mitogen-activated protein kinase p38 binding	1	30	5	12391	0.0	0.000	82.60666667	1432	MAPK14	0.01204902	0.065739172	molecular_function
GO:0035005	1-phosphatidylinositol-4-phosphate 3-kinase activity	1	30	5	12391	0.0	0.000	82.60666667	5294	PIK3CG	0.01204902	0.065739172	molecular_function
GO:0071253	connexin binding	1	30	5	12391	0.0	0.000	82.60666667	2697	GJA1	0.01204902	0.065739172	molecular_function
GO:0071682	endocytic vesicle lumen	1	30	6	14857	0.0	0.000	82.53888889	3320	HSP90AA1	0.012056524	0.089332063	cellular_component
GO:0043220	Schmidt-Lanterman incisure	1	30	6	14857	0.0	0.000	82.53888889	231	AKR1B1	0.012056524	0.089332063	cellular_component
GO:0019899	enzyme binding	3	30	203	12391	0.1	0.016	6.103940887	7153;6331;5578	TOP2A;SCN5A;PRKCA	0.012704289	0.065739172	molecular_function
GO:0002076	osteoblast development	1	30	6	13332	0.0	0.000	74.06666667	860	RUNX2	0.013428129	0.053821332	biological_process
GO:0071374	cellular response to parathyroid hormone stimulus	1	30	6	13332	0.0	0.000	74.06666667	2697	GJA1	0.013428129	0.053821332	biological_process
GO:0008210	estrogen metabolic process	1	30	6	13332	0.0	0.000	74.06666667	6817	SULT1A1	0.013428129	0.053821332	biological_process
GO:1901653	cellular response to peptide	1	30	6	13332	0.0	0.000	74.06666667	231	AKR1B1	0.013428129	0.053821332	biological_process
GO:0060044	negative regulation of cardiac muscle cell proliferation	1	30	6	13332	0.0	0.000	74.06666667	2697	GJA1	0.013428129	0.053821332	biological_process
GO:0006265	DNA topological change	1	30	6	13332	0.0	0.000	74.06666667	7153	TOP2A	0.013428129	0.053821332	biological_process
GO:1903206	negative regulation of hydrogen peroxide-induced cell death	1	30	6	13332	0.0	0.000	74.06666667	4780	NFE2L2	0.013428129	0.053821332	biological_process
GO:0032495	response to muramyl dipeptide	1	30	6	13332	0.0	0.000	74.06666667	1432	MAPK14	0.013428129	0.053821332	biological_process
GO:0003231	cardiac ventricle development	1	30	6	13332	0.0	0.000	74.06666667	6331	SCN5A	0.013428129	0.053821332	biological_process
GO:0009410	response to xenobiotic stimulus	1	30	6	13332	0.0	0.000	74.06666667	196	AHR	0.013428129	0.053821332	biological_process
GO:0055118	negative regulation of cardiac muscle contraction	1	30	6	13332	0.0	0.000	74.06666667	5294	PIK3CG	0.013428129	0.053821332	biological_process
GO:0051186	cofactor metabolic process	1	30	6	13332	0.0	0.000	74.06666667	3320	HSP90AA1	0.013428129	0.053821332	biological_process
GO:0035235	ionotropic glutamate receptor signaling pathway	1	30	6	13332	0.0	0.000	74.06666667	351	APP	0.013428129	0.053821332	biological_process
GO:0071223	cellular response to lipoteichoic acid	1	30	6	13332	0.0	0.000	74.06666667	1432	MAPK14	0.013428129	0.053821332	biological_process
GO:0042770	signal transduction in response to DNA damage	1	30	6	13332	0.0	0.000	74.06666667	1432	MAPK14	0.013428129	0.053821332	biological_process
GO:0035994	response to muscle stretch	1	30	6	13332	0.0	0.000	74.06666667	1432	MAPK14	0.013428129	0.053821332	biological_process
GO:0010831	positive regulation of myotube differentiation	1	30	6	13332	0.0	0.000	74.06666667	1432	MAPK14	0.013428129	0.053821332	biological_process
GO:0044070	regulation of anion transport	1	30	6	13332	0.0	0.000	74.06666667	760	CA2	0.013428129	0.053821332	biological_process
GO:0051272	positive regulation of cellular component movement	1	30	6	13332	0.0	0.000	74.06666667	7422	VEGFA	0.013428129	0.053821332	biological_process
GO:0086015	SA node cell action potential	1	30	6	13332	0.0	0.000	74.06666667	6331	SCN5A	0.013428129	0.053821332	biological_process
GO:0033010	paranodal junction	1	30	7	14857	0.0	0.000	70.74761905	231	AKR1B1	0.014052235	0.089332063	cellular_component
GO:0000307	cyclin-dependent protein kinase holoenzyme complex	1	30	7	14857	0.0	0.000	70.74761905	1017	CDK2	0.014052235	0.089332063	cellular_component
GO:0005916	fascia adherens	1	30	7	14857	0.0	0.000	70.74761905	2697	GJA1	0.014052235	0.089332063	cellular_component
GO:0001162	RNA polymerase II intronic transcription regulatory region sequence-specific DNA binding	1	30	6	12391	0.0	0.000	68.83888889	10499	NCOA2	0.014441926	0.065739172	molecular_function
GO:0019870	potassium channel inhibitor activity	1	30	6	12391	0.0	0.000	68.83888889	5921	RASA1	0.014441926	0.065739172	molecular_function
GO:0042826	histone deacetylase binding	2	30	77	12391	0.1	0.006	10.72813853	3320;7153	HSP90AA1;TOP2A	0.014816299	0.065739172	molecular_function
GO:0030165	PDZ domain binding	2	30	77	12391	0.1	0.006	10.72813853	2697;5728	GJA1;PTEN	0.014816299	0.065739172	molecular_function
GO:0000086	G2/M transition of mitotic cell cycle	2	30	83	13332	0.1	0.006	10.70843373	3320;332	HSP90AA1;BIRC5	0.014878767	0.053821332	biological_process
GO:0005125	cytokine activity	2	30	79	12391	0.1	0.006	10.45654008	3565;7422	IL4;VEGFA	0.015554658	0.065739172	molecular_function
GO:0016572	histone phosphorylation	1	30	7	13332	0.0	0.001	63.48571429	1017	CDK2	0.015649137	0.053821332	biological_process
GO:0016264	gap junction assembly	1	30	7	13332	0.0	0.001	63.48571429	2697	GJA1	0.015649137	0.053821332	biological_process
GO:0006700	C21-steroid hormone biosynthetic process	1	30	7	13332	0.0	0.001	63.48571429	231	AKR1B1	0.015649137	0.053821332	biological_process
GO:1903351	cellular response to dopamine	1	30	7	13332	0.0	0.001	63.48571429	6818	SULT1A3	0.015649137	0.053821332	biological_process
GO:0019395	fatty acid oxidation	1	30	7	13332	0.0	0.001	63.48571429	1432	MAPK14	0.015649137	0.053821332	biological_process
GO:0044597	daunorubicin metabolic process	1	30	7	13332	0.0	0.001	63.48571429	231	AKR1B1	0.015649137	0.053821332	biological_process
GO:0044598	doxorubicin metabolic process	1	30	7	13332	0.0	0.001	63.48571429	231	AKR1B1	0.015649137	0.053821332	biological_process
GO:0035767	endothelial cell chemotaxis	1	30	7	13332	0.0	0.001	63.48571429	7422	VEGFA	0.015649137	0.053821332	biological_process
GO:0090050	positive regulation of cell migration involved in sprouting angiogenesis	1	30	7	13332	0.0	0.001	63.48571429	7422	VEGFA	0.015649137	0.053821332	biological_process
GO:1903071	positive regulation of ER-associated ubiquitin-dependent protein catabolic process	1	30	7	13332	0.0	0.001	63.48571429	4780	NFE2L2	0.015649137	0.053821332	biological_process
GO:0030225	macrophage differentiation	1	30	7	13332	0.0	0.001	63.48571429	7422	VEGFA	0.015649137	0.053821332	biological_process
GO:1902305	regulation of sodium ion transmembrane transport	1	30	7	13332	0.0	0.001	63.48571429	6331	SCN5A	0.015649137	0.053821332	biological_process
GO:0061045	negative regulation of wound healing	1	30	7	13332	0.0	0.001	63.48571429	2697	GJA1	0.015649137	0.053821332	biological_process

Term_ID	Term_description	ListHit	ListTotal	PopHit	PopTotal	GeneRatio	BgRatio	Enrichment_score	GeneIds	GeneSymbols	p-value	FDR_bh	Category
GO:0014894	response to denervation involved in regulation of muscle adaptation	1	30	7	13332	0.0	0.001	63.48571429	6331	SCN5A	0.015649137	0.053821332	biological_process
GO:0010759	positive regulation of macrophage chemotaxis	1	30	7	13332	0.0	0.001	63.48571429	1432	MAPK14	0.015649137	0.053821332	biological_process
GO:2000810	regulation of bicellular tight junction assembly	1	30	7	13332	0.0	0.001	63.48571429	2697	GJA1	0.015649137	0.053821332	biological_process
GO:0045651	positive regulation of macrophage differentiation	1	30	7	13332	0.0	0.001	63.48571429	5578	PRKCA	0.015649137	0.053821332	biological_process
GO:0002675	positive regulation of acute inflammatory response	1	30	7	13332	0.0	0.001	63.48571429	5294	PIK3CG	0.015649137	0.053821332	biological_process
GO:0046697	decidualization	1	30	7	13332	0.0	0.001	63.48571429	2697	GJA1	0.015649137	0.053821332	biological_process
GO:0008083	growth factor activity	2	30	82	12391	0.1	0.007	10.07398374	3565;7422	IL4;VEGFA	0.016691422	0.065739172	molecular_function
GO:0008094	DNA-dependent ATPase activity	1	30	7	12391	0.0	0.001	59.0047619	7153	TOP2A	0.016829228	0.065739172	molecular_function
GO:0030911	TPR domain binding	1	30	7	12391	0.0	0.001	59.0047619	3320	HSP90AA1	0.016829228	0.065739172	molecular_function
GO:0005243	gap junction channel activity	1	30	7	12391	0.0	0.001	59.0047619	2697	GJA1	0.016829228	0.065739172	molecular_function
GO:0050872	white fat cell differentiation	1	30	8	13332	0.0	0.001	55.55	5468	PPARG	0.017865312	0.055247351	biological_process
GO:0048545	response to steroid hormone	1	30	8	13332	0.0	0.001	55.55	760	CA2	0.017865312	0.055247351	biological_process
GO:0046855	inositol phosphate dephosphorylation	1	30	8	13332	0.0	0.001	55.55	5728	PTEN	0.017865312	0.055247351	biological_process
GO:0007178	transmembrane receptor protein serine/threonine kinase signaling pathway	1	30	8	13332	0.0	0.001	55.55	1432	MAPK14	0.017865312	0.055247351	biological_process
GO:0006878	cellular copper ion homeostasis	1	30	8	13332	0.0	0.001	55.55	351	APP	0.017865312	0.055247351	biological_process
GO:0042026	protein refolding	1	30	8	13332	0.0	0.001	55.55	3320	HSP90AA1	0.017865312	0.055247351	biological_process
GO:0009268	response to pH	1	30	8	13332	0.0	0.001	55.55	760	CA2	0.017865312	0.055247351	biological_process
GO:0045475	locomotor rhythm	1	30	8	13332	0.0	0.001	55.55	10499	NCOA2	0.017865312	0.055247351	biological_process
GO:0030949	positive regulation of vascular endothelial growth factor receptor signaling pathway	1	30	8	13332	0.0	0.001	55.55	7422	VEGFA	0.017865312	0.055247351	biological_process
GO:2000279	negative regulation of DNA biosynthetic process	1	30	8	13332	0.0	0.001	55.55	2697	GJA1	0.017865312	0.055247351	biological_process
GO:0033628	regulation of cell adhesion mediated by integrin	1	30	8	13332	0.0	0.001	55.55	5294	PIK3CG	0.017865312	0.055247351	biological_process
GO:0016322	neuron remodeling	1	30	8	13332	0.0	0.001	55.55	351	APP	0.017865312	0.055247351	biological_process
GO:0005524	ATP binding	7	30	1153	12391	0.2	0.093	2.507574443	860;7153;5294;1432;3320;5578;1017	RUNX2;TOP2A;PIK3CG;MAPK14;HSP90AA1;PRKCA;CDK2	0.017929715	0.067915586	molecular_function
GO:0043209	myelin sheath	2	30	104	14857	0.1	0.007	9.523717949	3320;760	HSP90AA1;CA2	0.018583807	0.110263919	cellular_component
GO:0006357	regulation of transcription from RNA polymerase II promoter	3	30	254	13332	0.1	0.019	5.248818898	196;6648;1432	AHR;SOD2;MAPK14	0.018989673	0.056398564	biological_process
GO:0005161	platelet-derived growth factor receptor binding	1	30	8	12391	0.0	0.001	51.62916667	7422	VEGFA	0.01921094	0.068610501	molecular_function
GO:0001223	transcription coactivator binding	1	30	8	12391	0.0	0.001	51.62916667	196	AHR	0.01921094	0.068610501	molecular_function
GO:0050930	induction of positive chemotaxis	1	30	9	13332	0.0	0.001	49.37777778	7422	VEGFA	0.020076663	0.056398564	biological_process
GO:0045672	positive regulation of osteoclast differentiation	1	30	9	13332	0.0	0.001	49.37777778	760	CA2	0.020076663	0.056398564	biological_process
GO:0002009	morphogenesis of an epithelium	1	30	9	13332	0.0	0.001	49.37777778	760	CA2	0.020076663	0.056398564	biological_process
GO:0001894	tissue homeostasis	1	30	9	13332	0.0	0.001	49.37777778	231	AKR1B1	0.020076663	0.056398564	biological_process
GO:0042098	T cell proliferation	1	30	9	13332	0.0	0.001	49.37777778	5294	PIK3CG	0.020076663	0.056398564	biological_process
GO:0097755	positive regulation of blood vessel diameter	1	30	9	13332	0.0	0.001	49.37777778	2697	GJA1	0.020076663	0.056398564	biological_process
GO:0030224	monocyte differentiation	1	30	9	13332	0.0	0.001	49.37777778	7422	VEGFA	0.020076663	0.056398564	biological_process
GO:0007512	adult heart development	1	30	9	13332	0.0	0.001	49.37777778	2697	GJA1	0.020076663	0.056398564	biological_process
GO:0050918	positive chemotaxis	1	30	9	13332	0.0	0.001	49.37777778	7422	VEGFA	0.020076663	0.056398564	biological_process
GO:0086005	ventricular cardiac muscle cell action potential	1	30	9	13332	0.0	0.001	49.37777778	6331	SCN5A	0.020076663	0.056398564	biological_process
GO:0043303	mast cell degranulation	1	30	9	13332	0.0	0.001	49.37777778	5294	PIK3CG	0.020076663	0.056398564	biological_process
GO:0008360	regulation of cell shape	2	30	100	13332	0.1	0.008	8.888	7422;5921	VEGFA;RASA1	0.021138662	0.057391439	biological_process
GO:0015075	ion transmembrane transporter activity	1	30	9	12391	0.0	0.001	45.89259259	2697	GJA1	0.021587075	0.072402975	molecular_function
GO:0050998	nitric-oxide synthase binding	1	30	9	12391	0.0	0.001	45.89259259	6331	SCN5A	0.021587075	0.072402975	molecular_function
GO:0031235	intrinsic component of the cytoplasmic side of the plasma membrane	1	30	11	14857	0.0	0.001	45.02121212	5921	RASA1	0.021996179	0.118530577	cellular_component
GO:0008201	heparin binding	2	30	95	12391	0.1	0.008	8.695438596	351;7422	APP;VEGFA	0.022010504	0.072402975	molecular_function
GO:0086064	cell communication by electrical coupling involved in cardiac conduction	1	30	10	13332	0.0	0.001	44.44	2697	GJA1	0.0222832	0.057391439	biological_process
GO:0002042	cell migration involved in sprouting angiogenesis	1	30	10	13332	0.0	0.001	44.44	7422	VEGFA	0.0222832	0.057391439	biological_process
GO:0000712	resolution of meiotic recombination intermediates	1	30	10	13332	0.0	0.001	44.44	7153	TOP2A	0.0222832	0.057391439	biological_process
GO:0032793	positive regulation of CREB transcription factor activity	1	30	10	13332	0.0	0.001	44.44	7422	VEGFA	0.0222832	0.057391439	biological_process
GO:0031396	regulation of protein ubiquitination	1	30	10	13332	0.0	0.001	44.44	3320	HSP90AA1	0.0222832	0.057391439	biological_process
GO:0032332	positive regulation of chondrocyte differentiation	1	30	10	13332	0.0	0.001	44.44	860	RUNX2	0.0222832	0.057391439	biological_process
GO:0030833	regulation of actin filament polymerization	1	30	10	13332	0.0	0.001	44.44	5921	RASA1	0.0222832	0.057391439	biological_process
GO:0032230	positive regulation of synaptic transmission, GABAergic	1	30	10	13332	0.0	0.001	44.44	760	CA2	0.0222832	0.057391439	biological_process
GO:0051895	negative regulation of focal adhesion assembly	1	30	10	13332	0.0	0.001	44.44	5728	PTEN	0.0222832	0.057391439	biological_process

Term_ID	Term_description	ListHit	ListTotal	PopHit	PopTotal	GeneRatio	BgRatio	Enrichment_score	GeneIds	GeneSymbols	p-value	FDR_bh	Category
GO:0046677	response to antibiotic	1	30	10	13332	0.0	0.001	44.44	3320	HSP90AA1	0.0222832	0.057391439	biological_process
GO:0007507	heart development	2	30	104	13332	0.1	0.008	8.546153846	2697;5728	GJA1;PTEN	0.022746178	0.057391439	biological_process
GO:0004697	protein kinase C activity	1	30	10	12391	0.0	0.001	41.30333333	5578	PRKCA	0.023957644	0.073041597	molecular_function
GO:0017134	fibroblast growth factor binding	1	30	10	12391	0.0	0.001	41.30333333	6331	SCN5A	0.023957644	0.073041597	molecular_function
GO:0035257	nuclear hormone receptor binding	1	30	10	12391	0.0	0.001	41.30333333	10499	NCOA2	0.023957644	0.073041597	molecular_function
GO:0001518	voltage-gated sodium channel complex	1	30	12	14857	0.0	0.001	41.26944444	6331	SCN5A	0.023972476	0.118530577	cellular_component
GO:0005921	gap junction	1	30	12	14857	0.0	0.001	41.26944444	2697	GJA1	0.023972476	0.118530577	cellular_component
GO:0008284	positive regulation of cell proliferation	3	30	279	13332	0.1	0.021	4.778494624	860;1017;7422	RUNX2;CDK2;VEGFA	0.024265981	0.057391439	biological_process
GO:0042307	positive regulation of protein import into nucleus	1	30	11	13332	0.0	0.001	40.4	1432	MAPK14	0.024484935	0.057391439	biological_process
GO:0002063	chondrocyte development	1	30	11	13332	0.0	0.001	40.4	860	RUNX2	0.024484935	0.057391439	biological_process
GO:0001958	endochondral ossification	1	30	11	13332	0.0	0.001	40.4	860	RUNX2	0.024484935	0.057391439	biological_process
GO:0007004	telomere maintenance via telomerase	1	30	11	13332	0.0	0.001	40.4	3320	HSP90AA1	0.024484935	0.057391439	biological_process
GO:0060968	regulation of gene silencing	1	30	11	13332	0.0	0.001	40.4	1017	CDK2	0.024484935	0.057391439	biological_process
GO:0006801	superoxide metabolic process	1	30	11	13332	0.0	0.001	40.4	6648	SOD2	0.024484935	0.057391439	biological_process
GO:0001953	negative regulation of cell-matrix adhesion	1	30	11	13332	0.0	0.001	40.4	5921	RASA1	0.024484935	0.057391439	biological_process
GO:0086004	regulation of cardiac muscle cell contraction	1	30	11	13332	0.0	0.001	40.4	6331	SCN5A	0.024484935	0.057391439	biological_process
GO:0051131	chaperone-mediated protein complex assembly	1	30	11	13332	0.0	0.001	40.4	3320	HSP90AA1	0.024484935	0.057391439	biological_process
GO:0050999	regulation of nitric-oxide synthase activity	1	30	11	13332	0.0	0.001	40.4	3320	HSP90AA1	0.024484935	0.057391439	biological_process
GO:2000352	negative regulation of endothelial cell apoptotic process	1	30	11	13332	0.0	0.001	40.4	4780	NFE2L2	0.024484935	0.057391439	biological_process
GO:0001816	cytokine production	1	30	11	13332	0.0	0.001	40.4	5294	PIK3CG	0.024484935	0.057391439	biological_process
GO:0043254	regulation of protein complex assembly	1	30	11	13332	0.0	0.001	40.4	3320	HSP90AA1	0.024484935	0.057391439	biological_process
GO:0006897	endocytosis	2	30	111	13332	0.1	0.008	8.007207207	351;5294	APP;PIK3CG	0.025677266	0.059508668	biological_process
GO:0035326	enhancer binding	1	30	11	12391	0.0	0.001	37.54848485	196	AHR	0.02632266	0.076282279	molecular_function
GO:0070182	DNA polymerase binding	1	30	11	12391	0.0	0.001	37.54848485	3320	HSP90AA1	0.02632266	0.076282279	molecular_function
GO:0010906	regulation of glucose metabolic process	1	30	12	13332	0.0	0.001	37.03333333	10499	NCOA2	0.026681876	0.059508668	biological_process
GO:0009235	cobalamin metabolic process	1	30	12	13332	0.0	0.001	37.03333333	5644	PRSS1	0.026681876	0.059508668	biological_process
GO:0086012	membrane depolarization during cardiac muscle cell action potential	1	30	12	13332	0.0	0.001	37.03333333	6331	SCN5A	0.026681876	0.059508668	biological_process
GO:0043403	skeletal muscle tissue regeneration	1	30	12	13332	0.0	0.001	37.03333333	2697	GJA1	0.026681876	0.059508668	biological_process
GO:1901741	positive regulation of myoblast fusion	1	30	12	13332	0.0	0.001	37.03333333	1432	MAPK14	0.026681876	0.059508668	biological_process
GO:0060045	positive regulation of cardiac muscle cell proliferation	1	30	12	13332	0.0	0.001	37.03333333	1432	MAPK14	0.026681876	0.059508668	biological_process
GO:0043434	response to peptide hormone	1	30	12	13332	0.0	0.001	37.03333333	2697	GJA1	0.026681876	0.059508668	biological_process
GO:0005634	nucleus	14	30	4252	14857	0.5	0.286	1.630589526	860;7153;5468;332;1432;3320;4780;862;196;840;10499;1017;9970;6256	RUNX2;TOP2A;PPARG;BIRC5;MAPK14;HSP90AA1;NFE2L2;RUNX1T1;AHR;CASP7;NCOA2;CDK2;NR1I3;RXRA	0.027022966	0.126581261	cellular_component
GO:0008301	DNA binding, bending	1	30	12	12391	0.0	0.001	34.41944444	7153	TOP2A	0.028682137	0.076282279	molecular_function
GO:0043027	cysteine-type endopeptidase inhibitor activity involved in apoptotic [illegible]	1	30	12	12391	0.0	0.001	34.41944444	332	BIRC5	0.028682137	0.076282279	molecular_function
GO:0042056	chemoattractant activity	1	30	12	12391	0.0	0.001	34.41944444	7422	VEGFA	0.028682137	0.076282279	molecular_function
GO:0048037	cofactor binding	1	30	12	12391	0.0	0.001	34.41944444	332	BIRC5	0.028682137	0.076282279	molecular_function
GO:0010613	positive regulation of cardiac muscle hypertrophy	1	30	13	13332	0.0	0.001	34.18461538	5578	PRKCA	0.028874034	0.060031889	biological_process
GO:0009408	response to heat	1	30	13	13332	0.0	0.001	34.18461538	3320	HSP90AA1	0.028874034	0.060031889	biological_process
GO:0031954	positive regulation of protein autophosphorylation	1	30	13	13332	0.0	0.001	34.18461538	7422	VEGFA	0.028874034	0.060031889	biological_process
GO:0045995	regulation of embryonic development	1	30	13	13332	0.0	0.001	34.18461538	4780	NFE2L2	0.028874034	0.060031889	biological_process
GO:0070371	ERK1 and ERK2 cascade	1	30	13	13332	0.0	0.001	34.18461538	6818	SULT1A3	0.028874034	0.060031889	biological_process
GO:0007212	dopamine receptor signaling pathway	1	30	13	13332	0.0	0.001	34.18461538	6818	SULT1A3	0.028874034	0.060031889	biological_process
GO:0031663	lipopolysaccharide-mediated signaling pathway	1	30	13	13332	0.0	0.001	34.18461538	1432	MAPK14	0.028874034	0.060031889	biological_process
GO:0040014	regulation of multicellular organism growth	1	30	13	13332	0.0	0.001	34.18461538	351	APP	0.028874034	0.060031889	biological_process
GO:0001502	cartilage condensation	1	30	13	13332	0.0	0.001	34.18461538	1432	MAPK14	0.028874034	0.060031889	biological_process
GO:0000077	DNA damage checkpoint	1	30	13	13332	0.0	0.001	34.18461538	1432	MAPK14	0.028874034	0.060031889	biological_process
GO:0010765	positive regulation of sodium ion transport	1	30	13	13332	0.0	0.001	34.18461538	6331	SCN5A	0.028874034	0.060031889	biological_process
GO:0045648	positive regulation of erythrocyte differentiation	1	30	13	13332	0.0	0.001	34.18461538	1432	MAPK14	0.028874034	0.060031889	biological_process
GO:0030660	Golgi-associated vesicle membrane	1	30	15	14857	0.0	0.001	33.01555556	2697	GJA1	0.029878234	0.132958141	cellular_component
GO:0004089	carbonate dehydratase activity	1	30	13	12391	0.0	0.001	31.77179487	760	CA2	0.031036086	0.079173689	molecular_function
GO:0004438	phosphatidylinositol-3-phosphatase activity	1	30	13	12391	0.0	0.001	31.77179487	5728	PTEN	0.031036086	0.079173689	molecular_function
GO:0030316	osteoclast differentiation	1	30	14	13332	0.0	0.001	31.74285714	1432	MAPK14	0.031061419	0.060161063	biological_process
GO:0045907	positive regulation of vasoconstriction	1	30	14	13332	0.0	0.001	31.74285714	2697	GJA1	0.031061419	0.060161063	biological_process
GO:0016310	phosphorylation	1	30	14	13332	0.0	0.001	31.74285714	5294	PIK3CG	0.031061419	0.060161063	biological_process

Term_ID	Term_description	ListHit	ListTotal	PopHit	PopTotal	GeneRatio	BgRatio	Enrichment_score	GeneIds	GeneSymbols	p-value	FDR_bh	Category
GO:0051924	regulation of calcium ion transport	1	30	14	13332	0.0	0.001	31.74285714	2697	GJA1	0.031061419	0.060161063	biological_process
GO:0010667	negative regulation of cardiac muscle cell apoptotic process	1	30	14	13332	0.0	0.001	31.74285714	4780	NFE2L2	0.031061419	0.060161063	biological_process
GO:0030500	regulation of bone mineralization	1	30	14	13332	0.0	0.001	31.74285714	2697	GJA1	0.031061419	0.060161063	biological_process
GO:0007099	centriole replication	1	30	14	13332	0.0	0.001	31.74285714	1017	CDK2	0.031061419	0.060161063	biological_process
GO:0098586	cellular response to virus	1	30	14	13332	0.0	0.001	31.74285714	1432	MAPK14	0.031061419	0.060161063	biological_process
GO:0010975	regulation of neuron projection development	1	30	14	13332	0.0	0.001	31.74285714	5728	PTEN	0.031061419	0.060161063	biological_process
GO:0030217	T cell differentiation	1	30	14	13332	0.0	0.001	31.74285714	860	RUNX2	0.031061419	0.060161063	biological_process
GO:0070555	response to interleukin-1	1	30	14	13332	0.0	0.001	31.74285714	5578	PRKCA	0.031061419	0.060161063	biological_process
GO:0051899	membrane depolarization	1	30	14	13332	0.0	0.001	31.74285714	6331	SCN5A	0.031061419	0.060161063	biological_process
GO:0086002	cardiac muscle cell action potential involved in contraction	1	30	14	13332	0.0	0.001	31.74285714	6331	SCN5A	0.031061419	0.060161063	biological_process
GO:0035556	intracellular signal transduction	3	30	308	13332	0.1	0.023	4.328571429	5578;5921;1432	PRKCA;RASA1;MAPK14	0.031290254	0.060286982	biological_process
GO:0001937	negative regulation of endothelial cell proliferation	1	30	15	13332	0.0	0.001	29.62666667	2697	GJA1	0.03324404	0.062100542	biological_process
GO:0002092	positive regulation of receptor internalization	1	30	15	13332	0.0	0.001	29.62666667	7422	VEGFA	0.03324404	0.062100542	biological_process
GO:0031334	positive regulation of protein complex assembly	1	30	15	13332	0.0	0.001	29.62666667	7422	VEGFA	0.03324404	0.062100542	biological_process
GO:0015721	bile acid and bile salt transport	1	30	15	13332	0.0	0.001	29.62666667	10499	NCOA2	0.03324404	0.062100542	biological_process
GO:0060135	maternal process involved in female pregnancy	1	30	15	13332	0.0	0.001	29.62666667	231	AKR1B1	0.03324404	0.062100542	biological_process
GO:0010033	response to organic substance	1	30	15	13332	0.0	0.001	29.62666667	1017	CDK2	0.03324404	0.062100542	biological_process
GO:0023026	MHC class II protein complex binding	1	30	14	12391	0.0	0.001	29.50238095	3320	HSP90AA1	0.03338452	0.081198754	molecular_function
GO:0004033	aldo-keto reductase (NADP) activity	1	30	14	12391	0.0	0.001	29.50238095	231	AKR1B1	0.03338452	0.081198754	molecular_function
GO:0005771	multivesicular body	1	30	17	14857	0.0	0.001	29.13137255	2697	GJA1	0.033796199	0.136720986	cellular_component
GO:0005922	connexin complex	1	30	17	14857	0.0	0.001	29.13137255	2697	GJA1	0.033796199	0.136720986	cellular_component
GO:0045663	positive regulation of myoblast differentiation	1	30	16	13332	0.0	0.001	27.775	1432	MAPK14	0.035421909	0.063898346	biological_process
GO:0016358	dendrite development	1	30	16	13332	0.0	0.001	27.775	351	APP	0.035421909	0.063898346	biological_process
GO:0021537	telencephalon development	1	30	16	13332	0.0	0.001	27.775	6331	SCN5A	0.035421909	0.063898346	biological_process
GO:0008202	steroid metabolic process	1	30	16	13332	0.0	0.001	27.775	6818	SULT1A3	0.035421909	0.063898346	biological_process
GO:0043491	protein kinase B signaling	1	30	16	13332	0.0	0.001	27.775	5728	PTEN	0.035421909	0.063898346	biological_process
GO:0014065	phosphatidylinositol 3-kinase signaling	1	30	16	13332	0.0	0.001	27.775	5294	PIK3CG	0.035421909	0.063898346	biological_process
GO:0045429	positive regulation of nitric oxide biosynthetic process	1	30	16	13332	0.0	0.001	27.775	3320	HSP90AA1	0.035421909	0.063898346	biological_process
GO:0046983	protein dimerization activity	2	30	123	12391	0.1	0.010	6.71598916	10499;196	NCOA2;AHR	0.035478792	0.081198754	molecular_function
GO:0001968	fibronectin binding	1	30	15	12391	0.0	0.001	27.53555556	7422	VEGFA	0.035727452	0.081198754	molecular_function
GO:0004693	cyclin-dependent protein serine/threonine kinase activity	1	30	15	12391	0.0	0.001	27.53555556	1017	CDK2	0.035727452	0.081198754	molecular_function
GO:0016922	ligand-dependent nuclear receptor binding	1	30	15	12391	0.0	0.001	27.53555556	10499	NCOA2	0.035727452	0.081198754	molecular_function
GO:0042113	B cell activation	1	30	17	13332	0.0	0.001	26.14117647	3565	IL4	0.037595035	0.066835618	biological_process
GO:0045879	negative regulation of smoothened signaling pathway	1	30	17	13332	0.0	0.001	26.14117647	860	RUNX2	0.037595035	0.066835618	biological_process
GO:0097194	execution phase of apoptosis	1	30	17	13332	0.0	0.001	26.14117647	840	CASP7	0.037595035	0.066835618	biological_process
GO:0046914	transition metal ion binding	1	30	16	12391	0.0	0.001	25.81458333	351	APP	0.038064893	0.08496628	molecular_function
GO:0005876	spindle microtubule	1	30	20	14857	0.0	0.001	24.76166667	332	BIRC5	0.039644481	0.153406904	cellular_component
GO:0048863	stem cell differentiation	1	30	18	13332	0.0	0.001	24.68888889	860	RUNX2	0.039763428	0.069350433	biological_process
GO:0030593	neutrophil chemotaxis	1	30	18	13332	0.0	0.001	24.68888889	5294	PIK3CG	0.039763428	0.069350433	biological_process
GO:0046856	phosphatidylinositol dephosphorylation	1	30	18	13332	0.0	0.001	24.68888889	5728	PTEN	0.039763428	0.069350433	biological_process
GO:0051894	positive regulation of focal adhesion assembly	1	30	18	13332	0.0	0.001	24.68888889	7422	VEGFA	0.039763428	0.069350433	biological_process
GO:0030506	ankyrin binding	1	30	17	12391	0.0	0.001	24.29607843	6331	SCN5A	0.040396857	0.088589599	molecular_function
GO:0045070	positive regulation of viral genome replication	1	30	19	13332	0.0	0.001	23.38947368	7153	TOP2A	0.041927097	0.07176359	biological_process
GO:0010043	response to zinc ion	1	30	19	13332	0.0	0.001	23.38947368	760	CA2	0.041927097	0.07176359	biological_process
GO:0046580	negative regulation of Ras protein signal transduction	1	30	19	13332	0.0	0.001	23.38947368	5921	RASA1	0.041927097	0.07176359	biological_process
GO:0051898	negative regulation of protein kinase B signaling	1	30	19	13332	0.0	0.001	23.38947368	5728	PTEN	0.041927097	0.07176359	biological_process
GO:0043425	bHLH transcription factor binding	1	30	18	12391	0.0	0.001	22.9462963	860	RUNX2	0.042723356	0.090515585	molecular_function
GO:0048018	receptor ligand activity	1	30	18	12391	0.0	0.001	22.9462963	7422	VEGFA	0.042723356	0.090515585	molecular_function
GO:0048661	positive regulation of smooth muscle cell proliferation	1	30	20	13332	0.0	0.002	22.22	231	AKR1B1	0.044086054	0.074080675	biological_process
GO:0035115	embryonic forelimb morphogenesis	1	30	20	13332	0.0	0.002	22.22	860	RUNX2	0.044086054	0.074080675	biological_process
GO:0003091	renal water homeostasis	1	30	20	13332	0.0	0.002	22.22	231	AKR1B1	0.044086054	0.074080675	biological_process
GO:0001890	placenta development	1	30	20	13332	0.0	0.002	22.22	1432	MAPK14	0.044086054	0.074080675	biological_process
GO:0042803	protein homodimerization activity	4	30	559	12391	0.1	0.045	2.955515802	3320;7153;332;862	HSP90AA1;TOP2A;BIRC5;RUNX1T1	0.04433445	0.091305485	molecular_function
GO:0005248	voltage-gated sodium channel activity	1	30	19	12391	0.0	0.002	21.73859649	6331	SCN5A	0.045044402	0.091305485	molecular_function
GO:0071479	cellular response to ionizing radiation	1	30	21	13332	0.0	0.002	21.16190476	1432	MAPK14	0.046240307	0.075628591	biological_process
GO:0071773	cellular response to BMP stimulus	1	30	21	13332	0.0	0.002	21.16190476	860	RUNX2	0.046240307	0.075628591	biological_process

Term_ID	Term_description	ListHit	ListTotal	PopHit	PopTotal	GeneRatio	BgRatio	Enrichment_score	GeneIds	GeneSymbols	p-value	FDR_bh	Category
GO:0009409	response to cold	1	30	21	13332	0.0	0.002	21.16190476	3320	HSP90AA1	0.046240307	0.075628591	biological_process
GO:0002027	regulation of heart rate	1	30	21	13332	0.0	0.002	21.16190476	6331	SCN5A	0.046240307	0.075628591	biological_process
GO:0002931	response to ischemia	1	30	21	13332	0.0	0.002	21.16190476	2697	GJA1	0.046240307	0.075628591	biological_process
GO:0048469	cell maturation	1	30	21	13332	0.0	0.002	21.16190476	860	RUNX2	0.046240307	0.075628591	biological_process
GO:0001205	transcriptional activator activity, RNA polymerase II distal enhancer sequence-specific DNA binding	1	30	20	12391	0.0	0.002	20.65166667	4780	NFE2L2	0.047360007	0.091305485	molecular_function
GO:0030332	cyclin binding	1	30	20	12391	0.0	0.002	20.65166667	1017	CDK2	0.047360007	0.091305485	molecular_function
GO:0046875	ephrin receptor binding	1	30	20	12391	0.0	0.002	20.65166667	5294	PIK3CG	0.047360007	0.091305485	molecular_function
GO:0042110	T cell activation	1	30	22	13332	0.0	0.002	20.2	5294	PIK3CG	0.048389867	0.078102943	biological_process
GO:0048701	embryonic cranial skeleton morphogenesis	1	30	22	13332	0.0	0.002	20.2	860	RUNX2	0.048389867	0.078102943	biological_process
GO:1901215	negative regulation of neuron death	1	30	22	13332	0.0	0.002	20.2	6818	SULT1A3	0.048389867	0.078102943	biological_process
GO:0032993	protein-DNA complex	1	30	25	14857	0.0	0.002	19.80933333	4780	NFE2L2	0.049315656	0.182878891	cellular_component
GO:0016303	1-phosphatidylinositol-3-kinase activity	1	30	21	12391	0.0	0.002	19.66825397	5294	PIK3CG	0.049670184	0.091305485	molecular_function
GO:0008234	cysteine-type peptidase activity	1	30	21	12391	0.0	0.002	19.66825397	840	CASP7	0.049670184	0.091305485	molecular_function
GO:0001784	phosphotyrosine residue binding	1	30	21	12391	0.0	0.002	19.66825397	5921	RASA1	0.049670184	0.091305485	molecular_function
GO:0017025	TBP-class protein binding	1	30	21	12391	0.0	0.002	19.66825397	196	AHR	0.049670184	0.091305485	molecular_function
