# Supplementary material for: Identifying the Mechanism of Polygoni Cuspidati Rhizoma et Radix in Treating Acute Liver Failure Based on Network Pharmacology and Molecular Docking
Source: Gastroenterol Res Pract. 2022 Apr 8;2022:2021066. doi: 10.1155/2022/2021066 (PMC9012611; doi:10.1155/2022/2021066)
Supplement: Supplementary Materials — The 200 target genes interacting with the 10 bioactive components of PCRR, and the 2913 ALF-related target genes are provided in supplementary Tables S1 and S2, respectively. The 153 ALF-related PCRR target candidates are provided in supplementary Table S3. The results of the GO and KEGG pathway enrichment analyses are provided in supplementary Tables S4 and S5, respectively. [file 2021066.f1.zip › 2021066.f5.pdf]

Supplementary file

Table S5. Kyoto Encyclopedia of Genes and Genomes pathway enrichment analysis

Term_ID	Term_description	ListHit	ListTotal	PopHit	PopTotal	GeneRatio	BgRatio	Enrichment_score	GeneIds	GeneSymbols	p-value	FDR_bh	Classification_level1
path:hsa05200	Pathways in cancer	62	145	523	7246	0.42758621	0.07217775	5.924071998	4233;3558;5468;1029;4780;4792;862;1950;5578;5743;2099;841;836;2064;2100;4318;5894;598;207;7157;3565;595;1869;5594;5728;2932;596;840;1147;1956;1728;6256;581;3481;3569;2944;3458;4313;3725;4193;3320;3162;5579;842;11186;5970;7040;4312;3091;332;1019;4843;2353;6772;1026;7422;4609;2950;367;3576;1017;5925	MET;IL2;PPARG;CDKN2A;NFE2L2;NFKBIA;RUNX1T1;EGF;PRKCA;PTGS2;ESR1;CASP8;CASP3;ERBB2;ESR2;MMP9;RAF1;BCL2L1;AKT1;TP53;IL4;CCND1;E2F1;MAPK1;PTEN;GSK3B;BCL2;CASP7;CHUK;EGFR;NQO1;RXRA;BAX;IGF2;IL6;GSTM1;IFNG;MMP2;JUN;MDM2;HSP90AA1;HMOX1;PRKCB;CASP9;RASSF1;RELA;TGFB1;MMP1;HIF1A;BIRC5;CDK4;NOS2;FOS;STAT1;CDKN1A;VEGFA;MYC;GSTP1;AR;CXCL8;CDK2;RB1	1.40E-33	3.43E-31	Human Diseases
path:hsa04933	AGE-RAGE signaling pathway in diabetic complications	31	145	99	7246	0.2137931	0.01366271	15.64792755	6401;5578;5054;836;6347;207;4846;3383;595;1277;5594;596;1281;581;3569;4313;3725;7124;5579;3552;5970;7040;1019;1432;2152;6772;7056;7422;3553;7412;3576	SELE;PRKCA;SERPINE1;CASP3;CCL2;AKT1;NOS3;ICAM1;CCND1;COL1A1;MAPK1;BCL2;COL3A1;BAX;IL6;MMP2;JUN;TNF;PRKCB;IL1A;RELA;TGFB1;CDK4;MAPK14;F3;STAT1;THBD;VEGFA;IL1B;VCAM1;CXCL8	1.17E-29	1.43E-27	Human Diseases
path:hsa05161	Hepatitis B	33	145	143	7246	0.22758621	0.01973503	11.53209549	4792;5578;841;836;4318;5894;207;7157;595;1869;5594;5728;596;1147;581;3569;3725;7124;842;5579;5970;7040;332;1019;2353;6772;1026;4609;890;3576;1017;5111;5925	NFKBIA;PRKCA;CASP8;CASP3;MMP9;RAF1;AKT1;TP53;CCND1;E2F1;MAPK1;PTEN;BCL2;CHUK;BAX;IL6;JUN;TNF;CASP9;PRKCB;RELA;TGFB1;BIRC5;CDK4;FOS;STAT1;CDKN1A;MYC;CCNA2;CXCL8;CDK2;PCNA;RB1	9.43E-27	7.67E-25	Human Diseases
path:hsa05215	Prostate cancer	27	145	97	7246	0.1862069	0.0133867	13.90984714	1950;4792;5327;2064;4318;4314;5894;207;5328;7157;595;1869;5594;5728;2932;596;1147;1956;4193;3320;842;5970;1026;2950;367;1017;5925	EGF;NFKBIA;PLAT;ERBB2;MMP9;MMP3;RAF1;AKT1;PLAU;TP53;CCND1;E2F1;MAPK1;PTEN;GSK3B;BCL2;CHUK;EGFR;MDM2;HSP90AA1;CASP9;RELA;CDKN1A;GSTP1;AR;CDK2;RB1	2.61E-24	1.59E-22	Human Diseases
path:hsa05219	Bladder cancer	20	145	41	7246	0.13793103	0.00565829	24.37678722	1029;1950;2064;4318;5894;7157;595;1869;5594;1956;4313;4193;11186;4312;1019;1026;7422;4609;3576;5925	CDKN2A;EGF;ERBB2;MMP9;RAF1;TP53;CCND1;E2F1;MAPK1;EGFR;MMP2;MDM2;RASSF1;MMP1;CDK4;CDKN1A;VEGFA;MYC;CXCL8;RB1	5.23E-24	2.55E-22	Human Diseases
path:hsa05418	Fluid shear stress and atherosclerosis	30	145	136	7246	0.20689655	0.01876898	11.02332657	6401;653361;4780;5327;6347;4318;207;4846;7157;3383;596;1147;1728;3458;2944;4313;3725;3320;7124;3162;3552;5970;857;2353;1432;7056;7422;3553;2950;7412	SELE;NCF1;NFE2L2;PLAT;CCL2;MMP9;AKT1;NOS3;TP53;ICAM1;BCL2;CHUK;NQO1;IFNG;GSTM1;MMP2;JUN;HSP90AA1;TNF;HMOX1;IL1A;RELA;CAV1;FOS;MAPK14;THBD;VEGFA;IL1B;GSTP1;VCAM1	1.05E-23	4.29E-22	Human Diseases
path:hsa04657	IL-17 signaling pathway	24	145	93	7246	0.16551724	0.01283467	12.89610679	3627;4792;5743;841;836;6347;4318;4314;3565;5594;2932;1147;3458;3569;3725;3320;2920;7124;5970;4312;2353;1432;3553;3576	CXCL10;NFKBIA;PTGS2;CASP8;CASP3;CCL2;MMP9;MMP3;IL4;MAPK1;GSK3B;CHUK;IFNG;IL6;JUN;HSP90AA1;CXCL2;TNF;RELA;MMP1;FOS;MAPK14;IL1B;CXCL8	8.34E-21	2.91E-19	Organismal Systems
path:hsa05167	Kaposi sarcoma-associated herpesvirus infection	30	145	186	7246	0.20689655	0.02566933	8.060066741	5294;4792;5743;841;836;5894;207;7157;3383;595;1869;5594;2932;1147;581;3569;3725;2920;842;5970;3091;1019;1432;2353;6772;1026;7422;4609;3576;5925	PIK3CG;NFKBIA;PTGS2;CASP8;CASP3;RAF1;AKT1;TP53;ICAM1;CCND1;E2F1;MAPK1;GSK3B;CHUK;BAX;IL6;JUN;CXCL2;CASP9;RELA;HIF1A;CDK4;MAPK14;FOS;STAT1;CDKN1A;VEGFA;MYC;CXCL8;RB1	1.54E-19	4.70E-18	Human Diseases

Term_ID	Term_description	ListHit	ListTotal	PopHit	PopTotal	GeneRatio	BgRatio	Enrichment_score	GeneIds	GeneSymbols	p-value	FDR_bh	Classification_level1
path:hsa05212	Pancreatic cancer	21	145	75	7246	0.14482759	0.01035054	13.99227586	1029;1950;2064;5894;598;207;7157;595;1869;5594;1147;1956;581;842;5970;7040;1019;6772;1026;7422;5925	CDKN2A;EGF;ERBB2;RAF1;BCL2L1;AKT1;TP53;CCND1;E2F1;MAPK1;CHUK;EGFR;BAX;CASP9;RELA;TGFB1;CDK4;STAT1;CDKN1A;VEGFA;RB1	4.10E-19	1.11E-17	Human Diseases
path:hsa04668	TNF signaling pathway	24	145	110	7246	0.16551724	0.01518079	10.9030721	3627;6401;4792;5743;841;836;6347;4318;4314;3659;207;3383;5594;840;1147;3569;3725;2920;7124;5970;2353;1432;3553;7412	CXCL10;SELE;NFKBIA;PTGS2;CASP8;CASP3;CCL2;MMP9;MMP3;IRF1;AKT1;ICAM1;MAPK1;CASP7;CHUK;IL6;JUN;CXCL2;TNF;RELA;FOS;MAPK14;IL1B;VCAM1	6.13E-19	1.49E-17	Environmental Information Processing
path:hsa05163	Human cytomegalovirus infection	31	145	223	7246	0.2137931	0.0307756	6.946837792	1029;4792;5743;5578;841;836;6347;5894;207;7157;595;1869;5594;2932;1147;1956;581;3569;4193;7124;842;5579;5970;1019;1432;1026;7422;3553;4609;3576;5925	CDKN2A;NFKBIA;PTGS2;PRKCA;CASP8;CASP3;CCL2;RAF1;AKT1;TP53;CCND1;E2F1;MAPK1;GSK3B;CHUK;EGFR;BAX;IL6;MDM2;TNF;CASP9;PRKCB;RELA;CDK4;MAPK14;CDKN1A;VEGFA;IL1B;MYC;CXCL8;RB1	3.14E-18	6.96E-17	Human Diseases
path:hsa01522	Endocrine resistance	22	145	96	7246	0.15172414	0.01324869	11.45201149	1029;2099;2064;2100;4318;5894;207;7157;595;1869;5594;596;1956;581;4313;3725;4193;1019;1432;2353;1026;5925	CDKN2A;ESR1;ERBB2;ESR2;MMP9;RAF1;AKT1;TP53;CCND1;E2F1;MAPK1;BCL2;EGFR;BAX;MMP2;JUN;MDM2;CDK4;MAPK14;FOS;CDKN1A;RB1	6.45E-18	1.31E-16	Human Diseases
path:hsa05206	MicroRNAs in cancer	26	145	150	7246	0.17931034	0.02070108	8.661885057	4233;1029;5743;5578;836;2064;4318;5894;5328;7157;595;1545;1869;5594;5728;596;1956;2065;4193;3162;5579;11186;4170;1026;7422;4609	MET;CDKN2A;PTGS2;PRKCA;CASP3;ERBB2;MMP9;RAF1;PLAU;TP53;CCND1;CYP1B1;E2F1;MAPK1;PTEN;BCL2;EGFR;ERBB3;MDM2;HMOX1;PRKCB;RASSF1;MCL1;CDKN1A;VEGFA;MYC	8.36E-18	1.57E-16	Human Diseases
path:hsa05223	Non-small cell lung cancer	19	145	66	7246	0.13103448	0.00910847	14.38599791	1029;1950;5578;2064;5894;207;7157;595;1869;5594;1956;6256;581;842;5579;11186;1019;1026;5925	CDKN2A;EGF;PRKCA;ERBB2;RAF1;AKT1;TP53;CCND1;E2F1;MAPK1;EGFR;RXRA;BAX;CASP9;PRKCB;RASSF1;CDK4;CDKN1A;RB1	1.25E-17	2.17E-16	Human Diseases
path:hsa04218	Cellular senescence	26	145	158	7246	0.17931034	0.02180513	8.223308599	11200;1029;5054;5894;207;7157;595;1869;5594;5728;3569;4193;3486;3552;5970;7040;891;1019;1432;1026;4609;1111;890;3576;1017;5925	CHEK2;CDKN2A;SERPINE1;RAF1;AKT1;TP53;CCND1;E2F1;MAPK1;PTEN;IL6;MDM2;IGFBP3;IL1A;RELA;TGFB1;CCNB1;CDK4;MAPK14;CDKN1A;MYC;CHEK1;CCNA2;CXCL8;CDK2;RB1	3.21E-17	5.22E-16	Cellular Processes
path:hsa05222	Small cell lung cancer	21	145	93	7246	0.14482759	0.01283467	11.28409344	4792;5743;836;598;207;7157;595;1869;5728;596;1147;6256;581;842;5970;4843;1019;1026;4609;1017;5925	NFKBIA;PTGS2;CASP3;BCL2L1;AKT1;TP53;CCND1;E2F1;PTEN;BCL2;CHUK;RXRA;BAX;CASP9;RELA;NOS2;CDK4;CDKN1A;MYC;CDK2;RB1	5.35E-17	8.15E-16	Human Diseases
path:hsa04115	p53 signaling pathway	19	145	72	7246	0.13103448	0.00993652	13.18716475	11200;1029;5054;836;841;598;7157;595;5728;596;581;4193;842;3486;891;1019;1026;1111;1017	CHEK2;CDKN2A;SERPINE1;CASP3;CASP8;BCL2L1;TP53;CCND1;PTEN;BCL2;BAX;MDM2;CASP9;IGFBP3;CCNB1;CDK4;CDKN1A;CHEK1;CDK2	7.67E-17	1.10E-15	Cellular Processes
path:hsa05225	Hepatocellular carcinoma	26	145	165	7246	0.17931034	0.02277118	7.874440961	4233;1029;4780;5578;5894;598;207;7157;595;1869;5594;5728;2932;1956;1728;581;3481;2944;3162;5579;7040;1019;1026;4609;2950;5925	MET;CDKN2A;NFE2L2;PRKCA;RAF1;BCL2L1;AKT1;TP53;CCND1;E2F1;MAPK1;PTEN;GSK3B;EGFR;NQO1;BAX;IGF2;GSTM1;HMOX1;PRKCB;TGFB1;CDK4;CDKN1A;MYC;GSTP1;RB1	9.75E-17	1.32E-15	Human Diseases

Term_ID	Term_description	ListHit	ListTotal	PopHit	PopTotal	GeneRatio	BgRatio	Enrichment_score	GeneIds	GeneSymbols	p-value	FDR_bh	Classification_level1
path:hsa04151	PI3K-Akt signaling pathway	36	145	353	7246	0.24827586	0.04871653	5.096336817	4233;5294;3558;1950;5578;3643;2064;5894;598;6696;207;4846;7157;3565;595;1277;5594;5728;2932;596;1147;1956;6256;3481;3569;2065;4193;3320;842;5970;4170;1019;1026;7422;4609;1017	MET;PIK3CG;IL2;EGF;PRKCA;INSR;ERBB2;RAF1;BCL2L1;SPP1;AKT1;NOS3;TP53;IL4;CCND1;COL1A1;MAPK1;PTEN;GSK3B;BCL2;CHUK;EGFR;RXRA;IGF2;IL6;ERBB3;MDM2;HSP90AA1;CASP9;RELA;MCL1;CDK4;CDKN1A;VEGFA;MYC;CDK2	1.22E-16	1.57E-15	Environmental Information Processing
path:hsa05140	Leishmaniasis	18	145	70	7246	0.12413793	0.0096605	12.85004926	3458;4843;2353;1432;3725;6772;3565;653361;3553;4792;5743;3586;7124;5594;5579;3552;5970;7040	IFNG;NOS2;FOS;MAPK14;JUN;STAT1;IL4;NCF1;IL1B;NFKBIA;PTGS2;IL10;TNF;MAPK1;PRKCB;IL1A;RELA;TGFB1	8.70E-16	1.06E-14	Human Diseases
path:hsa04066	HIF-1 signaling pathway	20	145	100	7246	0.13793103	0.01380072	9.994482759	3099;1950;5578;3643;5054;2064;207;4846;5594;596;1956;3458;3569;3162;5579;5970;3091;4843;1026;7422	HK2;EGF;PRKCA;INSR;SERPINE1;ERBB2;AKT1;NOS3;MAPK1;BCL2;EGFR;IFNG;IL6;HMOX1;PRKCB;RELA;HIF1A;NOS2;CDKN1A;VEGFA	3.89E-15	4.52E-14	Environmental Information Processing
path:hsa05220	Chronic myeloid leukemia	18	145	76	7246	0.12413793	0.01048855	11.83557169	581;5894;598;1019;1029;207;7157;4193;1026;595;4792;4609;1147;1869;5594;5970;7040;5925	BAX;RAF1;BCL2L1;CDK4;CDKN2A;AKT1;TP53;MDM2;CDKN1A;CCND1;NFKBIA;MYC;CHUK;E2F1;MAPK1;RELA;TGFB1;RB1	4.25E-15	4.71E-14	Human Diseases
path:hsa05142	Chagas disease (American trypanosomiasis)	20	145	101	7246	0.13793103	0.01393872	9.895527484	3558;4792;841;5054;3586;6347;207;5594;1147;3458;3569;3725;7124;5970;7040;4843;2353;1432;3553;3576	IL2;NFKBIA;CASP8;SERPINE1;IL10;CCL2;AKT1;MAPK1;CHUK;IFNG;IL6;JUN;TNF;RELA;TGFB1;NOS2;FOS;MAPK14;IL1B;CXCL8	4.77E-15	5.06E-14	Human Diseases
path:hsa05169	Epstein-Barr virus infection	26	145	198	7246	0.17931034	0.02732542	6.562034134	3627;4792;841;836;207;7157;3383;595;1869;596;1147;581;3569;3725;4193;7124;842;5970;1019;1432;6772;1026;4609;890;1017;5925	CXCL10;NFKBIA;CASP8;CASP3;AKT1;TP53;ICAM1;CCND1;E2F1;BCL2;CHUK;BAX;IL6;JUN;MDM2;TNF;CASP9;RELA;CDK4;MAPK14;STAT1;CDKN1A;MYC;CCNA2;CDK2;RB1	9.38E-15	9.15E-14	Human Diseases
path:hsa05205	Proteoglycans in cancer	26	145	198	7246	0.17931034	0.02732542	6.562034134	4233;5578;2099;836;2064;4318;5894;207;5328;7157;595;5594;1956;3481;4313;2065;4193;7124;5579;7040;857;3091;1432;1026;7422;4609	MET;PRKCA;ESR1;CASP3;ERBB2;MMP9;RAF1;AKT1;PLAU;TP53;CCND1;MAPK1;EGFR;IGF2;MMP2;ERBB3;MDM2;TNF;PRKCB;TGFB1;CAV1;HIF1A;MAPK14;CDKN1A;VEGFA;MYC	9.38E-15	9.15E-14	Human Diseases
path:hsa05214	Glioma	17	145	71	7246	0.11724138	0.00979851	11.96522584	581;5894;1019;1029;207;7157;4193;1026;595;1950;1869;5594;5578;5728;5579;1956;5925	BAX;RAF1;CDK4;CDKN2A;AKT1;TP53;MDM2;CDKN1A;CCND1;EGF;E2F1;MAPK1;PRKCA;PTEN;PRKCB;EGFR;RB1	2.11E-14	1.91E-13	Human Diseases
path:hsa01524	Platinum drug resistance	17	145	71	7246	0.11724138	0.00979851	11.96522584	581;7153;598;332;2944;1029;207;7157;4193;1026;2064;2950;842;5594;836;596;841	BAX;TOP2A;BCL2L1;BIRC5;GSTM1;CDKN2A;AKT1;TP53;MDM2;CDKN1A;ERBB2;GSTP1;CASP9;MAPK1;CASP3;BCL2;CASP8	2.11E-14	1.91E-13	Human Diseases
path:hsa05145	Toxoplasmosis	20	145	111	7246	0.13793103	0.0153188	9.004038521	5294;4792;841;3586;836;959;598;207;5594;596;1147;3458;7124;842;240;5970;7040;4843;1432;6772	PIK3CG;NFKBIA;CASP8;IL10;CASP3;CD40LG;BCL2L1;AKT1;MAPK1;BCL2;CHUK;IFNG;TNF;CASP9;ALOX5;RELA;TGFB1;NOS2;MAPK14;STAT1	3.24E-14	2.82E-13	Human Diseases
path:hsa05210	Colorectal cancer	18	145	86	7246	0.12413793	0.01186862	10.45934242	581;5894;332;207;2353;3725;7157;1026;595;4609;1950;842;5594;2932;596;836;7040;1956	BAX;RAF1;BIRC5;AKT1;FOS;JUN;TP53;CDKN1A;CCND1;MYC;EGF;CASP9;MAPK1;GSK3B;BCL2;CASP3;TGFB1;EGFR	4.37E-14	3.68E-13	Human Diseases
path:hsa01521	EGFR tyrosine kinase inhibitor resistance	17	145	79	7246	0.11724138	0.01090257	10.7535574	581;4233;5894;598;3569;207;2065;7422;1950;5578;5594;5579;5728;2932;596;2064;1956	BAX;MET;RAF1;BCL2L1;IL6;AKT1;ERBB3;VEGFA;EGF;PRKCA;MAPK1;PRKCB;PTEN;GSK3B;BCL2;ERBB2;EGFR	1.42E-13	1.16E-12	Human Diseases

Term_ID	Term_description	ListHit	ListTotal	PopHit	PopTotal	GeneRatio	BgRatio	Enrichment_score	GeneIds	GeneSymbols	p-value	FDR_bh	Classification_level1
path:hsa04210	Apoptosis	21	145	135	7246	0.14482759	0.01863097	7.77348659	4792;841;836;5894;598;207;7157;5594;596;840;1147;581;3725;142;7124;842;5970;4170;332;2353;1509	NFKBIA;CASP8;CASP3;RAF1;BCL2L1;AKT1;TP53;MAPK1;BCL2;CASP7;CHUK;BAX;JUN;PARP1;TNF;CASP9;RELA;MCL1;BIRC5;FOS;CTSD	1.49E-13	1.17E-12	Cellular Processes
path:hsa05218	Melanoma	16	145	72	7246	0.11034483	0.00993652	11.10498084	581;4233;5894;1019;1029;207;7157;4193;1026;595;1950;1869;5594;5728;1956;5925	BAX;MET;RAF1;CDK4;CDKN2A;AKT1;TP53;MDM2;CDKN1A;CCND1;EGF;E2F1;MAPK1;PTEN;EGFR;RB1	4.53E-13	3.46E-12	Human Diseases
path:hsa05224	Breast cancer	21	145	147	7246	0.14482759	0.02028705	7.138916256	5241;1950;2099;2064;2100;5894;207;7157;595;1869;5594;5728;2932;1956;581;3725;1019;2353;1026;4609;5925	PGR;EGF;ESR1;ERBB2;ESR2;RAF1;AKT1;TP53;CCND1;E2F1;MAPK1;PTEN;GSK3B;EGFR;BAX;JUN;CDK4;FOS;CDKN1A;MYC;RB1	8.37E-13	6.19E-12	Human Diseases
path:hsa04068	FoxO signaling pathway	20	145	132	7246	0.13793103	0.01821695	7.571577847	1950;3643;3586;847;5894;207;595;5594;5728;1147;1956;3569;4193;6517;7040;891;6648;1432;1026;1017	EGF;INSR;IL10;CAT;RAF1;AKT1;CCND1;MAPK1;PTEN;CHUK;EGFR;IL6;MDM2;SLC2A4;TGFB1;CCNB1;SOD2;MAPK14;CDKN1A;CDK2	9.89E-13	7.10E-12	Environmental Information Processing
path:hsa04625	C-type lectin receptor signaling pathway	18	145	104	7246	0.12413793	0.01435275	8.649071618	5894;3558;3659;3569;841;3725;207;4193;1432;6772;3553;4792;5743;3586;7124;5594;5970;1147	RAF1;IL2;IRF1;IL6;CASP8;JUN;AKT1;MDM2;MAPK14;STAT1;IL1B;NFKBIA;PTGS2;IL10;TNF;MAPK1;RELA;CHUK	1.40E-12	9.78E-12	Organismal Systems
path:hsa05164	Influenza A	22	145	169	7246	0.15172414	0.02332321	6.505284636	3627;4792;5578;6347;5894;207;3383;5594;2932;3458;3569;3725;5644;7124;842;5579;3552;5970;1432;6772;3553;3576	CXCL10;NFKBIA;PRKCA;CCL2;RAF1;AKT1;ICAM1;MAPK1;GSK3B;IFNG;IL6;JUN;PRSS1;TNF;CASP9;PRKCB;IL1A;RELA;MAPK14;STAT1;IL1B;CXCL8	1.58E-12	1.07E-11	Human Diseases
path:hsa04659	Th17 cell differentiation	18	145	105	7246	0.12413793	0.01449075	8.566699507	3091;3558;3569;3458;1432;3725;6772;3320;2353;3565;3553;4792;1147;196;5594;5970;7040;6256	HIF1A;IL2;IL6;IFNG;MAPK14;JUN;STAT1;HSP90AA1;FOS;IL4;IL1B;NFKBIA;CHUK;AHR;MAPK1;RELA;TGFB1;RXRA	1.67E-12	1.10E-11	Organismal Systems
path:hsa05166	Human T-cell leukemia virus 1 infection	26	145	253	7246	0.17931034	0.03491582	5.135504975	3558;11200;1029;4792;598;207;7157;3383;595;1869;2932;1147;581;3569;3725;7124;5970;7040;1019;2353;1026;4609;1111;7412;5111;5925	IL2;CHEK2;CDKN2A;NFKBIA;BCL2L1;AKT1;TP53;ICAM1;CCND1;E2F1;GSK3B;CHUK;BAX;IL6;JUN;TNF;RELA;TGFB1;CDK4;FOS;CDKN1A;MYC;CHEK1;VCAM1;PCNA;RB1	3.29E-12	2.11E-11	Human Diseases
path:hsa05213	Endometrial cancer	14	145	58	7246	0.09655172	0.00800442	12.06230678	581;5894;207;7157;1026;595;4609;1950;842;5594;5728;2932;2064;1956	BAX;RAF1;AKT1;TP53;CDKN1A;CCND1;MYC;EGF;CASP9;MAPK1;PTEN;GSK3B;ERBB2;EGFR	4.19E-12	2.62E-11	Human Diseases
path:hsa05144	Malaria	13	145	49	7246	0.08965517	0.00676235	13.25798733	4233;6347;959;3458;3569;6401;3383;3553;7124;7412;3586;7040;3576	MET;CCL2;CD40LG;IFNG;IL6;SELE;ICAM1;IL1B;TNF;VCAM1;IL10;TGFB1;CXCL8	6.80E-12	4.15E-11	Human Diseases
path:hsa04926	Relaxin signaling pathway	19	145	130	7246	0.13103448	0.01794093	7.303660477	4792;5578;4318;5894;207;4846;1277;5594;1956;1281;4313;3725;5970;7040;4312;4843;2353;1432;7422	NFKBIA;PRKCA;MMP9;RAF1;AKT1;NOS3;COL1A1;MAPK1;EGFR;COL3A1;MMP2;JUN;RELA;TGFB1;MMP1;NOS2;FOS;MAPK14;VEGFA	7.40E-12	4.40E-11	Organismal Systems
path:hsa04660	T cell receptor signaling pathway	17	145	101	7246	0.11724138	0.01393872	8.411198361	959;5894;3558;3458;1019;207;3725;1432;2353;3565;4792;7124;3586;5594;2932;5970;1147	CD40LG;RAF1;IL2;IFNG;CDK4;AKT1;JUN;MAPK14;FOS;IL4;NFKBIA;TNF;IL10;MAPK1;GSK3B;RELA;CHUK	9.81E-12	5.70E-11	Organismal Systems
path:hsa05133	Pertussis	15	145	76	7246	0.10344828	0.01048855	9.862976407	3569;3659;1432;3725;4843;2353;840;3553;7124;3586;3552;5594;836;5970;3576	IL6;IRF1;MAPK14;JUN;NOS2;FOS;CASP7;IL1B;TNF;IL10;IL1A;MAPK1;CASP3;RELA;CXCL8	1.59E-11	9.04E-11	Human Diseases

Term_ID	Term_description	ListHit	ListTotal	PopHit	PopTotal	GeneRatio	BgRatio	Enrichment_score	GeneIds	GeneSymbols	p-value	FDR_bh	Classification_level1
path:hsa04010	MAPK signaling pathway	27	145	295	7246	0.1862069	0.04071212	4.573746347	4233;1950;5578;3643;836;2064;5894;207;7157;5594;1147;1956;3481;3725;2065;7124;5579;3552;5970;7040;2353;1432;7422;3553;4609;3315;5921	MET;EGF;PRKCA;INSR;CASP3;ERBB2;RAF1;AKT1;TP53;MAPK1;CHUK;EGFR;IGF2;JUN;ERBB3;TNF;PRKCB;IL1A;RELA;TGFB1;FOS;MAPK14;VEGFA;IL1B;MYC;HSPB1;RASA1	1.87E-11	1.04E-10	Environmental Information Processing
path:hsa04064	NF-kappa B signaling pathway	16	145	93	7246	0.11034483	0.01283467	8.597404524	959;598;5328;142;3383;2920;3553;4792;5743;7124;5579;7412;596;5970;1147;3576	CD40LG;BCL2L1;PLAU;PARP1;ICAM1;CXCL2;IL1B;NFKBIA;PTGS2;TNF;PRKCB;VCAM1;BCL2;RELA;CHUK;CXCL8	2.93E-11	1.59E-10	Environmental Information Processing
path:hsa05226	Gastric cancer	19	145	148	7246	0.13103448	0.02042506	6.415377446	4233;1950;2064;5894;207;7157;595;1869;5594;2932;596;1956;6256;581;7040;1026;4609;1017;5925	MET;EGF;ERBB2;RAF1;AKT1;TP53;CCND1;E2F1;MAPK1;GSK3B;BCL2;EGFR;RXRA;BAX;TGFB1;CDKN1A;MYC;CDK2;RB1	7.65E-11	4.06E-10	Human Diseases
path:hsa05160	Hepatitis C	18	145	131	7246	0.12413793	0.01807894	6.866438536	5894;3659;207;1432;7157;6772;1026;1950;4792;5465;7124;5594;2932;5970;1147;3576;1956;6256	RAF1;IRF1;AKT1;MAPK14;TP53;STAT1;CDKN1A;EGF;NFKBIA;PPARA;TNF;MAPK1;GSK3B;RELA;CHUK;CXCL8;EGFR;RXRA	7.94E-11	4.12E-10	Human Diseases
path:hsa05165	Human papillomavirus infection	28	145	339	7246	0.19310345	0.04678443	4.127515004	1950;5743;841;836;5894;3659;6696;207;7157;595;1277;1869;5594;5728;2932;1147;1956;581;4193;7124;5970;1019;6772;1026;7422;890;1017;5925	EGF;PTGS2;CASP8;CASP3;RAF1;IRF1;SPP1;AKT1;TP53;CCND1;COL1A1;E2F1;MAPK1;PTEN;GSK3B;CHUK;EGFR;BAX;MDM2;TNF;RELA;CDK4;STAT1;CDKN1A;VEGFA;CCNA2;CDK2;RB1	8.74E-11	4.44E-10	Human Diseases
path:hsa04620	Toll-like receptor signaling pathway	16	145	102	7246	0.11034483	0.01407673	7.838810007	3627;3569;6696;841;3725;207;1432;6772;2353;3553;4792;7124;5594;5970;1147;3576	CXCL10;IL6;SPP1;CASP8;JUN;AKT1;MAPK14;STAT1;FOS;IL1B;NFKBIA;TNF;MAPK1;RELA;CHUK;CXCL8	1.25E-10	6.24E-10	Organismal Systems
path:hsa05152	Tuberculosis	20	145	175	7246	0.13793103	0.02415126	5.711133005	841;3586;836;5894;207;5594;596;581;3458;3569;7124;842;3552;5970;7040;4843;1432;6772;1509;3553	CASP8;IL10;CASP3;RAF1;AKT1;MAPK1;BCL2;BAX;IFNG;IL6;TNF;CASP9;IL1A;RELA;TGFB1;NOS2;MAPK14;STAT1;CTSD;IL1B	1.99E-10	9.71E-10	Human Diseases
path:hsa04110	Cell cycle	17	145	124	7246	0.11724138	0.01711289	6.85105673	11200;1019;1029;7157;4193;1026;595;4609;1111;1869;2932;890;7040;891;1017;5111;5925	CHEK2;CDK4;CDKN2A;TP53;MDM2;CDKN1A;CCND1;MYC;CHEK1;E2F1;GSK3B;CCNA2;TGFB1;CCNB1;CDK2;PCNA;RB1	2.87E-10	1.37E-09	Cellular Processes
path:hsa05146	Amoebiasis	15	145	95	7246	0.10344828	0.01311068	7.890381125	3458;3569;4843;3553;1277;7124;3586;5578;5579;836;3315;5970;7040;3576;1281	IFNG;IL6;NOS2;IL1B;COL1A1;TNF;IL10;PRKCA;PRKCB;CASP3;HSPB1;RELA;TGFB1;CXCL8;COL3A1	4.47E-10	2.10E-09	Human Diseases
path:hsa05202	Transcriptional misregulation in cancer	20	145	185	7246	0.13793103	0.02553133	5.402423113	4233;5468;862;5327;860;4318;4314;598;5328;7157;6256;581;3569;4193;3486;4353;5970;1026;4609;3576	MET;PPARG;RUNX1T1;PLAT;RUNX2;MMP9;MMP3;BCL2L1;PLAU;TP53;RXRA;BAX;IL6;MDM2;IGFBP3;MPO;RELA;CDKN1A;MYC;CXCL8	5.44E-10	2.51E-09	Human Diseases
path:hsa05143	African trypanosomiasis	10	145	34	7246	0.06896552	0.00469224	14.69776876	3458;3569;6401;3383;3553;7124;5578;7412;5579;3586	IFNG;IL6;SELE;ICAM1;IL1B;TNF;PRKCA;VCAM1;PRKCB;IL10	6.56E-10	2.96E-09	Human Diseases
path:hsa04919	Thyroid hormone signaling pathway	16	145	116	7246	0.11034483	0.01600883	6.89274673	3091;5894;2099;207;7157;6772;4193;595;4609;842;5578;5594;5579;2932;10499;6256	HIF1A;RAF1;ESR1;AKT1;TP53;STAT1;MDM2;CCND1;MYC;CASP9;PRKCA;MAPK1;PRKCB;GSK3B;NCOA2;RXRA	9.05E-10	4.01E-09	Organismal Systems
path:hsa05323	Rheumatoid arthritis	14	145	86	7246	0.09655172	0.01186862	8.135044106	6347;4314;3458;3569;2353;3725;3383;7422;3553;7124;3552;7040;3576;4312	CCL2;MMP3;IFNG;IL6;FOS;JUN;ICAM1;VEGFA;IL1B;TNF;IL1A;TGFB1;CXCL8;MMP1	1.16E-09	5.06E-09	Human Diseases
path:hsa04915	Estrogen signaling pathway	17	145	136	7246	0.11724138	0.01876898	6.246551724	2100;4318;5894;207;4846;4313;3725;1509;10499;3320;2353;5241;4988;5594;2099;596;1956	ESR2;MMP9;RAF1;AKT1;NOS3;MMP2;JUN;CTSD;NCOA2;HSP90AA1;FOS;PGR;OPRM1;MAPK1;ESR1;BCL2;EGFR	1.25E-09	5.35E-09	Organismal Systems

Term_ID	Term_description	ListHit	ListTotal	PopHit	PopTotal	GeneRatio	BgRatio	Enrichment_score	GeneIds	GeneSymbols	p-value	FDR_bh	Classification_level1
path:hsa04380	Osteoclast differentiation	16	145	125	7246	0.11034483	0.0172509	6.396468966	3458;5468;207;1432;3725;6772;2353;653361;3553;4792;1147;7124;3552;5594;5970;7040	IFNG;PPARG;AKT1;MAPK14;JUN;STAT1;FOS;NCF1;IL1B;NFKBIA;CHUK;TNF;IL1A;MAPK1;RELA;TGFB1	2.78E-09	1.17E-08	Organismal Systems
path:hsa05321	Inflammatory bowel disease (IBD)	12	145	63	7246	0.08275862	0.00869445	9.518555008	3558;3569;3458;3725;6772;3565;3553;7124;3586;3552;5970;7040	IL2;IL6;IFNG;JUN;STAT1;IL4;IL1B;TNF;IL10;IL1A;RELA;TGFB1	2.90E-09	1.20E-08	Human Diseases
path:hsa04932	Non-alcoholic fatty liver disease (NAFLD)	17	145	145	7246	0.11724138	0.02001104	5.858834721	581;3569;841;207;3725;840;3553;7124;3552;3643;836;2932;5970;7040;3576;5465;6256	BAX;IL6;CASP8;AKT1;JUN;CASP7;IL1B;TNF;IL1A;INSR;CASP3;GSK3B;RELA;TGFB1;CXCL8;PPARA;RXRA	3.41E-09	1.39E-08	Human Diseases
path:hsa05170	Human immunodeficiency virus 1 infection	20	145	211	7246	0.13793103	0.02911951	4.736721687	4792;5578;841;836;5894;598;207;5594;596;1147;581;3725;7124;842;5579;5970;891;2353;1432;1111	NFKBIA;PRKCA;CASP8;CASP3;RAF1;BCL2L1;AKT1;MAPK1;BCL2;CHUK;BAX;JUN;TNF;CASP9;PRKCB;RELA;CCNB1;FOS;MAPK14;CHEK1	5.58E-09	2.20E-08	Human Diseases
path:hsa05162	Measles	16	145	131	7246	0.11034483	0.01807894	6.103500921	3558;3569;1019;207;7157;6772;3458;3565;595;4792;3553;3552;2932;5970;1147;1017	IL2;IL6;CDK4;AKT1;TP53;STAT1;IFNG;IL4;CCND1;NFKBIA;IL1B;IL1A;GSK3B;RELA;CHUK;CDK2	5.58E-09	2.20E-08	Human Diseases
path:hsa05134	Legionellosis	11	145	55	7246	0.07586207	0.00759039	9.994482759	3569;841;840;2920;3553;4792;7124;842;836;5970;3576	IL6;CASP8;CASP7;CXCL2;IL1B;NFKBIA;TNF;CASP9;CASP3;RELA;CXCL8	7.93E-09	3.07E-08	Human Diseases
path:hsa04917	Prolactin signaling pathway	12	145	70	7246	0.08275862	0.0096605	8.566699507	2100;5894;3659;207;1432;2353;2932;6772;595;5594;2099;5970	ESR2;RAF1;IRF1;AKT1;MAPK14;FOS;GSK3B;STAT1;CCND1;MAPK1;ESR1;RELA	1.03E-08	3.88E-08	Organismal Systems
path:hsa04012	ErbB signaling pathway	13	145	85	7246	0.08965517	0.01173061	7.642839757	5894;207;3725;2065;1026;1950;4609;5578;5594;5579;2932;2064;1956	RAF1;AKT1;JUN;ERBB3;CDKN1A;EGF;MYC;PRKCA;MAPK1;PRKCB;GSK3B;ERBB2;EGFR	1.03E-08	3.88E-08	Environmental Information Processing
path:hsa04370	VEGF signaling pathway	11	145	59	7246	0.07586207	0.00814242	9.316890707	5894;207;1432;4846;7422;5743;842;5578;5594;5579;3315	RAF1;AKT1;MAPK14;NOS3;VEGFA;PTGS2;CASP9;PRKCA;MAPK1;PRKCB;HSPB1	1.73E-08	6.40E-08	Environmental Information Processing
path:hsa04621	NOD-like receptor signaling pathway	17	145	167	7246	0.11724138	0.0230472	5.087012183	6347;598;3569;841;1432;3725;6772;3320;2920;3553;4792;7124;5594;596;5970;1147;3576	CCL2;BCL2L1;IL6;CASP8;MAPK14;JUN;STAT1;HSP90AA1;CXCL2;IL1B;NFKBIA;TNF;MAPK1;BCL2;RELA;CHUK;CXCL8	2.95E-08	1.08E-07	Organismal Systems
path:hsa05014	Amyotrophic lateral sclerosis (ALS)	10	145	50	7246	0.06896552	0.00690036	9.994482759	581;598;1432;7157;6647;7124;842;596;836;847	BAX;BCL2L1;MAPK14;TP53;SOD1;TNF;CASP9;BCL2;CASP3;CAT	3.91E-08	1.40E-07	Human Diseases
path:hsa05230	Central carbon metabolism in cancer	11	145	65	7246	0.07586207	0.00897047	8.456870027	4233;3091;5894;3099;207;7157;4609;5594;5728;2064;1956	MET;HIF1A;RAF1;HK2;AKT1;TP53;MYC;MAPK1;PTEN;ERBB2;EGFR	5.00E-08	1.77E-07	Human Diseases
path:hsa04510	Focal adhesion	18	145	199	7246	0.12413793	0.02746343	4.520117831	4233;5894;6696;207;3725;7422;595;1950;1277;5578;5594;5579;5728;2932;596;2064;1956;857	MET;RAF1;SPP1;AKT1;JUN;VEGFA;CCND1;EGF;COL1A1;PRKCA;MAPK1;PRKCB;PTEN;GSK3B;BCL2;ERBB2;EGFR;CAV1	7.15E-08	2.49E-07	Cellular Processes
path:hsa04071	Sphingolipid signaling pathway	14	145	118	7246	0.09655172	0.01628485	5.92893045	581;5894;207;1432;4846;7157;1509;7124;5578;5594;5579;5728;596;5970	BAX;RAF1;AKT1;MAPK14;NOS3;TP53;CTSD;TNF;PRKCA;MAPK1;PRKCB;PTEN;BCL2;RELA	7.71E-08	2.65E-07	Environmental Information Processing
path:hsa05132	Salmonella infection	12	145	84	7246	0.08275862	0.0115926	7.138916256	3458;3569;4843;1432;3725;2353;2920;3553;3552;5594;5970;3576	IFNG;IL6;NOS2;MAPK14;JUN;FOS;CXCL2;IL1B;IL1A;MAPK1;RELA;CXCL8	8.58E-08	2.91E-07	Human Diseases
path:hsa04630	Jak-STAT signaling pathway	16	145	162	7246	0.11034483	0.02235716	4.935547041	4170;5894;3558;3569;207;598;3458;6772;1026;3565;595;4609;1950;596;3586;1956	MCL1;RAF1;IL2;IL6;AKT1;BCL2L1;IFNG;STAT1;CDKN1A;IL4;CCND1;MYC;EGF;BCL2;IL10;EGFR	1.19E-07	3.97E-07	Environmental Information Processing

Term_ID	Term_description	ListHit	ListTotal	PopHit	PopTotal	GeneRatio	BgRatio	Enrichment_score	GeneIds	GeneSymbols	p-value	FDR_bh	Classification_level1
path:hsa04931	Insulin resistance	13	145	107	7246	0.08965517	0.01476677	6.07141476	3569;207;4846;5770;4792;7124;6517;5579;3643;5728;2932;5970;5465	IL6;AKT1;NOS3;PTPN1;NFKBIA;TNF;SLC2A4;PRKCB;INSR;PTEN;GSK3B;RELA;PPARA	1.74E-07	5.73E-07	Human Diseases
path:hsa04215	Apoptosis - multiple species	8	145	33	7246	0.05517241	0.00455424	12.11452456	581;598;332;840;842;836;596;841	BAX;BCL2L1;BIRC5;CASP7;CASP9;CASP3;BCL2;CASP8	1.93E-07	6.27E-07	Cellular Processes
path:hsa05203	Viral carcinogenesis	17	145	200	7246	0.11724138	0.02760144	4.247655172	581;1019;1029;3725;841;4193;1026;7157;595;4792;1111;5594;836;890;5970;1017;5925	BAX;CDK4;CDKN2A;JUN;CASP8;MDM2;CDKN1A;TP53;CCND1;NFKBIA;CHEK1;MAPK1;CASP3;CCNA2;RELA;CDK2;RB1	4.16E-07	1.34E-06	Human Diseases
path:hsa05216	Thyroid cancer	8	145	37	7246	0.05517241	0.00510627	10.80484623	581;5468;7157;1026;595;4609;5594;6256	BAX;PPARG;TP53;CDKN1A;CCND1;MYC;MAPK1;RXRA	5.01E-07	1.59E-06	Human Diseases
path:hsa04662	B cell receptor signaling pathway	10	145	70	7246	0.06896552	0.0096605	7.138916256	5894;207;2353;3725;4792;5579;5594;2932;5970;1147	RAF1;AKT1;FOS;JUN;NFKBIA;PRKCB;MAPK1;GSK3B;RELA;CHUK	1.07E-06	3.36E-06	Organismal Systems
path:hsa04658	Th1 and Th2 cell differentiation	11	145	90	7246	0.07586207	0.01242065	6.107739464	3558;3458;2353;1432;3725;6772;3565;4792;5594;5970;1147	IL2;IFNG;FOS;MAPK14;JUN;STAT1;IL4;NFKBIA;MAPK1;RELA;CHUK	1.51E-06	4.68E-06	Organismal Systems
path:hsa04726	Serotonergic synapse	12	145	113	7246	0.08275862	0.01559481	5.306805005	351;3356;5894;5742;5743;5578;5594;240;5579;4129;836;6532	APP;HTR2A;RAF1;PTGS1;PTGS2;PRKCA;MAPK1;ALOX5;PRKCB;MAOB;CASP3;SLC6A4	2.28E-06	6.96E-06	Organismal Systems
path:hsa05168	Herpes simplex infection	15	145	183	7246	0.10344828	0.02525531	4.096099491	6347;3458;3569;841;2353;3725;6772;7157;3553;4792;7124;836;5970;1147;1017	CCL2;IFNG;IL6;CASP8;FOS;JUN;STAT1;TP53;IL1B;NFKBIA;TNF;CASP3;RELA;CHUK;CDK2	3.30E-06	9.93E-06	Human Diseases
path:hsa04062	Chemokine signaling pathway	15	145	187	7246	0.10344828	0.02580734	4.00848239	6347;5894;5294;3627;207;6772;2920;653361;4792;5579;5594;2932;5970;1147;3576	CCL2;RAF1;PIK3CG;CXCL10;AKT1;STAT1;CXCL2;NCF1;NFKBIA;PRKCB;MAPK1;GSK3B;RELA;CHUK;CXCL8	4.31E-06	1.28E-05	Organismal Systems
path:hsa05221	Acute myeloid leukemia	9	145	66	7246	0.06206897	0.00910847	6.814420063	5894;207;595;862;4609;4353;5594;5970;1147	RAF1;AKT1;CCND1;RUNX1T1;MYC;MPO;MAPK1;RELA;CHUK	5.63E-06	1.65E-05	Human Diseases
path:hsa05120	Epithelial cell signaling in Helicobacter pylori infection	9	145	68	7246	0.06206897	0.00938449	6.613995943	4233;1432;3725;4792;836;5970;1147;3576;1956	MET;MAPK14;JUN;NFKBIA;CASP3;RELA;CHUK;CXCL8;EGFR	7.24E-06	2.10E-05	Human Diseases
path:hsa05211	Renal cell carcinoma	9	145	69	7246	0.06206897	0.0095225	6.51814093	4233;3091;5894;207;3725;1026;7422;5594;7040	MET;HIF1A;RAF1;AKT1;JUN;CDKN1A;VEGFA;MAPK1;TGFB1	8.19E-06	2.35E-05	Human Diseases
path:hsa04014	Ras signaling pathway	16	145	232	7246	0.11034483	0.03201766	3.446373365	4233;5894;3481;598;207;7422;1950;5578;3643;5594;11186;5579;5970;1147;1956;5921	MET;RAF1;IGF2;BCL2L1;AKT1;VEGFA;EGF;PRKCA;INSR;MAPK1;RASSF1;PRKCB;RELA;CHUK;EGFR;RASA1	1.42E-05	4.03E-05	Environmental Information Processing
path:hsa04722	Neurotrophin signaling pathway	11	145	119	7246	0.07586207	0.01642285	4.619298754	581;5894;207;1432;3725;7157;4792;5594;2932;596;5970	BAX;RAF1;AKT1;MAPK14;JUN;TP53;NFKBIA;MAPK1;GSK3B;BCL2;RELA	2.34E-05	6.55E-05	Organismal Systems
path:hsa05231	Choline metabolism in cancer	10	145	99	7246	0.06896552	0.01366271	5.047718565	3091;5894;207;2353;3725;1950;5578;5594;5579;1956	HIF1A;RAF1;AKT1;FOS;JUN;EGF;PRKCA;MAPK1;PRKCB;EGFR	2.57E-05	7.12E-05	Human Diseases
path:hsa05204	Chemical carcinogenesis	9	145	80	7246	0.06206897	0.01104057	5.621896552	2944;1543;1576;1545;6817;1544;2950;5743;6818	GSTM1;CYP1A1;CYP3A4;CYP1B1;SULT1A1;CYP1A2;GSTP1;PTGS2;SULT1A3	2.78E-05	7.62E-05	Human Diseases
path:hsa01523	Antifolate resistance	6	145	31	7246	0.04137931	0.00427822	9.672080089	3569;3553;7124;5970;1147;9429	IL6;IL1B;TNF;RELA;CHUK;ABCG2	2.82E-05	7.65E-05	Human Diseases
path:hsa04928	Parathyroid hormone synthesis, secretion and action	10	145	106	7246	0.06896552	0.01462876	4.71437866	860;5894;2353;1026;5578;5594;5579;596;1956;6256	RUNX2;RAF1;FOS;CDKN1A;PRKCA;MAPK1;PRKCB;BCL2;EGFR;RXRA	4.66E-05	0.00012501	Organismal Systems

Term_ID	Term_description	ListHit	ListTotal	PopHit	PopTotal	GeneRatio	BgRatio	Enrichment_score	GeneIds	GeneSymbols	p-value	FDR_bh	Classification_level1
path:hsa04921	Oxytocin signaling pathway	12	145	152	7246	0.08275862	0.02097709	3.945190563	5294;5894;2353;4846;3725;1026;595;5743;5578;5594;5579;1956	PIK3CG;RAF1;FOS;NOS3;JUN;CDKN1A;CCND1;PTGS2;PRKCA;MAPK1;PRKCB;EGFR	4.82E-05	0.00012775	Organismal Systems
path:hsa05020	Prion diseases	6	145	34	7246	0.04137931	0.00469224	8.818661258	581;3569;6647;3553;3552;5594	BAX;IL6;SOD1;IL1B;IL1A;MAPK1	4.90E-05	0.00012867	Human Diseases
path:hsa04664	Fc epsilon RI signaling pathway	8	145	67	7246	0.05517241	0.00924648	5.966855378	5894;207;1432;3565;7124;5578;5594;240	RAF1;AKT1;MAPK14;IL4;TNF;PRKCA;MAPK1;ALOX5	5.10E-05	0.00013234	Organismal Systems
path:hsa05330	Allograft rejection	6	145	35	7246	0.04137931	0.00483025	8.566699507	959;3558;3458;3565;7124;3586	CD40LG;IL2;IFNG;IL4;TNF;IL10	5.82E-05	0.00014954	Human Diseases
path:hsa04920	Adipocytokine signaling pathway	8	145	69	7246	0.05517241	0.0095225	5.793903048	207;4792;7124;6517;5970;1147;5465;6256	AKT1;NFKBIA;TNF;SLC2A4;RELA;CHUK;PPARA;RXRA	6.32E-05	0.00016062	Organismal Systems
path:hsa04622	RIG-I-like receptor signaling pathway	8	145	70	7246	0.05517241	0.0096605	5.711133005	3627;841;1432;4792;7124;5970;1147;3576	CXCL10;CASP8;MAPK14;NFKBIA;TNF;RELA;CHUK;CXCL8	7.02E-05	0.00017647	Organismal Systems
path:hsa05332	Graft-versus-host disease	6	145	37	7246	0.04137931	0.00510627	8.103634669	3458;3558;3569;3553;7124;3552	IFNG;IL2;IL6;IL1B;TNF;IL1A	8.07E-05	0.0002009	Human Diseases
path:hsa05416	Viral myocarditis	7	145	56	7246	0.04827586	0.0077284	6.246551724	959;3383;595;842;836;841;857	CD40LG;ICAM1;CCND1;CASP9;CASP3;CASP8;CAV1	0.00011352	0.00027978	Human Diseases
path:hsa04914	Progesterone-mediated oocyte maturation	9	145	99	7246	0.06206897	0.01366271	4.542946708	5894;207;1432;3320;5241;5594;890;891;1017	RAF1;AKT1;MAPK14;HSP90AA1;PGR;MAPK1;CCNA2;CCNB1;CDK2	0.00015036	0.00036687	Organismal Systems
path:hsa04610	Complement and coagulation cascades	8	145	79	7246	0.05517241	0.01090257	5.060497599	2155;2152;5328;7056;2147;2159;5327;5054	F7;F3;PLAU;THBD;F2;F10;PLAT;SERPINE1	0.00016661	0.00040251	Organismal Systems
path:hsa04934	Cushing syndrome	11	145	154	7246	0.07586207	0.02125311	3.569458128	1019;1029;1026;595;1869;196;5594;2932;1956;1017;5925	CDK4;CDKN2A;CDKN1A;CCND1;E2F1;AHR;MAPK1;GSK3B;EGFR;CDK2;RB1	0.00024468	0.00058532	Human Diseases
path:hsa04672	Intestinal immune network for IgA production	6	145	46	7246	0.04137931	0.00634833	6.51814093	959;3558;3569;3565;3586;7040	CD40LG;IL2;IL6;IL4;IL10;TGFB1	0.00028037	0.00066419	Organismal Systems
path:hsa04540	Gap junction	8	145	88	7246	0.05517241	0.01214463	4.542946708	3356;5894;1950;2697;5578;5594;5579;1956	HTR2A;RAF1;EGF;GJA1;PRKCA;MAPK1;PRKCB;EGFR	0.00035284	0.00082783	Cellular Processes
path:hsa04211	Longevity regulating pathway	8	145	89	7246	0.05517241	0.01228264	4.491902363	581;6648;5468;207;7157;3643;5970;847	BAX;SOD2;PPARG;AKT1;TP53;INSR;RELA;CAT	0.00038123	0.00088591	Organismal Systems
path:hsa04371	Apelin signaling pathway	10	145	137	7246	0.06896552	0.01890698	3.647621445	5894;5294;4843;6696;207;4846;595;5594;5327;5054	RAF1;PIK3CG;NOS2;SPP1;AKT1;NOS3;CCND1;MAPK1;PLAT;SERPINE1	0.00039595	0.00091144	Environmental Information Processing
path:hsa04912	GnRH signaling pathway	8	145	93	7246	0.05517241	0.01283467	4.298702262	5894;1432;4313;3725;5578;5594;5579;1956	RAF1;MAPK14;MMP2;JUN;PRKCA;MAPK1;PRKCB;EGFR	0.00051401	0.00117215	Organismal Systems
path:hsa04150	mTOR signaling pathway	10	145	151	7246	0.06896552	0.02083908	3.309431377	5894;207;7124;5578;5594;3643;5728;2932;5579;1147	RAF1;AKT1;TNF;PRKCA;MAPK1;INSR;PTEN;GSK3B;PRKCB;CHUK	0.00085257	0.00192619	Environmental Information Processing
path:hsa00380	Tryptophan metabolism	5	145	40	7246	0.03448276	0.00552029	6.246551724	1543;1545;1544;4129;847	CYP1A1;CYP1B1;CYP1A2;MAOB;CAT	0.00112093	0.00250923	Metabolism
path:hsa04940	Type I diabetes mellitus	5	145	41	7246	0.03448276	0.00565829	6.094196804	3458;3558;3553;7124;3552	IFNG;IL2;IL1B;TNF;IL1A	0.00125632	0.00278674	Human Diseases
path:hsa04623	Cytosolic DNA-sensing pathway	6	145	62	7246	0.04137931	0.00855644	4.836040044	3627;3569;3553;4792;5970;1147	CXCL10;IL6;IL1B;NFKBIA;RELA;CHUK	0.00141571	0.00311201	Organismal Systems
path:hsa04217	Necroptosis	10	145	162	7246	0.06896552	0.02235716	3.084716901	581;3458;142;6772;3320;3553;7124;3552;596;841	BAX;IFNG;PARP1;STAT1;HSP90AA1;IL1B;TNF;IL1A;BCL2;CASP8	0.00145946	0.00317954	Cellular Processes
path:hsa04910	Insulin signaling pathway	9	145	137	7246	0.06206897	0.01890698	3.2828593	5894;3099;207;5770;31;6517;5594;3643;2932	RAF1;HK2;AKT1;PTPN1;ACACA;SLC2A4;MAPK1;INSR;GSK3B	0.00163675	0.00353422	Organismal Systems
path:hsa04725	Cholinergic synapse	8	145	112	7246	0.05517241	0.0154568	3.569458128	5294;43;207;2353;5578;5594;5579;596	PIK3CG;ACHE;AKT1;FOS;PRKCA;MAPK1;PRKCB;BCL2	0.00174525	0.00370296	Organismal Systems

Term_ID	Term_description	ListHit	ListTotal	PopHit	PopTotal	GeneRatio	BgRatio	Enrichment_score	GeneIds	GeneSymbols	p-value	FDR_bh	Classification_level1
path:hsa04670	Leukocyte transendothelial migration	8	145	112	7246	0.05517241	0.0154568	3.569458128	4318;1432;4313;3383;653361;5578;7412;5579	MMP9;MAPK14;MMP2;ICAM1;NCF1;PRKCA;VCAM1;PRKCB	0.00174525	0.00370296	Organismal Systems
path:hsa05131	Shigellosis	6	145	65	7246	0.04137931	0.00897047	4.612838196	1432;4792;5594;5970;1147;3576	MAPK14;NFKBIA;MAPK1;RELA;CHUK;CXCL8	0.0018112	0.00377719	Human Diseases
path:hsa04137	Mitophagy - animal	6	145	65	7246	0.04137931	0.00897047	4.612838196	3091;598;3725;7157;1869;5970	HIF1A;BCL2L1;JUN;TP53;E2F1;RELA	0.0018112	0.00377719	Cellular Processes
path:hsa04060	Cytokine-cytokine receptor interaction	14	145	292	7246	0.09655172	0.0402981	2.395937648	6347;959;3627;3569;3558;3458;3565;2920;3553;7124;3552;3586;7040;3576	CCL2;CD40LG;CXCL10;IL6;IL2;IFNG;IL4;CXCL2;IL1B;TNF;IL1A;IL10;TGFB1;CXCL8	0.00204735	0.0042335	Environmental Information Processing
path:hsa04930	Type II diabetes mellitus	5	145	46	7246	0.03448276	0.00634833	5.431784108	3099;7124;6517;5594;3643	HK2;TNF;SLC2A4;MAPK1;INSR	0.00212126	0.00434948	Human Diseases
path:hsa05310	Asthma	4	145	28	7246	0.02758621	0.0038642	7.138916256	7124;3586;959;3565	TNF;IL10;CD40LG;IL4	0.00216765	0.00440755	Human Diseases
path:hsa05031	Amphetamine addiction	6	145	68	7246	0.04137931	0.00938449	4.409330629	2353;3725;6531;5578;5579;4129	FOS;JUN;SLC6A3;PRKCA;PRKCB;MAOB	0.00228535	0.00460847	Human Diseases
path:hsa04072	Phospholipase D signaling pathway	9	145	145	7246	0.06206897	0.02001104	3.101736029	5294;5894;207;1950;5578;5594;3643;3576;1956	PIK3CG;RAF1;AKT1;EGF;PRKCA;MAPK1;INSR;CXCL8;EGFR	0.00241996	0.00483991	Environmental Information Processing
path:hsa04015	Rap1 signaling pathway	11	145	206	7246	0.07586207	0.02842948	2.668429863	4233;5894;207;1432;7422;1950;5578;5594;3643;5579;1956	MET;RAF1;AKT1;MAPK14;VEGFA;EGF;PRKCA;MAPK1;INSR;PRKCB;EGFR	0.00271817	0.00539215	Environmental Information Processing
path:hsa04913	Ovarian steroidogenesis	5	145	49	7246	0.03448276	0.00676235	5.099225897	1543;1545;5743;240;3643	CYP1A1;CYP1B1;PTGS2;ALOX5;INSR	0.00281284	0.00553493	Organismal Systems
path:hsa04520	Adherens junction	6	145	72	7246	0.04137931	0.00993652	4.164367816	4233;5770;5594;3643;2064;1956	MET;PTPN1;MAPK1;INSR;ERBB2;EGFR	0.00305624	0.00591843	Cellular Processes
path:hsa00980	Metabolism of xenobiotics by cytochrome P450	6	145	72	7246	0.04137931	0.00993652	4.164367816	2944;1543;1576;1545;1544;2950	GSTM1;CYP1A1;CYP3A4;CYP1B1;CYP1A2;GSTP1	0.00305624	0.00591843	Metabolism
path:hsa04611	Platelet activation	8	145	123	7246	0.05517241	0.01697488	3.250238295	5294;5742;207;1432;4846;1277;5594;1281	PIK3CG;PTGS1;AKT1;MAPK14;NOS3;COL1A1;MAPK1;COL3A1	0.00314501	0.00604239	Organismal Systems
path:hsa00232	Caffeine metabolism	2	145	5	7246	0.0137931	0.00069004	19.98896552	7498;1544	XDH;CYP1A2	0.00382262	0.00724188	Metabolism
path:hsa04650	Natural killer cell mediated cytotoxicity	8	145	127	7246	0.05517241	0.01752691	3.147868585	5894;3458;3383;7124;5578;5594;5579;836	RAF1;IFNG;ICAM1;TNF;PRKCA;MAPK1;PRKCB;CASP3	0.0038287	0.00724188	Organismal Systems
path:hsa04140	Autophagy - animal	8	145	128	7246	0.05517241	0.01766492	3.123275862	3091;5894;598;207;1509;5594;5728;596	HIF1A;RAF1;BCL2L1;AKT1;CTSD;MAPK1;PTEN;BCL2	0.00401635	0.00753839	Cellular Processes
path:hsa04923	Regulation of lipolysis in adipocytes	5	145	54	7246	0.03448276	0.00745239	4.627075351	5742;207;5743;3643;154	PTGS1;AKT1;PTGS2;INSR;ADRB2	0.00430746	0.00802306	Organismal Systems
path:hsa04728	Dopaminergic synapse	8	145	131	7246	0.05517241	0.01807894	3.051750461	207;1432;2353;6531;5578;5579;2932;4129	AKT1;MAPK14;FOS;SLC6A3;PRKCA;PRKCB;GSK3B;MAOB	0.00462223	0.00854413	Organismal Systems
path:hsa05010	Alzheimer disease	9	145	164	7246	0.06206897	0.02263318	2.742388562	351;841;840;3553;7124;842;5594;2932;836	APP;CASP8;CASP7;IL1B;TNF;CASP9;MAPK1;GSK3B;CASP3	0.0054859	0.01006436	Human Diseases
path:hsa04960	Aldosterone-regulated sodium reabsorption	4	145	37	7246	0.02758621	0.00510627	5.402423113	5594;5578;3643;5579	MAPK1;PRKCA;INSR;PRKCB	0.00608487	0.01107991	Organismal Systems
path:hsa04213	Longevity regulating pathway - multiple species	5	145	62	7246	0.03448276	0.00855644	4.03003337	6648;207;6647;3643;847	SOD2;AKT1;SOD1;INSR;CAT	0.007761	0.01402728	Organismal Systems
path:hsa04261	Adrenergic signaling in cardiomyocytes	8	145	144	7246	0.05517241	0.01987303	2.776245211	5294;207;1432;6331;5578;5594;154;596	PIK3CG;AKT1;MAPK14;SCN5A;PRKCA;MAPK1;ADRB2;BCL2	0.00809567	0.01452459	Organismal Systems
path:hsa04666	Fc gamma R-mediated phagocytosis	6	145	90	7246	0.04137931	0.01242065	3.331494253	5894;207;653361;5578;5594;5579	RAF1;AKT1;NCF1;PRKCA;MAPK1;PRKCB	0.00909129	0.01619178	Organismal Systems
path:hsa04152	AMPK signaling pathway	7	145	120	7246	0.04827586	0.01656086	2.915057471	5468;207;595;31;6517;3643;890	PPARG;AKT1;CCND1;ACACA;SLC2A4;INSR;CCNA2	0.01008854	0.0178377	Environmental Information Processing
path:hsa04020	Calcium signaling pathway	9	145	182	7246	0.06206897	0.02511731	2.471163319	3356;4843;4846;2065;5578;5579;154;2064;1956	HTR2A;NOS2;NOS3;ERBB3;PRKCA;PRKCB;ADRB2;ERBB2;EGFR	0.0105908	0.01859104	Environmental Information Processing

Term_ID	Term_description	ListHit	ListTotal	PopHit	PopTotal	GeneRatio	BgRatio	Enrichment_score	GeneIds	GeneSymbols	p-value	FDR_bh	Classification_level1
path:hsa04973	Carbohydrate digestion and absorption	4	145	44	7246	0.02758621	0.00607232	4.542946708	8972;3099;5579;207	MGAM;HK2;PRKCB;AKT1	0.01123192	0.01957563	Organismal Systems
path:hsa00982	Drug metabolism - cytochrome P450	5	145	68	7246	0.03448276	0.00938449	3.674442191	2944;1576;1544;2950;4129	GSTM1;CYP3A4;CYP1A2;GSTP1;MAOB	0.01136814	0.01967253	Metabolism
path:hsa04916	Melanogenesis	6	145	101	7246	0.04137931	0.01393872	2.968658245	5894;5578;5594;5579;2932;7299	RAF1;PRKCA;MAPK1;PRKCB;GSK3B;TYR	0.01549152	0.02661923	Organismal Systems
path:hsa05030	Cocaine addiction	4	145	49	7246	0.02758621	0.00676235	4.079380718	3725;5970;6531;4129	JUN;RELA;SLC6A3;MAOB	0.01623937	0.02751671	Human Diseases
path:hsa00330	Arginine and proline metabolism	4	145	49	7246	0.02758621	0.00676235	4.079380718	4129;4953;4843;4846	MAOB;ODC1;NOS2;NOS3	0.01623937	0.02751671	Metabolism
path:hsa05320	Autoimmune thyroid disease	4	145	50	7246	0.02758621	0.00690036	3.997793103	3586;3558;959;3565	IL10;IL2;CD40LG;IL4	0.01738402	0.02925311	Human Diseases
path:hsa04024	cAMP signaling pathway	9	145	199	7246	0.06206897	0.02746343	2.260058915	5894;207;2353;3725;4792;5594;154;5970;5465	RAF1;AKT1;FOS;JUN;NFKBIA;MAPK1;ADRB2;RELA;PPARA	0.01811682	0.03027743	Environmental Information Processing
path:hsa00983	Drug metabolism - other enzymes	5	145	77	7246	0.03448276	0.01062655	3.244961935	2944;7498;1576;2950;4353	GSTM1;XDH;CYP3A4;GSTP1;MPO	0.01869269	0.03102733	Metabolism
path:hsa00052	Galactose metabolism	3	145	31	7246	0.02068966	0.00427822	4.836040044	8972;3099;231	MGAM;HK2;AKR1B1	0.02343149	0.03863029	Metabolism
path:hsa04146	Peroxisome	5	145	83	7246	0.03448276	0.0114546	3.010386373	6648;4843;7498;6647;847	SOD2;NOS2;XDH;SOD1;CAT	0.02499527	0.04093185	Cellular Processes
path:hsa04350	TGF-beta signaling pathway	5	145	84	7246	0.03448276	0.0115926	2.97454844	3458;4609;7124;5594;7040	IFNG;MYC;TNF;MAPK1;TGFB1	0.02616322	0.04255884	Environmental Information Processing
path:hsa04310	Wnt signaling pathway	7	145	146	7246	0.04827586	0.02014905	2.395937648	3725;7157;595;4609;5578;5579;2932	JUN;TP53;CCND1;MYC;PRKCA;PRKCB;GSK3B	0.02686841	0.04341651	Environmental Information Processing
path:hsa00140	Steroid hormone biosynthesis	4	145	60	7246	0.02758621	0.00828043	3.331494253	1543;1545;1576;1544	CYP1A1;CYP1B1;CYP3A4;CYP1A2	0.03161516	0.05041895	Metabolism
path:hsa04730	Long-term depression	4	145	60	7246	0.02758621	0.00828043	3.331494253	5578;5894;5594;5579	PRKCA;RAF1;MAPK1;PRKCB	0.03161516	0.05041895	Organismal Systems
path:hsa05016	Huntington disease	8	145	187	7246	0.05517241	0.02580734	2.137857275	581;6648;5468;7157;6647;842;836;841	BAX;SOD2;PPARG;TP53;SOD1;CASP9;CASP3;CASP8	0.03365835	0.05332882	Human Diseases
path:hsa04390	Hippo signaling pathway	7	145	154	7246	0.04827586	0.02125311	2.271473354	332;595;4609;11186;2932;5054;7040	BIRC5;CCND1;MYC;RASSF1;GSK3B;SERPINE1;TGFB1	0.03453068	0.05435798	Environmental Information Processing
path:hsa05217	Basal cell carcinoma	4	145	63	7246	0.02758621	0.00869445	3.172851669	581;1026;7157;2932	BAX;CDKN1A;TP53;GSK3B	0.03690441	0.05772228	Human Diseases
path:hsa04640	Hematopoietic cell lineage	5	145	94	7246	0.03448276	0.01297267	2.658107117	3569;3565;3553;7124;3552	IL6;IL4;IL1B;TNF;IL1A	0.03977771	0.06182013	Organismal Systems
path:hsa04720	Long-term potentiation	4	145	67	7246	0.02758621	0.00924648	2.983427689	5578;5894;5594;5579	PRKCA;RAF1;MAPK1;PRKCB	0.04470393	0.0686732	Organismal Systems
path:hsa04022	cGMP-PKG signaling pathway	7	145	163	7246	0.04827586	0.02249517	2.14605458	5294;5894;207;4846;5594;3643;154	PIK3CG;RAF1;AKT1;NOS3;MAPK1;INSR;ADRB2	0.04475016	0.0686732	Environmental Information Processing
path:hsa04750	Inflammatory mediator regulation of TRP channels	5	145	99	7246	0.03448276	0.01366271	2.523859282	3356;1432;3553;5578;5579	HTR2A;MAPK14;IL1B;PRKCA;PRKCB	0.04794368	0.07311411	Organismal Systems
